# Metal Complexes Containing Homoleptic Diorganoselenium(II) Ligands: Synthesis, Characterization and Investigation of Optical Properties

**DOI:** 10.3390/molecules29040792

**Published:** 2024-02-08

**Authors:** Darius Dumitraș, Emese Gal, Cristian Silvestru, Alexandra Pop

**Affiliations:** 1 Supramolecular Organic and Organometallic Chemistry Centre, Department of Chemistry, Faculty of Chemistry and Chemical Engineering, Babeş-Bolyai University, Str. Arany Janos 11, RO-400028 Cluj-Napoca, Romania; darius.dumitras@ubbcluj.ro (D.D.); cristian.silvestru@ubbcluj.ro (C.S.); 2 Research Center on Fundamental and Applied Heterochemistry, Faculty of Chemistry and Chemical Engineering, Babeș-Bolyai University, Str. Arany Janos 11, RO-400028 Cluj-Napoca, Romania; emese.gal@ubbcluj.ro

**Keywords:** homoleptic diorganoselenium(II) proligands, oxazolone, metal complexes, solution behavior, X-ray structures, supramolecular interactions, photophysical properties

## Abstract

[(*Z*)-2′-{*2*-C_6_H_5_-(4*H*)-oxazol-5-one}CHC_6_H_4_]_2_Se (**5**, L^1^) and [(*Z*)-4′-{2-C_6_H_5_-(4*H*)-oxazol-5-one}CHC_6_H_4_]_2_Se (**6**, L^2^) were prepared, structurally characterized and used as ligands to obtain new metal complexes of types [MX(L^n^)] [L^1^: M = Ag, X = OTf (**7**); M = Au, X = Cl (**13**); L^2^: M = Ag, X = OTf (**8**); M = Au, X = Cl (**14**)], [(MX)_2_(L^n^)] [M = Ag, X = OTf, L^1^ (**9**); L^2^ (**10**)], [ZnCl_2_(L^n^)] [L^1^ (**15**); L^2^ (**16**)] and [Ag(L^n^)][PF_6_] [L^1^ (**11**); L^2^ (**12**)]. The silver complexes **7** and **8** were ionic species (1:1 electrolytes) in a MeCN solution, while in the solid state, the triflate fragments were bonded to the silver cations. Similarly, the 2:1 complexes **9** and **10** were found to behave as 1:2 electrolytes in a MeCN solution, but single-crystal X-ray diffraction demonstrated that compound **9** showed the formation of a dimer in the solid state: a tetranuclear [Ag(OTf)]_4_ built through bridging triflate ligands was coordinated by two bridging organoselenium ligands through the nitrogen from the oxazolone ring and the selenium atoms in a 1κN:2κSe fashion. Supramolecular architectures supported by intermolecular C−H∙∙∙π, C−H∙∙∙O, Cl∙∙∙H and F∙∙∙H interactions were observed in compounds **4**, **5** and **9**. The compounds exhibited similar photophysical properties, with a bathochromic shift in the UV-Vis spectra caused by the position of the oxazolone ring on the phenyl ring attached to the selenium atoms.

## 1. Introduction

Organoselenium chemistry is a growing field of research not only due to the application of organoselenium compounds in biology [1], material science [2] and organic chemistry [3] but also for their use in coordination chemistry as ligands for the preparation of metal complexes [4].

The antioxidant properties of organoselenium compounds have determined the development of selenium fluoroprobes for the investigation of cell functions and the recognition of metal cations [5,6]. One example is Rhodamine B selenolactone, which has been found to have fluorescence on–off responses to Ag^+^ and Hg^2+^ cations due to the affinity of selenium for silver and mercury [7].

Diorganoselenium compounds with homoleptic or heteroleptic organic groups containing more than one donor atom in their skeletons are fascinating and interesting to study because of their potential use for the coordination of *soft* and *hard* metal ions due both to the softness properties of selenium atoms and the hardness of other donor atoms, such as nitrogen. Metal complexes containing multidentate diorganoselenides have been found to have applications in medicine [8] and material science [9]. For the preparation of such species, the role of ligands is very important. To prepare potentially multidentate proligands, the geometrical properties of the binding sites can be rationalized considering the stereoelectronic preferences of the targeted metals. The incorporation of several donor atoms, both *soft* and *hard*, in the structure of a proligand represents a challenge as different types of coordination patterns to the metal cations can be accomplished. A series of biologically active selenium-containing compounds have been used to obtain complexes with metal cations known for their importance in biology (e.g., selenonicotine derivatives were used as linkers to build 1D coordination polymers [10]) and material science (e.g., dichlorobis [1,3-dimethyl-2(3*H*)-imidazoleselone]zinc(II) was used as a ZnSe precursor [11]).

For years, our research interests have been focused on the synthesis and structural characterization of metal complexes with organochalcogen multidentate ligands for potential applications in optoelectronics and material science, e.g., complexes of heteroleptic organochalcogen ligands containing nitrogen and chalcogen donor atoms with coinage metals [12,13,14], group 12 [15,16] and tetrels [17]. In the attempt to obtain metal complexes with photophysical properties and potential applications in optoelectronics, in the present study, we designed new homoleptic diarylselenides with organic substituents containing oxazolone fragments in their skeletons as ligands to build metal complexes. The selection of the oxazolone moiety was based on the optical properties described for various compounds containing such heterocycles [18,19,20,21,22]. In a previous work, we described the related heteroleptic diorganoselenide (*n*-Bu)[(*Z*)-4′-{2-C_6_H_5_-(4*H*)-oxazol-5-one}CHC_6_H_4_]Se and used it for the synthesis of silver(I) complexes [23]. The metal salts used for the synthesis of complexes in the present work were chosen considering their abilities to provide ionic or neutral metal complexes with potential luminescence properties. Silver triflate was selected due to the capacity of triflate anions to behave as bridging ligands, thus being very useful building blocks for the construction of coordination polymers [23] or different heteropolynuclear complexes (e.g., [Hg(mesityl)_2_Ag_2_(OTf)_2_]_2_ [24]).

## 2. Results and Discussion

### 2.1. Synthesis

**Scheme 1:**
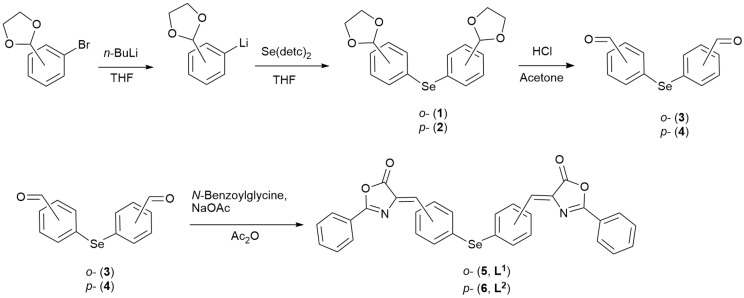
Synthesis of diorganoselenium(II) compounds **1**–**6**.

The precursor [2-(O=CH)C_6_H_4_]_2_Se (**3**) was prepared following the method previously described in the literature [25], while for [4-(O=CH)C_6_H_4_]_2_Se (**4**), the method was adapted [26]. Even though the diorganoselenide **4** has previously been reported in the literature [27], we developed a new method for the synthesis of this compound and fully characterized it. Thus, a THF solution of the freshly protected 4-brombenzyl aldehyde, 4-[(CH_2_O)_2_CH]C_6_H_4_Br, was treated with n-BuLi at −78 °C, in THF. After 3 h, Se(detc)_2_ (detc = diethyl dithiocarbamate) was added to the reaction mixture, and the mixture was allowed to reach room temperature. After the isolation and purification of {4-[(CH_2_O)_2_CH]C_6_H_4_}_2_Se (**2**), the deprotection of the aldehyde fragments was achieved by treatment with HCl in acetone to give the desired precursor **4** (Scheme 1).

The target compounds [(*Z*)-2′-{2-C_6_H_5_-(4*H*)-oxazol-5-one}CHC_6_H_4_]_2_Se (**5**, **L^1^**) and [(*Z*)-4′-{2-C_6_H_5_-(4*H*)-oxazol-5-one}CHC_6_H_4_]_2_Se (**6**, **L^2^**) were obtained using a well-known literature procedure, i.e., the Erlenmeyer–Plochl method [28,29,30,31], by reacting [2-(O=CH)C_6_H_4_]_2_Se (**3**) and [4-(O=CH)C_6_H4]_2_Se (**4**), respectively, with *N*-benzoylglycine and sodium acetate, in acetic anhydride, at 100 °C for 2 h (Scheme 1). After the work-up of the reaction mixtures, the products were isolated in very good yields (75% for **5** and 92% for **6**) as intense yellow solids. As in most of the cases, using the described procedure, only the *Z* isomer was isolated for both oxazolone-containing compounds **5** and **6** [32].

The ability of **5** and **6** to behave as ligands for metal cations was investigated towards the following salts: AgOTf, AgPF_6_, AuCl(tht) and ZnCl_2_. The proligand-to-metal molar ratio used for the reactions was 1:1. For reactions with AgOTf, a proligand-to-metal molar ratio of 1:2 was also used in order to study the influence of the amount of metal salts towards complexation and taking into account the potential multidenticity of proligands **5** and **6**.

**Scheme 2:**
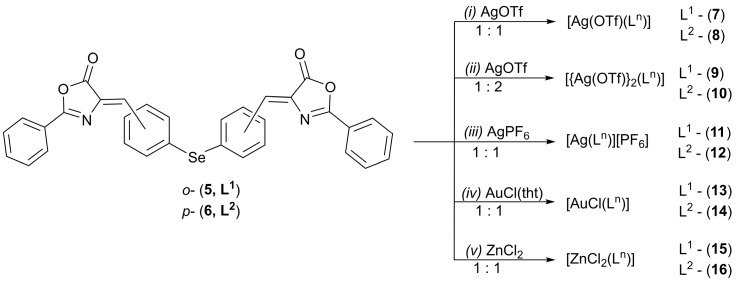
Synthesis of complexes **7**–**16**: *(i)* and *(ii)* acetone; *(iii)* and *(iv)* CH_3_Cl; *(**v**)* CH_3_Cl/EtOH.

All the complexes were prepared at room temperature, under normal atmospheric conditions, using dry solvents (acetone for **7**–**10**, CH_3_Cl for **11**–**14**, and a mixture of CH_3_Cl/EtOH for **15**–**16**) (Scheme 2). The reaction time was 30 min, and the preparation and work-up of the silver complexes **7**–**12** was performed in the absence of light, to avoid decomposition. The metal complexes **7**–**16** were isolated as intense colored solids, in very good yields, after precipitation with Et_2_O (compounds **7**–**12**) and *n*-hexane (compounds **13**–**16**) and washing the precipitates several times, with the same solvent.

The proligands **5** and **6** and the new metal complexes were characterized in the solid state by IR spectroscopy and in solution by multinuclear NMR spectroscopy, mass spectrometry and molar conductivities (for metal complexes). The optical properties of the compounds were investigated in solution by UV-Vis and fluorescence measurements. The solid-state molecular structures of **4**, **5** and **9** were obtained by single-crystal X-ray diffraction.

### 2.2. Spectroscopic Characterization

The FT-IR spectrum of compound **4** exhibits a typical strong band at 1710 cm^−1^ for the C=O stretching vibration, consistent with the deprotection of the aldehyde fragments in the precursor **2**. The formation of the oxazolone derivatives **5** and **6** according to Scheme 1 was confirmed by their infrared spectra, i.e., *(i)* the disappearance of a NH absorption band around 3400–3500 cm^−1^, which is present in the *N*-benzoylglycine used as starting reagent [33], *(ii)* the presence in the range 1768–1795 cm^−1^ of a characteristic double absorption, due to Fermi resonance, for the C=O stretching vibration in the lactone group [34,35,36], and *(iii)* the presence of bands for stretching of endocyclic C=N bonds (1651 and 1653 cm^−1^ for **5** and **6**, respectively) and C−O (1289/1225 and 1295/1238 cm^−1^ for **5** and **6**, respectively) [35,37]. The IR spectra of the metal complexes **7**–**16** exhibit bands for stretching vibrations of the organoselenium ligands in the same ranges as observed for free, uncoordinated **5** and **6**, i.e., 1750–1800 cm^−1^, 1650–1660 cm^−1^ and 1220–1300 cm^−1^ for ν(C=O), ν(C=N) and ν(C−O) stretching vibrations, respectively.

In the IR spectra of the silver complexes **7**–**10**, the four characteristic bands for the triflate fragment were found in the range 1040–1330 cm^−1^, including the very strong split band corresponding to the asymmetric SO_3_ stretch at 1289 cm^−1^ in **7**, 1231 cm^−1^ in **8**, 1296 cm^−1^ in **9** and 1291 cm^−1^ in **10**. The splitting of these strong bands indicates an interaction, in the solid state, of the triflate fragment with the silver atom [38].

**Scheme 3:**
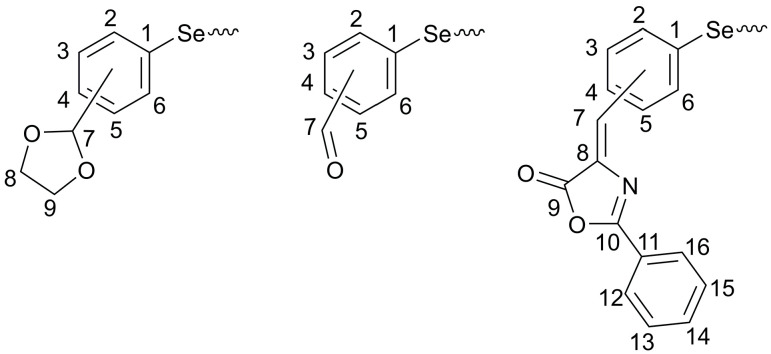
Numbering scheme for NMR assignments in compounds **1**–**16**.

Multinuclear NMR spectroscopy was employed to characterize all the compounds in solution, at room temperature. The ^1^H and ^13^C{^1^H} NMR spectra of the diorganoselenides **1**−**6** exhibited only one set of resonances, consistent with the formation of only one species in solution, with equivalent organic groups attached to selenium atoms. All resonances showed the expected multiplicity and were assigned using 2D NMR spectra (according to the numbering scheme displayed in Scheme 3). Two multiplet ^1^H resonances in the range δ 4.00–4.20 ppm were observed for the protons of the dioxolane ring in compounds **1** and **2**. Their complete disappearance in the spectra of compounds **3** and **4**, together with the change in the chemical shift for the resonance assigned to H7 protons from δ 6.14 ppm in **1** to 10.28 ppm in **3** and from δ 5.8 ppm in **2** to 9.98 ppm in **4**, evidenced the formation of the target species by deprotection of the aldehyde moieties. The formation of the oxazolone ring in **5** and **6** was easily confirmed by the shift of the singlet resonance for H7 protons to δ 7.85 ppm in **5** (see Supplementary Material, Figure S1) and δ 7.22 ppm in **6** (see Supplementary Material, Figure S2). Differences in the chemical shifts and in the multiplicity of the resonance signals were observed in complexes **7**–**16** when compared with the free proligands **5** and **6** (see Supplementary Material, Figures S3 and S4).

The ^13^C{^1^H} NMR spectra showed the expected resonances for all compounds, slightly shifted in the metal complexes when compared with the free proligands.

The ^77^Se{^1^H} NMR spectra were recorded for all the compounds; nonetheless, in some metal complexes, the signals were not observed. The singlet ^77^Se{^1^H} resonance signals are in the range δ = 320–443 ppm, corresponding to the typical chemical shifts for diorganoselenides, as reported in the literature [39,40]. The chemical shifts in the proligands **5** (δ = 343 ppm) and **6** (δ = 438 ppm) are very similar to those observed for the metal complexes **13** (δ = 343 ppm), **14** (δ = 437 ppm) and **16** (δ = 437 ppm), indicating that Se–M (M = Ag, Au and Zn) interactions might not be present in solution. This is consistent with the previously observed behavior for [Ag{(*n*-Bu)[4-{2-C_6_H_5_-(4*H*)-oxazol-5-one}CHC_6_H_4_]Se}][X] (X = OTf, PF_6_) [23], in solution, when only the nitrogen atoms from the oxazolone rings are coordinated to the metal center.

The silver complexes **7**–**12** exhibited one resonance signal in the ^19^F{^1^H} and ^31^P{^1^H} NMR spectra, with the expected multiplicities, thus being consistent with the presence of only one species in solution.

When the metal was changed, from Ag(I) to Au(I) and Zn(II), no major differences were observed in the NMR spectra recorded in solution for the complexes reported in this work. Based on the NMR spectra of the complexes reported herein and taking into account the solid-state molecular structure for **9** (vide infra), for the complex [Ag(OTf){(*n*-Bu)[(*Z*)-4′-{2-C_6_H_5_-(4*H*)-oxazol-5-one}CHC_6_H_4_]S}], which contains a related heteroleptic diorganosulfide ligand [23], as well as other literature data [40], we suggest that the metal ion in complexes **7**–**16** is coordinated by the nitrogen atom of an oxazolone ring. The presence of only one set of resonances for the organic groups attached to the selenium atom in the NMR spectra of the complexes **7**, **8**, **11**–**16** is consistent with a dynamic behavior in solution, at room temperature, the metal atom being alternatively attached to one of the two organic substituents from a selenium ligand. To investigate such a dynamic behavior, as also proposed for the related mercury(II) complex [HgCl_2_{2-(4,4-dimethyl-2-oxazolinyl)phenyl}_2_Se] [40], a VT NMR study in acetone-*d*_6_ was performed for compound **7**. Unfortunately, the poor solubility of the complex prevented measurements carried out below −50 °C, and the spectra recorded until this temperature provided no evidence regarding the presence of non-equivalent organic substituents on selenium.

The HRMS ESI+ spectra show the molecular peak in **1** (*m*/*z* 290.99188), **5** (*m*/*z* 577.06329) and **6** (*m*/*z* 577.06671) and the cations [M-OTf]^+^ (*m*/*z* 682.96344 for **7**, *m*/*z* 682.96337 for **8**) and [M-PF_6_]^+^ (*m*/*z* 682.96246 for **11**, *m*/*z* 682.96350 for **12**) as the base peak.

Molar conductivity measurements were performed in 10^−3^ M acetonitrile solution. The Λ_M_ values in the range 120–170 Ω^−1^·cm^2^·mol^−1^ found for the complexes **7**, **8**, **11** and **12** are consistent with a behavior as 1:1 electrolytes, while Λ_M_ values in the range 14–65 Ω^−1^·cm^2^·mol^−1^, observed for the gold complexes **13** and **14** as well as for zinc complexes **15** and **16**, indicate their non-electrolyte nature [41,42]. The Λ_M_ values found for complexes **9** (250 Ω^−1^·cm^2^·mol^−1^) and **10** (230 Ω^−1^·cm^2^·mol^−1^) confirm their behavior as 1:2 (cation–anion) electrolytes in solution.

**Figure 1:**
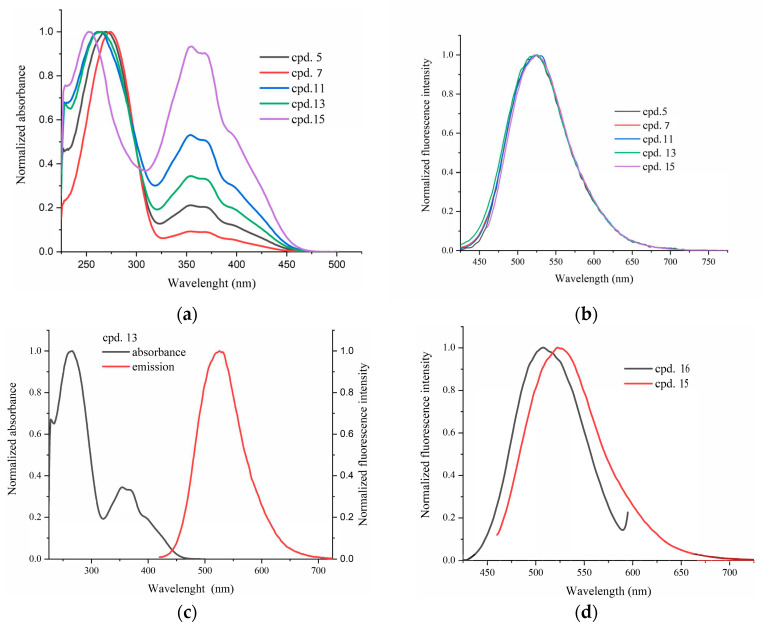
(**a**) Normalized UV–Vis absorption spectra of **5**, **7**, **11**, **13** and **15**; (**b**) normalized emission spectra of **5**, **7**, **11**, **13** and **15**; (**c**) normalized absorption and emission spectra of **13**, and (**d**) normalized emission spectra of **15** and **16**.

The photophysical investigation of the homoleptic diorganoselenium compounds functionalized with oxazolone and their complexes with different *d* block metals was carried out at room temperature, in 5·10^−5^ M dichloromethane solution. The relevant data obtained from this investigation are summarized in Table 1. Figure 1a–d as well as Figures S5 and S6 (see Supplementary Material) show the absorption and emission spectra of these compounds.

The UV-Vis absorption spectra of compounds **5**–**16** revealed the electron-donor property of the oxazolone ring by intraligand charge transfer (ILCT) characterized by large molar extinction coefficients. The presence of the oxazolone ring in the skeleton of the compounds was easily evidenced by the presence of a higher energy band in the visible region for compounds **5**–**16**, when compared to precursors **3** and **4**. The intensive absorption bands below 300 nm in **3** and **4** were assigned to intraligand π–π* interactions [43].

The pattern of the emission in the spectra of the metal complexes resembles that of the free ligands, and the intensity of the emission is not modified (Figure 1b). This emission behavior suggests the absence of M···M interaction in solution. The perturbation caused by this type of interactions is usually reflected in a pronounced luminescence, as was revealed in several silver complexes, i.e., [Ag{4-(4-*N*,*N*-dimethylaminophenyl)-2,6-diphenylpyridine}_2_][ClO_4_] [44], [Ag_4_(SO_4_)_2_(3,3′,5,5′-tetramethyl-4,4′-bipyrazole)_4_]·H_2_O, [Ag_2_(SO_4_)(3,3′,5,5′-tetramethyl-4,4′-bipyrazole)_2_]·3H_2_O [43], [Ag(ClO_4_)(tris[2-(2-pyridyl)ethyl]phosphine)] and [Ag_4_Cl_4_(tris[2-(2-pyridyl)ethyl]phosphine)_2_] [45].

It is worth mentioning that both the compounds containing the *ortho*- (**5**, **7**, **11**, **13** and **15**) and the *para*-substituted (**6**, **8**, **12**, **14** and **16**) organic substituents attached to selenium showed quite similar photophysical behavior (Figure 1a,b); i.e., structured absorption spectra with maxima located between 240 and 275 nm and another intense absorption band between 300 and 400 nm can be observed for both sets of compounds. Nonetheless, the oxazolone moiety position on the phenyl ring attached to selenium has a significant effect on the absorbance of the compounds; i.e., the *para*-substituted compound **6** induced a bathochromic shift in the UV-Vis spectra (see Supplementary Material, Figure S5). A hyperchromic shift can be observed in the case of zinc complex **15** (Figure 1d). Despite the vibronic structure of the UV-Vis spectra, the emission spectra showed broad structureless maxima in the blue-green region, characterized by a relatively large Stokes shift. Similar emission maxima location was observed for all compounds, except compounds **8** and **14**, which do not present fluorescence emission.

### 2.3. Single-Crystal X-ray Diffraction Studies

The solid-state structure of the precursor **4**, the proligand **5** and the silver complex **9** was investigated by single-crystal X-ray diffraction.

**Figure 2:**
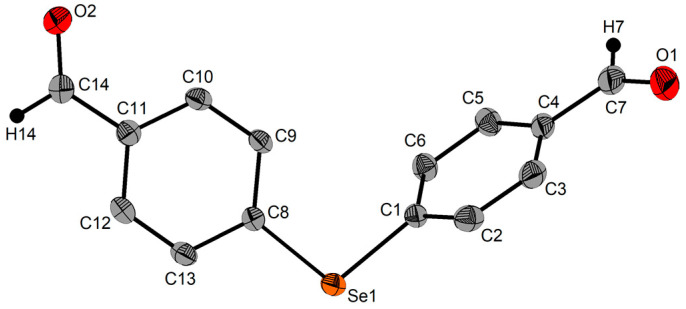
Thermal ellipsoid (probability 50%) representation of the molecular structure of compound **4**. Hydrogen atoms, except those of the aldehyde substituents, were omitted for clarity. Selected bond lengths (Å) and angles (°): Se1‒C1 1.916(2); Se1‒C8 1.907(2); C7‒O1 1.182(3); C14‒O2 1.220(3); C1‒Se1‒C8 101.15(9).

The molecule of compound **4** exhibits a bent geometry at the selenium atom with a C1‒Se1‒C8 angle of 101.15(9)° (Figure 2). The organic substituents attached to the central chalcogen atom are basically planar (deviations from the best [4-(O=CH)C_6_H_4_]Se plane in the range between −0.166 Å for O1 and 0.149 Å for C2 atoms for plane 1 and between −0.108 Å for C9 and 0.153 Å for O2 atoms for plane 2), with a dihedral angle of 70.5° between the two corresponding best planes.

**Figure 3:**
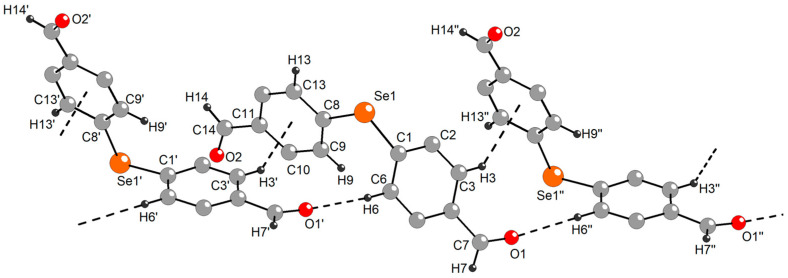
Polymeric chain association in the crystal of **4** [symmetry equivalent positions (−1/2+x, 3/2−y, −1/2+z) and (1/2+x, 3/2−y, 1/2+z) are given by “prime” and “double prime”, respectively]. Only the hydrogen atoms of the aldehyde substituents and those involved in intermolecular interactions are shown.

A closer check in the packing of the crystal revealed the formation of a polymeric chain association built from molecules of **4** doubly bridged through C−H_aryl_···π (Ph_centroid_) (H3···Ph_centroid_{C8″–C13″} 2.87 Å, *γ* = 6.6°; cf. H···Ar_centroid_ contacts shorter than 3.1 Å and an angle γ between the normal to the aromatic ring and the line defined by the H atom and Ar_centroid_ smaller than 30° [46]) and C−H_aryl_···O (H6···O1′ 2.58 Å; cf. Σr_vdW_(O,H) 2.70 Å [47]) interactions (Figure 3). Further hydrogen contacts between parallel chains involving both O1 and O2 atoms give rise to a 3D network (O1∙∙∙H9a 2.49 Å and O2∙∙∙H13b 2.46 Å) (see Supplementary Material, Figure S7).

**Figure 4:**
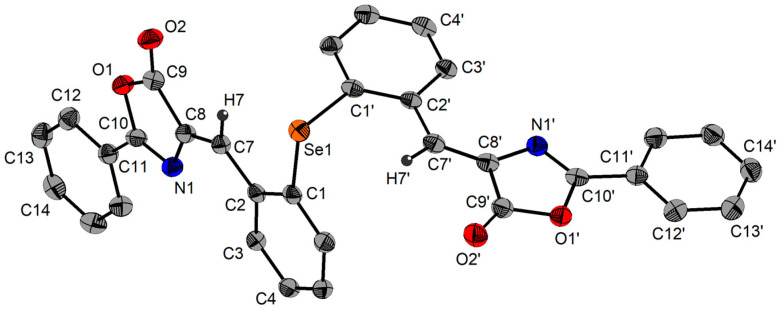
Thermal ellipsoid (probability 50%) representation of the molecular structure of compound **5** [symmetry equivalent position (−x, 1−y, z) is given by “prime”]. Hydrogen atoms, except those attached to the C7 and C7ʹatoms, were omitted for clarity. Selected bond lengths (Å) and angles (°): Se1‒C1 1.946(3); O1‒C9 1.399(4); O1‒C10 1.385(4); O2‒C9 1.193(4); N1‒C8 1.401(4); N1‒C10 1.295(4); C2‒C7 1.453(4); C7‒C8 1.354(4); C8‒C9 1.474(4); C1‒Se1‒C1’ 96.02(18); O1‒C9‒C8 104.5(3); C9‒O1‒C10 105.6(2); O1‒C10‒N1 116.0(3); C8‒N1‒C10 105.2(3); N1‒C8‒C9 108.7(3); C7‒C8‒C9 121.7(3); O2‒C9‒C8 133.6(3); O1‒C9‒O2 122.0(3).

In compound **5**, the angular geometry around the selenium atom is reflected by a C1‒Se1‒C1′ angle of 96.02(18)°, with half of the molecule generated by symmetry (Figure 4). The organic substituents on selenium are again planar (deviations of atoms from the best plane of a [2′-{2-C_6_H_5_-(4*H*)-oxazol-5-one}CHC_6_H_4_]Se fragment range between −0.086 Å for C16 atom and 0.117 Å for O2 atom) and almost orthogonal to each other (dihedral angle of 88.4° between the two best planes). The packing of the molecules in the crystal revealed a 2D supramolecular architecture formed by weak C−H_aryl_···O (H13···O2b 2.68 Å) intermolecular interactions involving a hydrogen from a phenyl moiety and the exocyclic oxygen from the oxazolone ring of a neighboring molecule (see Supplementary Material, Figure S8).

**Figure 5:**
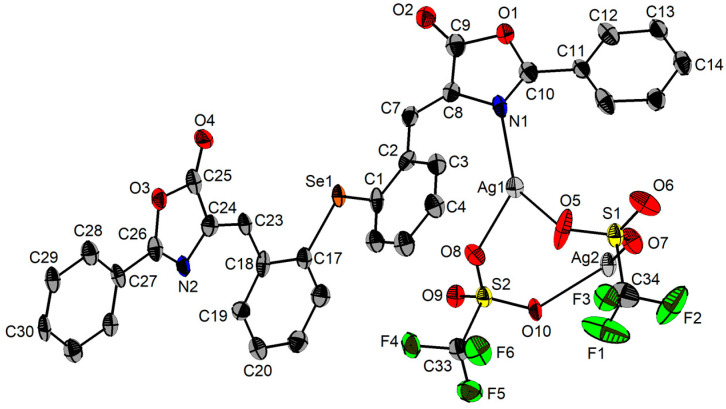
Thermal ellipsoid (probability 50%) representation of the molecular structure of **9**·4CHCl_3_ in the asymmetric unit. Hydrogen atoms and the molecules of CHCl_3_ were omitted for clarity.

Single crystals of the silver complex **9**, obtained when the proligand **5** was reacted with AgOTf in a 1:2 molar ratio, were isolated from a chloroform solution. Table 2 displays the most important interatomic distances and bond angles for **9**. The asymmetric unit of compound **9** contains one diorganoselenium ligand coordinated only through the nitrogen atom from one oxazole ring to one of the silver atoms of the dinuclear [Ag_2_(OTf)_2_] core (N1−Ag1 2.266(8)Å; cf. Σr_vdW_(Ag,N) 4.25 Å [47], Σr_cov_(Ag,N) 2.16 Å [48]) (Figure 5) and four molecules of crystallization solvent (CHCl_3_). The two metal atoms are bridged by the triflate anions generating an eight-membered Ag_2_O_4_S_2_ ring (Ag1−O5 2.254(7) Å, Ag1−O8 2.359(6) Å and Ag2−O7 2.336(6)Å, Ag2−O10 2.341(6)Å, respectively; cf. Σr_vdW_(Ag,O) 4.09 Å [47], Σr_cov_(Ag,O) 2.11 Å [48]).

**Figure 6:**
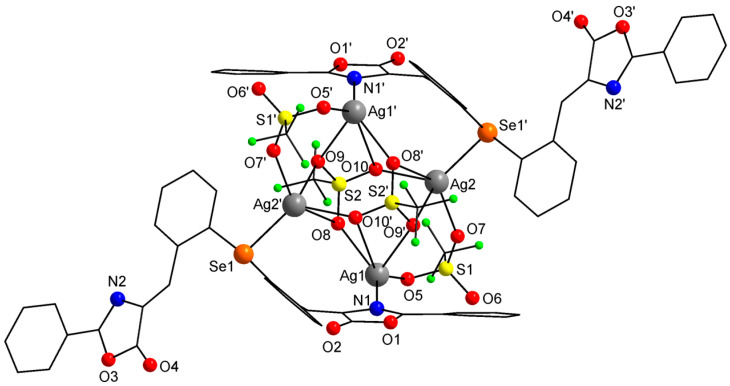
Tetranuclear structure in the crystal of **9**·4CHCl_3_ [symmetry equivalent position (2−x, 1−y, 1−z) is given by “prime”]. Hydrogen atoms and the molecules of CHCl_3_ were omitted for clarity.

Two dinuclear fragments corresponding to the asymmetric unit are connected to build a so-called dimer which exhibits a tetranuclear silver core [Ag_4_(OTf)_4_] formed by four metal atoms bridged by four triflate moieties. Moreover, both organoselenium ligands are bridging silver atoms from different asymmetric units using, in addition to a nitrogen atom, the selenium atom, Ag2−Se1’ 2.5691(1) Å (cf. Σr_vdW_(Ag,Se) 4.41 Å [47], Σr_cov_(Ag,Se) 2.65 Å [48]); thus, the diorganoselenium(II) ligand exhibits a 1κN:2κSe coordination pattern (Figure 6).

The silver atoms are penta-coordinated, and no Ag∙∙∙Ag metallophilic interactions were observed, the distances between the silver atoms [Ag1∙∙∙Ag2 3.959(1) Å and Ag2∙∙∙Ag1’ 4.345(1) Å, respectively] being longer than the Ag∙∙∙Ag distance (3.310 Å) observed in [Ag_2_(OTf)_2_(3,3‘-DCPA)] (DCPA = dicyanodiphenylacetylene) [49]. The NAg(1)O_4_ and SeAg(2)O_4_ cores around the silver atoms are distorted square-pyramidal as confirmed by τ_5_ values of 0.20 and 0.18, respectively [50]. Rather strong intermolecular C−H_solvent_···O (H36···O6 2.47 Å) interactions involving an oxygen atom from a triflate anion and a hydrogen atom from a crystallization chloroform molecule, as well as C−H_phenyl_···Cl (H32’’∙∙∙Cl6 2.80 Å, cf. Σr_vdW_(Cl,H) 3.02 Å [47]) involving an aromatic hydrogen from a phenyl ring and one of the chlorine atoms from the same solvent molecule, resulted in a polymeric chain association in the crystal of **9**·4CHCl_3_ (see Supplementary Material, Figure S9). Such parallel chains are intercalated, and weak interchain C−H_phenyl_···F contacts (H30a∙∙∙F2 2.63Å, cf. Σr_vdW_(F,H) 2.66 Å[47]), involving a hydrogen atom from the phenyl ring and a fluorine atom from a triflate fragment of a neighboring chain, resulted in a 2D supramolecular network (see Supplementary Material, Figure S10).

## 3. Materials and Methods

### 3.1. General Procedures

The starting materials, 2-(CHO)C_6_H_4_Br, 4-(CHO)C_6_H_4_Br, *n*-BuLi, *N*-benzoylglycine, sodium acetate, AgOTf, AgPF_6_ and ZnCl_2_, were commercially available and were used without additional purification. Se(detc)_2_ [51], 2-[(CH_2_O)_2_CH]C_6_H_4_Br, 4-[(CH_2_O)_2_CH]C_6_H_4_Br [26] and AuCl(tht) [52] were prepared following literature methods. The solvents used for the preparation of precursors **1**–**4** were dried following standard procedures and distilled under an argon atmosphere prior to use. Multinuclear NMR spectra were recorded in CDCl_3_ or acetone-*d*_6_, at room temperature, on Bruker AVANCE III (Bruker, Billerica, MA, USA) 400 and 600 MHz spectrometers operating at 400.13 MHz (^1^H), 100.62 MHz (^13^C), 376.49 MHz (^19^F), 76.31 MHz (^77^Se), and 600.13 MHz (^1^H), 150.90 MHz (^13^C), 564.69 MHz (^19^F), 242.94 (^31^P), 114.45 MHz (^77^Se), respectively. The ^1^H chemical shifts are reported in *δ* units (ppm) relative to the residual peak of the deuterated solvent (CHCl_3_, 7.26 ppm; acetone-*d*_5_, 2.05 ppm). The ^13^C chemical shifts are reported in *δ* units (ppm) relative to the peak of the deuterated solvent (CDCl_3_, 77.16 ppm; acetone-*d*_6_, 29.84 ppm) [53]. For the ^19^F{^1^H}, ^31^P{^1^H} and ^77^Se{^1^H} NMR spectra, the chemical shifts are reported in δ units (ppm) relative to CFCl_3_, H_3_PO_4_ 85% and Me_2_Se, respectively. The NMR data were processed using the MestReNova software (version 14) [54]. The ^1^H and ^13^C resonances were assigned using 2D NMR correlation experiments (COSY, HSQC and HMBC) according to the numbering scheme displayed in Scheme 3. ESI HRMS spectra were measured on a Thermo Scientific LTQ-OrbitrapXL instrument (Waltham, MA, USA) equipped with a standard ESI/APCI source. Infrared spectra were recorded on a JASCO FT/IR-615 instrument (Tokyo, Japan) in the range 400–4000 cm^−1^. The UV-Vis spectra were recorded in the range of 200‒900 nm in 10^−5^ M dichloromethane HPLC-grade solutions on a Cary 60 UV-Vis spectrophotometer (Agilent, Santa Clara, CA, USA). The photophysical investigation of the compounds was carried out at room temperature, for 10^−5^ M dichloromethane HPLC-grade solutions, using a Perkin Elmer LS 55 fluorimeter (Waltham, MA, USA). Molar conductivities of 10^−3^ M solutions in acetonitrile and acetone were measured with a TDS Meter CON 51 version 140 conductometer. Melting points were determined on an Electrothermal 9200 apparatus. Elemental analyses were carried out on a Thermo Flash EA-1112 analyzer (Thermo Scientific, Waltham, MA, USA).

### 3.2. Synthesis and Characterization

**[2-{(CH_2_O)_2_CH}C_6_H_4_]_2_Se (1)**

A solution of *n*-BuLi in hexane (11.3 mL, 1.6 M, 18.13 mmol, 20% excess) was added dropwise to a solution of 4-[(CH_2_O)_2_CH]C_6_H_4_Br (3.46 g, 15.10 mmol) in 60 mL anhydrous THF, under argon. After stirring for 1 h, Se(detc)_2_ (2.84 g, 7.56 mmol) was added, and the reaction mixture was stirred for 2 h. After removing the solvent in vacuum, 15 mL of water was added, and the compound was extracted with DCM and dried over anhydrous MgSO_4_. After filtration, the solvent was removed in vacuum, and the remaining solid was purified by washing it with hexane. Yield = 1.77 g (62%). **^1^H NMR** (600 MHz, CDCl_3_): δ 7.62 (d, ^3^*J*_HH_ = 7.6 Hz, 2H, H-6), 7.34–7.27 (m, 4H, H-3,5), 7.20 (t, ^3^*J*_HH_ = 7.7 Hz, 2H, H-4), 6.14 (s, 2H, H-7), 4.00–4.19 (m, 8H, H-8,9). **^77^Se{^1^H} NMR** (114 MHz, CDCl_3_): δ 321.

**[4-{(CH_2_O)_2_CH}C_6_H_4_]_2_Se (2)**

A solution of 4-[(CH_2_O)_2_CH]C_6_H_4_Br (2.61 g, 11.39 mmol) in 40 mL anhydrous THF was added dropwise to a solution of *n*-BuLi in hexane (8.54 mL, 1.6 M, 13.67 mmol, 20% excess) in 20 mL THF at −78 °C, under argon. After 1 h, Se(detc)_2_ (2.14 g, 5.70 mmol) was added, and the mixture was stirred for 3 h at −78 °C. The reaction mixture was allowed to warm to room temperature, and the solvent was removed in vacuum. Water (15 mL) was added to the residue, and the compound was extracted with DCM and dried over anhydrous MgSO_4_. After filtration, the solvent was removed in vacuum, and the title compound was isolated as a solid after being washed with hexane. Yield = 1.28 g (60%). **^1^H NMR** (600 MHz, CDCl_3_): δ 7.51 (d, ^3^*J*_HH_ = 8.3 Hz, 4H, H-2,6), 7.36 (d, ^3^*J*_HH_ = 8.2 Hz, 4H, H-3,5), 5.77 (s, 2H, H-7), 4.13–4.03 (m, 8H, H-8,9). **^13^C{^1^H} NMR** (151 MHz, CDCl_3_): δ 137.11 (C-4), 131.62 (C-2,6), 128.31 (C-3,5), 123.35 (C-1), 103.15 (C-7), 65.44 (C-8,9). **^77^Se{^1^H} NMR** (114 MHz, CDCl_3_) δ 414.

**[2-(O=CH)C_6_H_4_]_2_Se (3)**

To a solution of {2-[(CH_2_O)_2_CH]C_6_H_4_}_2_Se (1.77 g, 4.69 mmol) in 40 mL acetone, 0.5 mL HCl 37% was added. The reaction mixture was refluxed for 1 h at 100 °C. Then, 15 mL of water was added, and the compound was extracted with DCM and dried over anhydrous MgSO_4_. After filtration, the solvent was removed in vacuum, and the title compound was washed with hexane. Yield = 1.24 g (91%). **^1^H NMR** (400 MHz, CDCl_3_): δ 10.28 (s, 2H, H-7), 7.98 (d, ^3^*J*_HH_ = 7.78 Hz, 4H, H-6), 7.48–7.53 (m, 4H, H-4,5).

**[4-(O=CH)C_6_H_4_]_2_Se (4)**

To a solution of {4-[(CH_2_O)_2_CH]C_6_H_4_}_2_Se (1.28 g, 3.39 mmol) in 40 mL acetone, 0.5 mL HCl 37% was added. The reaction mixture was refluxed for 1 h at 100 °C; then, it was allowed to reach room temperature, and 15 mL of water was added. The reaction mixture was extracted with DCM and filtered, and the clear solution was dried over anhydrous MgSO_4_. After filtration, the solvent was removed in vacuum, and the title compound was isolated by column purification with 4:1 (*v*:*v*) DCM:EtOAc. Yield = 0.43 g (44%). M.p. 105 °C. **Elemental analysis** calc. for C_14_H_10_O_2_Se (289.98): C, 58.15; H, 3.49; found: C, 58.96; H, 3.97. **^1^H NMR** (400 MHz, CDCl_3_): δ 9.98 (s, 2H, H-7), 7.79 (d, ^3^*J*_HH_ = 8.1 Hz, 4H, H-3,5), 7.62 (d, ^3^*J*_HH_ = 8.1 Hz, 4H, H-2,6). **^13^C{^1^H} NMR** (101 MHz, CDCl_3_): δ 191.45 (C-7), 138.89 (C-1), 135.61 (C-4), 133.13 (C-2,6), 130.58 (C-3,5). **^77^Se{^1^H} NMR** (76 MHz, CDCl_3_): δ 444. **IR** (KBr pellet, ν, cm^−1^): 1710 (s) [ν(C=O)], 1688 (s), 1665 (m), 1618 (w), 1585 (s), 1561 (m), 1485 (w), 1409 (w), 1381 (w), 1301 (w), 1280 (w), 1215 (m), 1200 (m), 1169 (m), 1095 (w), 1058 (m), 1011 (m), 976 (w), 835 (s), 817 (s), 678 (w). **HRMS** (APCI+, MeCN), *m*/*z* (%): 290.99249 (100), [M+H]^+^ calcd. for C_14_H_11_O_2_Se: *m*/*z* = 290.99188; 211.07536 (10), [C_14_H_11_O_2_]^+^ calcd. for C_14_H_11_O_2_: *m*/*z* = 211.07536; 184.95055 (10), [C_7_H_5_OSe]^+^ calcd. for C_7_H_5_OSe: *m*/*z* = 184.95001; 156.95555 (10), [C_6_H_5_Se]^+^ calcd. for C_6_H_5_Se: *m*/*z* = 156.95510.

**[(*Z*)-2′-{2-C_6_H_5_-(4*H*)-oxazol-5-one}CHC_6_H_4_]_2_Se (5)**

A reaction mixture of [2-(O=CH)C_6_H_4_]_2_Se (**3**) (0.784 g, 2.71 mmol), C_6_H_5_CH(O)NHCH_2_COOH (0.971 g, 5.42 mmol), sodium acetate (0.890 g, 10.84 mmol) and 2 mL acetic anhydride was heated for 2 h, at 100 °C. Then, EtOH was added to the reaction mixture at room temperature, when the title compound precipitated and was isolated by filtration as an intense yellow solid that was washed several times with EtOH. Yield = 1.175 g (75%). M.p. 247 °C. **Elemental analysis** calc. for C_32_H_20_N_2_O_4_Se (576.06): C, 66.79; H, 3.50; N, 4.87; found: C, 66.43; H, 3.48; N, 4.67 calcd. **^1^H NMR** (600 MHz, CDCl_3_): δ 8.81 (d, ^3^*J*_HH_ = 8.0 Hz, 2H, H-3), 8.16 (d, ^3^*J*_HH_ = 7.0 Hz, 4H, H-12,16), 7.85 (s, 2H, H-7), 7.62 (t, ^3^*J*_HH_ = 7.5 Hz, 2H, H-14), 7.55–7.50 (m, 6H, H-6, H-13,15), 7.45 (t, ^3^*J*_HH_ = 8.3 Hz, 2H, H-4), 7.29 (t, ^3^*J*_HH_ = 7.6 Hz, 2H, H-5). **^13^C{^1^H} NMR** (151 MHz, CDCl_3_): δ 167.31 (C-8), 164.42 (C-10), 136.02 (C-1), 135.44 (C-2), 135.41 (C-6), 134.51 (C-9), 133.67 (C-14), 133.29 (C-3), 131.58 (C-5), 129.96 (C-7), 129.11 (C-13,15) 128.77 (C-4), 128.65 (C-12,16), 125.63 (C-11). **^77^Se{^1^H} NMR** (114 MHz, CDCl_3_): δ 344. **IR** (KBr pellet, ν, cm^−1^): 1792 (s)/1770 (m) [ν(C=O)], 1651 (m) [ν(C=N)], 1576 (m), 1556 (m), 1491 (w), 1451 (m), 1433 (m), 1407 (m), 1360 (w), 1327 (m), 1289 (m)/1225 (w) [ν(C−O)], 1165 (s), 1109 (w), 1069 (w), 1025 (w), 979 (w), 919 (w), 889 (w), 866 (m), 768 (m), 698 (s), 634 (w). **HRMS** (APCI+, MeCN), *m*/*z* (%): 577.06329 (100), [M+H]^+^ calcd. for C_32_H_21_N_2_O_4_Se: *m*/*z* = 577.06611; 327.98578 (15), [C_16_H_10_NO_2_Se]^+^ calcd. for C_16_H_10_NO_2_Se: *m*/*z* = 327.98713.

**[(*Z*)-4′-{2-C_6_H_5_-(4*H*)-oxazol-5-one}CHC_6_H_4_]_2_Se (6)**

Using a similar procedure as described above for compound **5**, the related derivative **6** was prepared from [4-(OCH_2_)C_6_H_4_]_2_Se (**4**) (0.431 g, 1.49 mmol), C_6_H_5_CH(O)NHCH_2_COOH (0.534 g, 2.98 mmol), sodium acetate (0.489 g, 5.96 mmol) and 2 mL acetic anhydride. It was isolated as an intense yellow solid. Yield = 0.76 g (88%). M.p. 160 °C. **Elemental analysis** calc. for C_32_H_20_N_2_O_4_Se (576.06): C, 66.79; H, 3.50; N, 4.87; found: C, 66.41; H, 3.77; N, 4.66. **^1^H NMR** (400 MHz, CDCl_3_): δ 8.17 (d, 4H, H-12,16), 8.15 (d, 4H, H-3,5), 7.63 (t, 2H, H-14), 7.60 (d, 4H, H-2,6), 7.53 (t, 4H, H-13,15), 7.22 (s, 2H, H-7). **^13^C{^1^H} NMR** (101 MHz, CDCl_3_): δ 167.62 (C-8), 163.87 (C-10), 135.43 (C-4), 133.75 (C-9), 133.67 (C-14), 133.24 (C-3,5), 133.17 (C-2,6), 132.96 (C-1), 130.72 (C-7), 129.14 (C-13,15), 128.57 (C-12,16), 125.58 (C-11). **^77^Se{^1^H} NMR** (76 MHz, CDCl_3_): δ 438. **IR** (KBr pellet, ν, cm^−1^): 1795 (s)/1768 (w) [ν(C=O)], 1653 (m) [ν(C=N)], 1577 (m), 1558 (m), 1487 (w), 1449 (w), 1403 (w), 1368 (w), 1327 (w), 1295 (w)/1238 (w) [ν(C−O)], 1164 (m), 1063 (w), 1011 (w), 982 (w), 887 (w), 866 (w), 811 (w), 777 (w), 697 (m). **HRMS** (APCI+, MeCN), *m*/*z* (%): 577.06671 (100), [M+H]^+^ calcd. for C_32_H_21_N_2_O_4_Se: *m*/*z* = 577.06611.

**[Ag(OTf){[(*Z*)-2′-{2-C_6_H_5_-(4*H*)-oxazol-5-one}CHC_6_H_4_]_2_Se}] (7)**

Silver triflate (0.045 g, 0.17 mmol) was added to a solution of compound **5** (0.1 g, 0.17 mmol) in 20 mL acetone. After 30 min of stirring, in the absence of light, the solvent was partially removed under vacuum, and the title compound was precipitated with Et_2_O. After the product was washed with Et_2_O (3 × 10 mL), an intense yellow solid was isolated. Yield = 0.14 g (97%). M.p. 108 °C (desc.). **Elemental analysis** calc. for C_33_H_20_AgF_3_N_2_O_7_SSe (831.92) C, 47.62; H, 2.42; N, 3.37; S, 3.85; found: C, 47.58; H, 2.55; N, 3.48; S, 4.10. **^1^H NMR** (600 MHz, acetone-*d*_6_): δ 8.90 (dd, ^3^*J*_HH_ = 8.0 Hz, ^4^*J*_HH_ = 1.5 Hz, 2H, H-3), 8.18 (m, 4H, H-12,16), 7.79 (s, 2H, H-7), 7.74 (tt, ^3^*J*_HH_ = 7.5 Hz, ^4^*J*_HH_ = 1.3 Hz, 2H, H-14), 7.64 (t, ^3^*J*_HH_ = 7.7 Hz, 4H, H-13,15), 7.60 (dd, ^3^*J*_HH_ = 7.8 Hz, ^4^J_HH_ = 1.2 Hz, 2H, H-6), 7.55 (t, ^3^*J*_HH_ = 8.3 Hz, 2H, H-4), 7.42 (td, ^3^*J*_HH_ = 7.6 Hz, ^4^J_HH_ = 1.6 Hz, 2H, H-5). **^13^C{^1^H} NMR** (151 MHz, CDCl_3_): δ 167.44 (C-8), 165.51 (C-10), 136.18 (C-6,9), 136.06 (C-1,2), 134.68 (C-14), 133.94 (C-3), 132.52 (C-5), 130.10 (C-13,15), 129.75 (C-4), 129.19 (C-12,16), 129.07 (C-7), 126.44 (C-11), 120.69 (q, ^1^*J*_CF_ = 123.5 Hz, CF_3_). **^19^F{^1^H} NMR** (565 MHz, acetone-*d*_6_): δ −78.8. **IR** (KBr pellet, ν, cm^−1^): 1792 (m)/1771 (w) [ν(C=O)], 1651 (m) [ν(C=N)], 1576 (s), 1558 (s), 1491 (w), 1451 (m), 1409 (m), 1327 (m), 1289 (m)/1259 (m) [ν(C−O)], 1227 (w), 1165 (m), 1109 (w), 1037 (m), 980 (w), 920 (w), 889 (w), 866 (m), 768 (m), 698 (m), 649 (m). **HRMS** (ESI+, MeCN), *m*/*z* (%): 682.96344 (100), [M−OTf]^+^ calcd. for C_32_H_20_AgN_2_O_4_Se: *m*/*z* = 682.96337. **Λ_M_** (MeCN): 152 Ω^−1^·cm^2^·mol^−1^. **Λ_M_** (acetone): 135 Ω^−1^·cm^2^·mol^−1^.

**[Ag(OTf){[(Z)-4′-{2-C_6_H_5_-(4*H*)-oxazol-5-one}CHC_6_H_4_]_2_Se}] (8)**

As described above for **7**, compound **8** was prepared from silver triflate (0.046 g, 0.18 mmol) and a solution of compound **6** (0.103 g, 0.18 mmol) in 20 mL acetone. Yield = 0.102 g (69%). M.p. 233 °C (desc.). **Elemental analysis** calc. for C_33_H_20_AgF_3_N_2_O_7_SSe (831.92) C, 47.62; H, 2.42; N, 3.37; S, 3.85; found: C, 48.25; H, 2.49; N, 3.75; S, 4.12. **^1^H NMR** (600 MHz, acetone-*d*_6_): δ 8.38 (d, ^3^*J*_HH_ = 8.3 Hz, 4H, H-3,5), 8.23 (d, ^3^*J*_HH_ = 8.2 Hz, 4H, H-12,16), 7.74 (t, ^3^*J*_HH_ = 7.5 Hz, 2H, H-14), 7.70 (d, ^3^*J*_HH_ = 8.4 Hz, 4H, H-2,6), 7.66 (t, ^3^*J*_HH_ = 7.7 Hz, 4H, H-13,15), 7.32 (s, 2H, H-7). **^13^C{^1^H} NMR** (151 MHz, acetone-*d*_6_): δ 167.61 (C-8), 164.74 (C-10), 135.63 (C-4), 134.75 (C-9), 134.54 (C-14), 134.16 (C-1,3,5), 133.85 (C-2,6), 130.55 (C-7), 130.10 (C-13,15), 129.10 (C-12,16), 126.51 (C-11), 121.98 (q, ^1^*J*_CF_ = 122.2 Hz, CF_3_). **^19^F{^1^H} NMR** (565 MHz, acetone-*d*_6_): δ −78.89. **IR** (KBr pellet, ν, cm^−1^): 1796 (m) [ν(C=O)], 1653 (m) [ν(C=N)], 1578 (s), 1488 (w), 1449 (w), 1406 (m), 1294 (m)/1259 (s) [ν(C−O)], 1231 (m), 1176 (m), 1037 (m), 980 (w), 924 (w), 862 (w), 817 (w), 767 (w), 697 (w), 648 (m). **HRMS** (ESI+, MeCN), *m*/*z* (%): 147.93069 (15), [Ag+MeCN]^+^ calcd. for C_2_H_3_AgN: *m*/*z* =147.93109; 682.96094 (5), [M−OTf]^+^ calcd. for C_32_H_20_AgN_2_O_4_Se: *m*/*z* = 682.96337. **Λ_M_** (MeCN): 165 Ω^−1^·cm^2^·mol^−1^. **Λ_M_** (acetone): 141 Ω^−1^·cm^2^·mol^−1^.

**[{Ag(OTf)}_2_{[(*Z*)-2′-{2-C_6_H_5_-(4*H*)-oxazol-5-one}CHC_6_H_4_]_2_Se}] (9)**

Silver triflate (0.053 g, 0.21 mmol) was added to a solution of compound **5** (0.06 g, 0.10 mmol) in 20 mL acetone. The reaction mixture was stirred for 30 min in the absence of light, and the solvent was partially removed under vacuum. The compound was precipitated with Et_2_O, washed with Et_2_O (3 × 10 mL) and dried under vacuum. Yield = 0.11 g (97%). M.p. 89 °C (desc.). **^1^H NMR** (600 MHz, acetone-*d*_6_): δ 8.81 (d, ^3^*J*_HH_ = 7.9 Hz, 2H, H-3), 8.18 (d, ^3^*J*_HH_ = 6.9 Hz, 4H, H-12,16), 7.77–7.72 (m, 4H, H-7, H-14), 7.71 (d, ^3^*J*_HH_ = 7.7 Hz, 2H, H-6), 7.64 (t, ^3^*J*_HH_ = 7.8 Hz, 4H, H-13,15), 7.57 (t, ^3^*J*_HH_ = 7.6 Hz, 2H, H-4), 7.44 (t, ^3^*J*_HH_ = 7.6 Hz, 2H, H-5). **^13^C{^1^H} NMR** (151 MHz, acetone-*d*_6_): δ 167.46 (C-8), 165.51 (C-10), 136.50 (C-6), 136.07 (C-4), 135.48 (C-1), 135.81 (C-2), 134.82 (C-14), 133.79 (C-9), 133.76 (C-3), 132.54 (C-5), 130.13 (C-13,15), 129.23 (C-12,16), 129.07 (C-7), 126.30 (C-11), 121.98 (q, ^1^*J*_CF_ = 320.5 Hz, CF_3_). **^19^F{^1^H} NMR** (565 MHz, acetone-*d*_6_): δ −78.61. **IR** (KBr pellet, ν, cm^−1^): 1793 (m)/1764 (m) [ν(C=O)], 1650 (m) [ν(C=N)], 1591 (w), 1554 (m), 1488 (w), 1454 (w), 1358 (w), 1329 (m), 1296 (s)/1255 (s) [ν(C−O)], 1224 (m), 1162 (s), 1105 (w), 1033 (m), 981 (w), 913 (w), 863 (m), 764 (m), 693 (m), 646 (m), 558 (w), 518 (w), 470 (w). **HRMS** (ESI+, MeCN), *m*/*z* (%): 682.96344 (100) [M−Ag(OTf)_2_]^+^ calcd. for: C_32_H_20_AgN_2_O_4_Se *m*/*z* = 682.96337; *m*/*z* (%): 791.10040 (5) [M−(OTf)_2_]^+^ calcd. for: C_32_H_20_Ag_2_N_2_O_4_Se *m*/*z* = 791.86813. **Λ_M_** (MeCN): 250 Ω^−1^·cm^2^·mol^−1^. **Λ_M_** (acetone): 179 Ω^−1^·cm^2^·mol^−1^.

**[{Ag(OTf)}_2_{[(*Z*)-4′-{2-C_6_H_5_-(4*H*)-oxazol-5-one}CHC_6_H_4_]_2_Se}] (10)**

As described above for **9**, compound **10** was prepared from silver triflate (0.053 g, 0.20 mmol) and a solution of compound **6** (0.06 g, 0.10 mmol) in acetone. Yield = 0.10 g (88%). M.p. 148.2 °C (desc.). **^1^H NMR** (400 MHz, acetone-*d*_6_): δ 8.39 (d, ^3^*J*_HH_ = 8.4 Hz, 4H, H-3,5), 8.20 (d, ^3^*J*_HH_ = 8.4 Hz, 4H, H-12,16), 7.83 (d, ^3^*J*_HH_ = 8.4 Hz, 4H, H-2,6), 7.73 (t, ^3^*J*_HH_ = 7.4 Hz, 2H, H-14), 7.64 (t, ^3^*J*_HH_ = 7.6 Hz, 4H, H-13,15), 7.31 (s, 2H, H-7). **^13^C{^1^H} NMR** (101 MHz, acetone-*d*_6_): δ 167.49 (C-8), 165.02 (C-10), 134.64 (C-14), 134.33 (C-4), 134.29 (C-9), 134.24 (C-1,3,5), 133.76 (C-2,6), 130.18 (C-7), 130.10 (C-13,15), 129.13 (C-12,16), 126.76 (C-11), 121.98 (q, ^1^*J*_CF_ = 321.2 Hz, CF_3_). **^19^F{^1^H} NMR** (376 MHz, acetone-*d*_6_): δ −78.61. **Λ_M_** (MeCN): 230 Ω^−1^·cm^2^·mol^−1^. **IR** (KBr pellet, ν, cm^−1^): 1795 (m) [ν(C=O)], 1653 (m) [ν(C=N)], 1578 (s), 1488 (w), 1444 (m), 1406 (m), 1370 (w), 1329 (m), 1292 (s)/1257 (s) [ν(C−O)], 1228 (m), 1168 (m), 1041 (m), 983 (w), 928 (w), 894 (w), 864 (w), 811 (w), 772 (w), 693 (w), 645 (m), 580 (w), 520 (w), 457 (w). **HRMS** (ESI+, MeCN), *m*/*z* (%): 791.14984 (5) [M−(OTf)_2_]^+^ calcd. for: C_32_H_20_Ag_2_N_2_O_4_Se *m*/*z* = 791.86813. **Λ_M_** (acetone): 200 Ω^−1^·cm^2^·mol^−1^.

**[Ag{[(*Z*)-2′-{2-C_6_H_5_-(4*H*)-oxazol-5-one}CHC_6_H_4_]_2_Se}][PF_6_] (11)**

Silver hexafluorophosphate (0.028 g, 0.11 mmol) was added to a solution of compound **5** (0.064 g, 0.11 mmol) in 20 mL CHCl_3_. After 30 min of stirring, the solvent was partially removed under vacuum. An intense yellow solid was isolated after the precipitation with Et_2_O and was purified by washing it with Et_2_O (3 × 5 mL). Yield = 0.09 g (98%). M.p. 120 °C (desc.). **Elemental analysis** calc. for C_32_H_20_AgF_6_N_2_O_4_PSe (827.93) C, 46.40; H, 2.43; N, 3.38; found: C, 46.45; H, 2.56; N,3.43. **^1^H NMR** (600 MHz, acetone-*d*_6_): δ 8.91 (d, ^3^*J*_HH_ = 8.0 Hz, 2H, H-3), 8.19 (d, ^3^*J*_HH_ = 7.0 Hz, 4H, H-12,16), 7.80 (s, 2H, H-7), 7.74 (t, ^3^*J*_HH_ = 7.5 Hz, 2H, H-14), 7.66 (t, 4H, H-13,15), 7.62 (d, 2H, H-6), 7.56 (t, ^3^*J*_HH_ = 8.3 Hz, 2H, H-4), 7.42 (t, ^3^*J*_HH_ = 7.6 Hz, 2H, H-5). **^13^C{^1^H} NMR** (151 MHz, acetone-*d*_6_): δ 167.53 (C-8), 165.53 (C-10), 136.19 (C-6,9), 136.09 (C-2), 135.25 (C-1), 134.69 (C-14), 133.95 (C-3), 132.52 (C-5), 130.11 (C-13,15), 129.75 (C-4), 129.20 (C-12,16), 129.11 (C-7), 126.46 (C-11). **^19^F{^1^H} NMR** (376 MHz, acetone-*d*_6_): δ −72.70 (d, ^1^J_FP_ = 708 Hz). **^31^P{^1^H} NMR** (243 MHz, acetone-*d*_6_): δ −144.27 (hept, ^1^*J*_PF_ = 708 Hz). **IR** (KBr pellet, ν, cm^−1^): 1792 (s)/1771 (m) [ν(C=O)], 1652 (m) [ν(C=N)], 1575 (m), 1556 (m), 1491 (w), 1451 (m), 1417 (w), 1360 (w), 1327 (m), 1302 (w), 1289 (w)/1225 (w) [ν(C−O)], 1165 (s), 1109 (m), 1069 (w), 1024 (w), 980 (w), 917 (w), 889 (m), 866 (s), 831 (s), 768 (m), 698 (m), 667 (w). **HRMS** (APCI+, MeCN), *m*/*z* (%): 577.06433 (100), [M−AgPF_6_+H]^+^ calcd. for C_32_H_21_N_2_O_4_Se: *m*/*z* = 577.06611; 327.98621 (90), [C_16_H_10_NO_2_Se]^+^ calcd. for C_16_H_10_NO_2_Se: *m*/*z* = 327.98713; 283.99655 (35) [C_16_H_10_NO_2_Se-COO]^+^ calcd. for C_15_H_10_NSe: *m*/*z* = 283.99730. **HRMS** (ESI+, MeCN), *m*/*z* (%): 682.96246 (10), [M−PF_6_]^+^ calcd. for: C_32_H_20_AgN_2_O_4_Se *m*/*z* = 682.96337. **Λ_M_** (MeCN): 124 Ω^−1^·cm^2^·mol^−1^. **Λ_M_** (acetone): 113 Ω^−1^·cm^2^·mol^−1^.

**[Ag{[(*Z*)-4′-{2-C_6_H_5_-(4*H*)-oxazol-5-one}CHC_6_H_4_]_2_Se}][PF_6_] (12)**

As described above for **11**, compound **12** was prepared from silver hexafluorophosphate (0.036 g, 0.14 mmol) and a solution of compound **6** (0.082 g, 0.14 mmol) in CHCl_3_. Yield = 0.12 g (99%). M.p. 239 °C (desc.). **Elemental analysis** calc. for C_32_H_20_AgF_6_N_2_O_4_PSe (827.93) C, 46.40; H, 2.43; N, 3.38; found: C, 46.41; H, 2.35; N, 3.15. **^1^H NMR** (600 MHz, acetone-*d*_6_): δ 8.38 (d, ^3^*J*_HH_ = 8.3 Hz, 4H, H-3,5), 8.23 (d, ^3^*J*_HH_ = 7.4 Hz, 4H, H-12,16), 7.74 (t, ^3^*J*_HH_ = 7.5 Hz, 2H, H-14), 7.70 (d, ^3^*J*_HH_ = 8.4 Hz, 4H, H-2,6), 7.66 (t, ^3^*J*_HH_ = 7.7 Hz, 4H, H-13,15), 7.22 (s, 2H, H-7). **^13^C{^1^H} NMR** (151 MHz, acetone-*d*_6_): δ 167.63 (C-8), 164.73 (C-10), 135.72 (C-4), 134.74 (C-9), 134.53 (C-14), 134.16 (C-3,5), 134.12 (C-1), 133.83 (C-2,6), 130.57 (C-7), 130.10 (C-13,15), 129.10 (C-12,16), 126.53 (C-11). **^19^F{^1^H} NMR** (565 MHz, acetone-*d*_6_): δ −72.70 (d, ^1^J_FP_ = 708 Hz). **^31^P{^1^H} NMR** (243 MHz, acetone-*d*_6_): δ −144.29 (hept, ^1^J_PF_ = 708 Hz. **IR** (KBr pellet, ν, cm^−1^): 1796 (s)/1769 (w) [ν(C=O)], 1653 (m) [ν(C=N)], 1578 (s), 1558 (m), 1487 (w), 1450 (m), 1406 (m), 1367 (w), 1327 (m), 1297 (m) [ν(C−O)], 1165 (m), 1064 (w), 1012 (w), 983 (w), 888 (m), 863 (s), 831 (s), 777 (w), 699 (m). **HRMS** (APCI+, MeCN), *m*/*z* (%): 577.06329 (100), [M−AgPF_6_+H]^+^ calcd. for C_32_H_21_N_2_O_4_Se: *m*/*z* = 577.06611. **HRMS** (ESI+, MeCN), *m*/*z* (%): 682.96124 (90), [M−PF_6_]^+^ calcd. for C_32_H_20_AgN_2_O_4_Se: *m*/*z* = 682.96337. **Λ_M_** (MeCN): 150 Ω^−1^·cm^2^·mol^−1^. **Λ_M_** (acetone): 129 Ω^−1^·cm^2^·mol^−1^.

**[AuCl{[(*Z*)-2′-{2-C_6_H_5_-(4*H*)-oxazol-5-one}CHC_6_H_4_]_2_Se}] (13)**

AuCl(tht) (0.056 g, 0.17 mmol) was added to a solution of compound **5** (0.1 g, 0.17 mmol), in 15 mL CHCl_3_. After 30 min of stirring, the solvent was removed under vacuum, and the remaining solid was purified by washing it with hexane (3 × 10 mL). Yield = 0.14 g (96%). M.p. 166 °C. **Elemental analysis** calc. for C_32_H_20_AuClN_2_O_4_Se (807.99) C, 47.57; H, 2.50; N, 3.47; found: C, 47.44; H,3.33; N, 3.59. **^1^H NMR** (600 MHz, CDCl_3_): δ 8.81 (d, ^3^*J*_HH_ = 8.0 Hz, 2H, H-3), 8.16 (d, ^3^*J*_HH_ = 7.0 Hz, 4H, H-12,16), 7.85 (s, 2H, H-7), 7.61 (t, ^3^*J*_HH_ = 7.5 Hz, 2H, H-14), 7.55–7.50 (m, 6H, H-6,13,15), 7.45 (t, ^3^*J*_HH_ = 8.3 Hz, 2H, H-4), 7.29 (t, ^3^*J*_HH_ = 7.6 Hz, 2H, H-5). **^13^C{^1^H} NMR** (151 MHz, CDCl_3_): δ 167.30 (C-8), 164.43 (C-10), 136.01 (C-1), 135.45 (C-2), 135.41 (C-6), 134.52(C-9), 133.67 (C-14), 133.29 (C-3), 131.57 (C-5), 129.96 (C-7), 129.11 (C-13), 128.77 (C-4), 128.65 (C-12), 125.65 (C-11). **^77^Se{^1^H} NMR** (115 MHz, CDCl_3_): δ 344. **IR** (KBr pellet, ν, cm^−1^): 1792 (s)/1771 (m) [ν(C=O)], 1651 (m) [ν(C=N)], 1576 (m), 1556 (m), 1492 (w), 1451 (m), 1433 (m), 1360 (w), 1327 (m), 1303 (m), 1290 (m)/1225 (m) [ν(C−O)], 1165 (s), 1109 (w), 1070 (w), 1026 (w), 980 (w), 920 (w), 889 (w), 866 (m), 768 (m), 698 (s), 634 (w). **HRMS** (ESI+, MeCN), *m*/*z* (%): 773.02191 (100), [M−Cl]^+^ calcd. for C_32_H_20_AuN_2_O_4_Se: *m*/*z* = 773.02483. **Λ_M_** (MeCN): 19 Ω^−1^·cm^2^·mol^−1^.

**[AuCl{[(*Z*)-4′-{2-C_6_H_5_-(4*H*)-oxazol-5-one}CHC_6_H_4_]_2_Se}] (14)**

As described above for **13**, compound **14** was prepared from AuCl(tht) (0.056 g, 0.17 mmol) and a CHCl_3_ solution of compound **6** (0.1 g, 0.17 mmol). Yield = 0.14 g (99%). M.p. 245 °C. **Elemental analysis** calc. for C_32_H_20_AuClN_2_O_4_Se (807.99) C, 47.57; H, 2.50; N, 3.47; found C, 47.10; H, 2.47; N, 3.41. **^1^H NMR** (600 MHz, CDCl_3_): δ 8.17 (d, ^3^*J*_HH_ = 7.44 Hz, 4H, H-3,5) 8.14 (d, ^3^*J*_HH_ = 8.27 Hz, 4H, H-12,16), 7.54–7.64 (m, 6H, H14, H-2,6), 7.53 (t, ^3^*J*_HH_ = 8.11 Hz, 4H, H-13,15), 7.22 (s, 2H, H-7). **^13^C{^1^H} NMR** (151 MHz, CDCl_3_): δ 167.60 (C-8), 163.93 (C-10), 135.31 (C-4), 133.81 (C-9), 133.69 (C-14), 133.20 (C-3,5), 133.26 (C-2,6), 132.28 (C-1), 130.66 (C-7), 129.15 (C-13,15), 128.58 (C-12,16), 125.58 (C-11). **^77^Se{^1^H} NMR** (115 MHz, CDCl_3_): δ 437. **IR** (KBr pellet, ν, cm^−1^): 1795 (s)/1768 (m) [ν(C=O)], 1652 (m) [ν(C=N)], 1577 (s), 1556 (m), 1486 (w), 1450 (m), 1405 (m), 1365 (w), 1327 (m), 1296 (m)/1267 (w) [ν(C−O)], 1163 (m), 1063 (w), 981 (w), 924 (w), 887 (w), 866 (m), 811 (m), 777 (w), 697 (m). **HRMS** (APCI+ MeCN), *m*/*z* (%): 577.06354 (50), [M−AuCl+H]^+^ calcd. for C_31_H_21_N_2_O_4_Se: *m*/*z* = 573.06880. **HRMS** (ESI+ MeCN), *m*/*z* (%): 1004.95911 (5), [M+Au]^+^ calcd. for C_32_H_20_Au_2_ClN_2_O_4_Se: *m*/*z* = 1004.96024. **Λ_M_** (MeCN): 14 Ω^−1^·cm^2^·mol^−1^.

**[** **ZnCl_2_{[(*Z*)-2′-{2-C_6_H_5_-(4*H*)-oxazol-5-one}CHC_6_H_4_]_2_Se}] (15)**

ZnCl_2_ (0.019 g, 0.14 mmol) was added to a solution of compound **5** (0.08 g, 0.14 mmol) in 20 mL CHCl_3_ and 5 mL absolute EtOH. After 30 min of stirring, the solvent was removed under vacuum, and the remaining solid was washed with hexane (3 × 10 mL). Yield = 0.95 g (97%). M.p. 220 °C **Elemental analysis** calc. for C_32_H_20_Cl_2_N_2_O_4_SeZn (709.93) C, 53.00; H, 2.83; N, 3.94; found: C, 53.71; H, 2.88; N, 3.84. **^1^H NMR** (400 MHz, CDCl_3_): δ 8.84 (d, ^3^*J*_HH_ = 7.9 Hz, 2H, H-3), 8.19 (d, ^3^*J*_HH_ = 7.0 Hz, 4H, H-12,16), 7.89 (s, 2H, H-7), 7.62 (t, ^3^*J*_HH_ = 7.7 Hz, 2H, H-14), 7.53 (m, 6H, H-6,13,15), 7.45 (t, ^3^*J*_HH_ = 7.7 Hz, 2H, H-4), 7.29 (t, ^3^*J*_HH_ = 7.7 Hz, 2H, H-5). **^13^C{^1^H} NMR** (101 MHz, CDCl_3_): δ 167.32 (C-8), 164.42 (C-10), 136.02 (C-2), 135.44 (C-1), 135.42 (C-6), 134.51 (C-9), 133.68 (C-14), 133.29 (C-3), 131.29 (C-5), 129.11 (C-13,15), 128.78 (C-4), 128.65 (C-12,16), 129.97 (C-7), 125.63 (C-11). **IR** (KBr pellet, ν, cm^−1^): 1797 (s)/1770 (m) [ν(C=O)], 1650 (m) [ν(C=N)], 1581 (w), 1554 (m), 1491 (w), 1450 (m), 1361 (w), 1325 (m), 1293 (m)/1224 (w) [ν(C−O)], 1166 (m), 1101 (w), 1070 (w), 1025 (w), 983 (w), 888 (w), 864 (m), 767 (m), 699 (s). **HRMS** (APCI+, MeCN), *m*/*z* (%): 577.06415 (100), [M−ZnCl_2_+H]^+^ calcd. for C_32_H_21_N_2_O_4_Se: *m*/*z* = 577.06611; 327.98615 (100), [C_16_H_10_N_1_O_2_Se]^+^ calcd. for C_16_H_10_N_1_O_2_Se: *m*/*z* = 327.98713. **Λ_M_** (MeCN): 65 Ω^−1^·cm^2^·mol^−1^.

**[ZnCl_2_{[(*Z*)-4′-{2-C_6_H_5_-(4*H*)-oxazol-5-one}CHC_6_H_4_]_2_Se}] (16)**

As described above for **15**, compound **16** was prepared from ZnCl_2_ (0.019 g, 0.14 mmol) and a solution of compound **6** (0.08 g, 0.14 mmol) in 20 mL CHCl_3_ and 5 mL absolute EtOH. Yield = 0.97 g (99%). M.p. 241 °C **Elemental analysis** calc. for C_32_H_20_Cl_2_N_2_O_4_SeZn (709.93) C, 53.00; H, 2.83; N, 3.94; found: C, 53.46; H, 2.95; N, 3.99. **^1^H NMR** (600 MHz, CDCl_3_): δ 8.18 (d, ^3^*J*_HH_ = 8.1 Hz, 4H, H-3,5), 8.15 (d, ^3^*J*_HH_ = 8.4 Hz, 4H, H-12,16), 7.62 (t, ^3^*J*_HH_ = 7.4 Hz, 2H, H-14), 7.59 (d, ^3^*J*_HH_ = 8.4 Hz, 4H, H-2,6), 7.53 (t, ^3^*J*_HH_ = 7.8 Hz, 4H, H-13,15), 7.22 (s, 2H, H-7). **^13^C{^1^H} NMR** (151 MHz, CDCl_3_): δ 167.63 (C-8), 163.91 (C-10), 135.45 (C-4), 133.67 (C-9), 133.58 (C-14), 133.25 (C-3,5), 133.19 (C-2,6), 132.99 (C-1), 130.73 (C-7), 129.15 (C-13,15), 128.59 (C-12,16), 125.61 (C-11). **^77^Se{^1^H} NMR** (115 MHz, CDCl_3_): δ 437. **IR** (KBr pellet, ν, cm^−1^): 1795 (s)/1768 (m) [ν(C=O)], 1652 (m) [ν(C=N)], 1578 (m), 1557 (m), 1487 (w), 1451 (w), 1404 (w), 1365 (w), 1327 (m), 1297 (w)/1236 (w) [ν(C−O)], 1162 (m), 1064 (w), 1011 (w), 981 (w), 888 (w), 867 (w), 818 (w), 779 (w), 698 (m). **HRMS** (APCI+, MeCN), *m*/*z* (%): 577.06354 (100), [M−ZnCl_2_+H]^+^ calcd. for C_32_H_21_N_2_O_4_Se: *m*/*z* = 577.06611. **Λ_M_** (MeCN): 30 Ω^−1^·cm^2^·mol^−1^.

### 3.3. Crystal Structure Determination

Vapor diffusion of *n*-hexane into a DCM solution of compounds **4** and **5** or a CHCl_3_ solution of **9** allowed the formation of crystals suitable for X-ray diffraction. Table S1 provided in the Supplementary Material illustrates the details of the crystal structure determination and refinement. The crystals were enclosed on MiTeGen microMounts cryoloops, and the data were collected on a Bruker D8 VENTURE diffractometer using Mo-Kα radiation (λ = 0.71073 Å) from an IμS 3.0 microfocus source with multilayer optics, at a low temperature (100 K). Bruker APEX3 Software Packages were used for structure solving and refinement. Hydrogen atoms were placed in fixed, idealized positions and refined with a riding model and a mutual isotropic thermal parameter [55]. The representations were generated using the Diamond program [56], and the intermolecular interactions were found with Platon [57,58].

## 4. Conclusions

[[(*Z*)-2′-{2-C_6_H_5_-(4*H*)-oxazol-5-one}CHC_6_H_4_]_2_Se (**5**) and [(*Z*)-4′-{2-C_6_H_5_-(4*H*)-oxazol-5-one}CHC_6_H_4_]_2_Se (**6**) were proven to be suitable ligands for the preparation of Ag(I), Au(I) and Zn(II) complexes. Ionic species in solution were obtained when AgOTf and AgPF_6_ were used in complexation reactions, while in the solid state, the triflate fragment in complexes **7** and **8** is bonded to the silver ions. No major differences were observed when a 1:2 molar ratio of the ligand and AgOTf was used for the preparation of **9** and **10**.

The coordination pattern of the ligand in [{Ag(OTf)}_2_{[(*Z*)-2′-{2-C_6_H_5_-(4*H*)-oxazol-5-one}CHC_6_H_4_]_2_Se}] (**9**) is 1κN:2κSe, as established by single-crystal X-ray studies, giving rise to a dimer, formed by two organoselenium ligands coordinated to a tetranuclear silver core. The silver core is built by triflate bridges between the silver atoms, with no Ag∙∙∙Ag argentophilic interactions and a distorted square-pyramidal coordination geometry around each silver center.

Supramolecular 2D and 3D architectures were observed in the crystals of **4**, **5** and **9**, supported by intermolecular C−H∙∙∙π, C−H∙∙∙O, Cl∙∙∙H and F∙∙∙H contacts.

UV-Vis absorption spectra showed the electron-donor property of the oxazolone ring by intraligand charge transfer (ILCT) characterized by large molar extinction coefficients for compounds **5**–**16**. A bathochromic shift in the UV-Vis spectra was observed when comparing the *para*-substituted derivatives with the related *ortho*-substituted derivatives. Although complexes **8** and **14** did not exhibit fluorescence, hyperchromism could be observed in the case of zinc complex **15**.

## Figures and Tables

**Table 1:** Photophysical data of compounds **3**–**16**.

Cpd.	λ_abs_ (nm) [ε (M^−1^·cm^−1^)]	λ_ex_ (nm)	λ_em_ (nm)	Stokes Shift (cm^−1^)
**3**	242 [30,806]; 271 [6805]; 344 [4659]	-	-	-
**4**	242 [18,414]; 313 [13,818]; 337 (sh)	-	-	-
**5**	255 [21,719]; 354 [28,900]; 367 [27,809]; 400 (sh)	400	523	5880
**6**	232 [12,818]; 262 [17,550]; 372 [25,677]; 404 [31,274]; 430 [sh]	430	510	3648
**7**	255 [13,485]; 354 [23,677]; 367 [22,836]; 400 (sh)	400	524	5916
**8**	265 [5357]; 372 [8840]; 399 [10,806]; 418 [11,154]	-	-	-
**11**	258 [23,581]; 365 [21,968]; 354 [22,844]; 400 (sh)	400	523	5880
**12**	264 [21,723]; 367 [29,273]; 401 [34,650]; 430 (sh)	430	511	3686
**13**	266 [16,567]; 354 [13,599]; 366 [13,134]; 400 (sh)	400	526	5989
**14**	265 [17,74]; 374 [21,201]; 399 [24,835]; 430 (sh)	-	-	-
**15**	266 [15,427]; 372 [31,308]; 404 [31,236]; 427 (sh)	427	523	4299
**16**	252 [18,387]; 355 [19,551]; 368 [18,930]; 395 (sh)	395	508	5631

**Table 2:** Selected interatomic distances (Å) and angles (°) in **9**·4CHCl_3_ ^a^.

Se1‒C1	1.943(8)	C1‒Se1‒C17	99.3(4)		
Se1‒C17	1.941(9)				
Ag1−N1	2.266(8)	N1−Ag1−O5	134.9(3)	Se1’−Ag2−O7	115.59(16)
Ag1−O5	2.254(7)	N1−Ag1−O8	126.9(2)	Se1’−Ag2−O10	125.86(16)
Ag1−O8	2.359(6)	N1−Ag1−O9’	83.03(22)	Se1’−Ag2−O8’	92.83(13)
Ag1−O9’	2.6514(56)	N1−Ag1−O10’	69.19(20)	Se1’−Ag2−O9’	119.69(13)
Ag1−O10’	3.5053(7)	O5−Ag1−O8	98.1(3)	O7−Ag2−O10	108.1(2)
Ag2−Se1’	2.5691(1)	O5−Ag1−O9’	107.77(24)	O7−Ag2−O8’	136.21(21)
Ag2−O7	2.336(6)	O5−Ag1−O10’	146.10(22)	O7−Ag2−O9’	86.5(2)
Ag2−O10	2.341(6)	O8−Ag1−O9’	84.02(22)	O10−Ag2−O8’	75.43(19)
Ag2−O8’	3.044(76)	O8−Ag1−O10’	66.14(2)	O10−Ag2−O9’	92.5(2)
Ag2−O9’	2.582(6)	O9’−Ag1−O10’	43.63(17)	O8’−Ag2−O9’	49.82(18)

^a^ Symmetry equivalent position (2−x, 1−y, 1−z) is given by “prime”.

## Data Availability

The data reported in this work are available in the published version of the manuscript and the corresponding Supplementary Material.

## Supplementary Materials

The following supporting information can be downloaded at https://www.mdpi.com/article/10.3390/molecules29040792/s1, Figures representing all NMR spectra; UV-Vis spectra; supramolecular architectures in the crystals of **4**, **5** and **9**·4CHCl_3_; X-ray crystal data and structure refinement for compounds. CCDC 2308731 (**5**), 2308732 (**4**) and 2308733 (**9**·4CHCl_3_).
