# Supplementary material for: Metal Complexes Containing Homoleptic Diorganoselenium(II) Ligands: Synthesis, Characterization and Investigation of Optical Properties
Source: Molecules. 2024 Feb 8;29(4):792. doi: 10.3390/molecules29040792 (PMC10892405; doi:10.3390/molecules29040792)

checkCIF/PLATON report

Structure factors have been supplied for datablock(s) ale274

THIS REPORT IS FOR GUIDANCE ONLY. IF USED AS PART OF A REVIEW PROCEDURE FOR PUBLICATION, IT SHOULD NOT REPLACE THE EXPERTISE OF AN EXPERIENCED CRYSTALLOGRAPHIC REFEREE.

No syntax errors found. CIF dictionary Interpreting this report

Datablock: ale274

```
Bond precision:   C-C = 0.0030 A          Wavelength=0.71073

Cell:            a=12.2490(4)     b=7.5386(3)        c=12.9332(5)
                 alpha=90         beta=102.563(1)    gamma=90
Temperature:     100 K

                Calculated                   Reported
Volume          1165.66(8)                   1165.66(8)
Space group     P 21/n                       P 1 21/n 1
Hall group      -P 2yn                       -P 2yn
Moiety formula  C14 H10 O2 Se                ?
Sum formula     C14 H10 O2 Se                C14 H10 O2 Se
Mr              289.18                       289.18
Dx,g cm-3       1.648                        1.648
Z               4                            4
Mu (mm-1)       3.206                        3.206
F000            576.0                        576.0
F000'           575.87
h,k,lmax                                     16,10,17
Nref                                         2888
Tmin,Tmax       0.670,0.828                  0.720,0.830
Tmin'           0.657

Correction method= # Reported T Limits: Tmin=0.720 Tmax=0.830
AbsCorr = MULTI-SCAN

Data completeness=                 Theta(max)= 28.300

R(reflections)= 0.0286( 2457)                        wR2(reflections)=
                                                     0.0709( 2888)
S = 1.044                  Npar= 154
```

The following ALERTS were generated. Each ALERT has the format
test-name_ALERT_alert-type_alert-level.
Click on the hyperlinks for more details of the test.

Alert level C

```
PLAT230_ALERT_2_C Hirshfeld Test Diff for    C4       --C7       .        5.4 s.u.
```

Alert level G

```
PLAT883_ALERT_1_G No Info/Value for _atom_sites_solution_primary .     Please Do !
PLAT978_ALERT_2_G Number C-C Bonds with Positive Residual Density.         13 Info
```

0 **ALERT level A** = Most likely a serious problem - resolve or explain
0 **ALERT level B** = A potentially serious problem, consider carefully
1 **ALERT level C** = Check. Ensure it is not caused by an omission or oversight
2 **ALERT level G** = General information/check it is not something unexpected

1 ALERT type 1 CIF construction/syntax error, inconsistent or missing data
2 ALERT type 2 Indicator that the structure model may be wrong or deficient
0 ALERT type 3 Indicator that the structure quality may be low
0 ALERT type 4 Improvement, methodology, query or suggestion
0 ALERT type 5 Informative message, check

It is advisable to attempt to resolve as many as possible of the alerts in all categories. Often the minor alerts point to easily fixed oversights, errors and omissions in your CIF or refinement strategy, so attention to these fine details can be worthwhile. In order to resolve some of the more serious problems it may be necessary to carry out additional measurements or structure refinements. However, the purpose of your study may justify the reported deviations and the more serious of these should normally be commented upon in the discussion or experimental section of a paper or in the "special_details" fields of the CIF. checkCIF was carefully designed to identify outliers and unusual parameters, but every test has its limitations and alerts that are not important in a particular case may appear. Conversely, the absence of alerts does not guarantee there are no aspects of the results needing attention. It is up to the individual to critically assess their own results and, if necessary, seek expert advice.

Publication of your CIF in IUCr journals

A basic structural check has been run on your CIF. These basic checks will be run on all CIFs submitted for publication in IUCr journals (*Acta Crystallographica*, *Journal of Applied Crystallography*, *Journal of Synchrotron Radiation*); however, if you intend to submit to *Acta Crystallographica Section C* or *E* or *IUCrData*, you should make sure that full publication checks are run on the final version of your CIF prior to submission.

Publication of your CIF in other journals

Please refer to the *Notes for Authors* of the relevant journal for any special instructions relating to CIF submission.

PLATON version of 14/11/2023; check.def file version of 14/09/2023

Datablock ale274 - ellipsoid plot

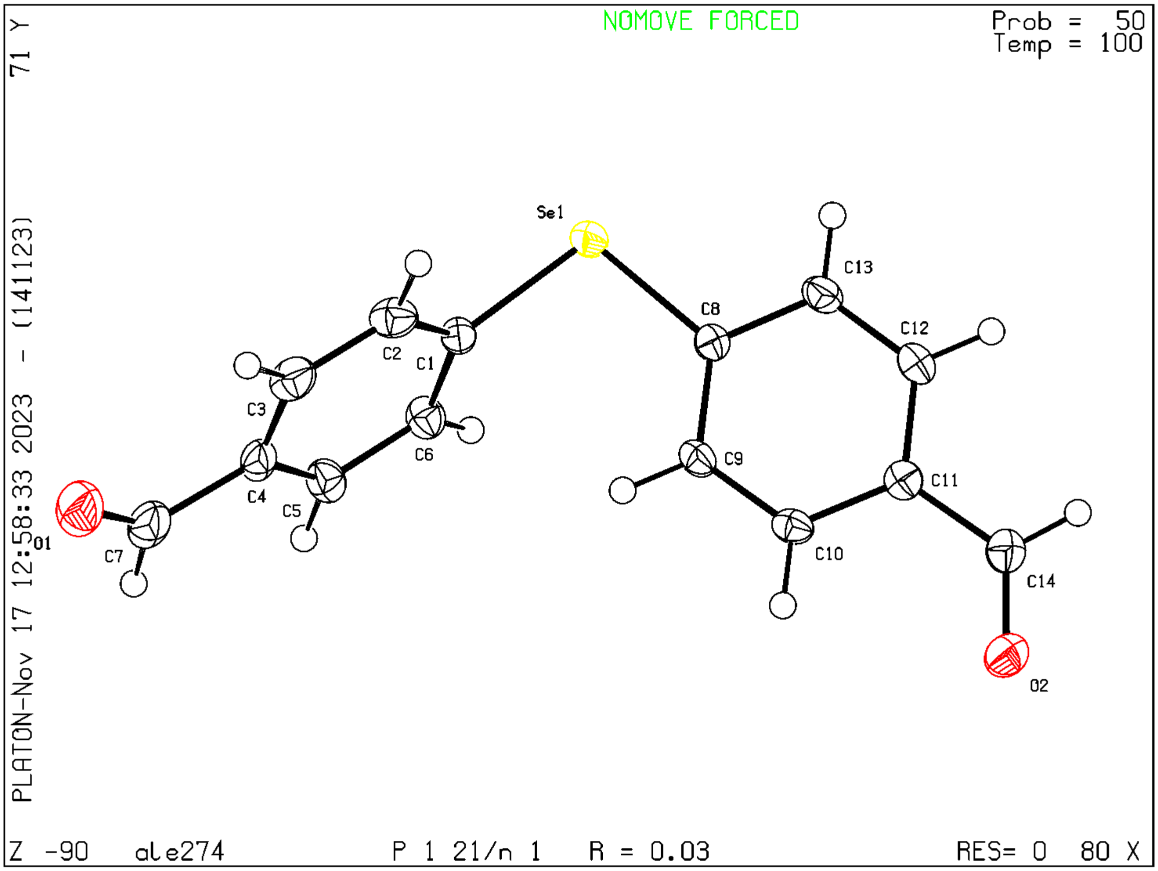

Supplement: Supplementary file 1 [file molecules-29-00792-s001.zip › Cpd_4_CCDC_2308732.pdf]
