# Supplementary material for: Metal Complexes Containing Homoleptic Diorganoselenium(II) Ligands: Synthesis, Characterization and Investigation of Optical Properties
Source: Molecules. 2024 Feb 8;29(4):792. doi: 10.3390/molecules29040792 (PMC10892405; doi:10.3390/molecules29040792)

checkCIF/PLATON report

Structure factors have been supplied for datablock(s) ale288

THIS REPORT IS FOR GUIDANCE ONLY. IF USED AS PART OF A REVIEW PROCEDURE FOR PUBLICATION, IT SHOULD NOT REPLACE THE EXPERTISE OF AN EXPERIENCED CRYSTALLOGRAPHIC REFEREE.

No syntax errors found. CIF dictionary Interpreting this report

Datablock: ale288

Bond precision: C-C = 0.0132 A Wavelength=0.71073

Cell:	a=11.2955(13)	b=13.2811(15)	c=17.903(2)
	alpha=84.806(3)	beta=81.693(3)	gamma=88.358(4)
Temperature:	100 K		

	Calculated	Reported
Volume	2646.3(5)	2646.3(5)
Space group	P -1	P -1
Hall group	-P 1	-P 1
Moiety formula	C68 H40 Ag4 F12 N4 O20 S4 Se2, 8(C H Cl3)	?
Sum formula	C76 H48 Ag4 Cl24 F12 N4 O20 S4 Se2	C76 H48 Ag4 Cl24 F12 N4 O20 S4 Se2
Mr	3133.63	3133.62
Dx,g cm-3	1.966	1.966
Z	1	1
Mu (mm-1)	2.188	2.188
F000	1528.0	1528.0
F000’	1528.92	
h,k,lmax		13,15,21
Nref		9332
Tmin,Tmax	0.845,0.951	0.780,0.950
Tmin’	0.838	

Correction method= # Reported T Limits: Tmin=0.780 Tmax=0.950
AbsCorr = MULTI-SCAN

Data completeness= Theta(max)= 25.000

R(reflections)= 0.0669(6615) wR2(reflections)= 0.1764(9332)

S = 1.036 Npar= 658

The following ALERTS were generated. Each ALERT has the format
test-name_ALERT_alert-type_alert-level.
Click on the hyperlinks for more details of the test.

Alert level C

```
RINTA01_ALERT_3_C  The value of Rint is greater than 0.12
            Rint given   0.121
PLAT213_ALERT_2_C Atom O5               has ADP max/min Ratio .....        3.1 prolat
PLAT234_ALERT_4_C Large Hirshfeld Difference C11      --C16      .       0.16 Ang.
PLAT241_ALERT_2_C High   'MainMol' Ueq as Compared to Neighbors of         O5 Check
PLAT242_ALERT_2_C Low    'MainMol' Ueq as Compared to Neighbors of         S1 Check
PLAT244_ALERT_4_C Low    'Solvent' Ueq as Compared to Neighbors of        C35 Check
PLAT244_ALERT_4_C Low    'Solvent' Ueq as Compared to Neighbors of        C36 Check
PLAT244_ALERT_4_C Low    'Solvent' Ueq as Compared to Neighbors of        C37 Check
PLAT342_ALERT_3_C Low Bond Precision on  C-C Bonds ..............    0.01319 Ang.
PLAT906_ALERT_3_C Large K Value in the Analysis of Variance ......      5.706 Check
PLAT971_ALERT_2_C Check Calcd Resid. Dens.  1.17Ang From Cl7            2.47 eA-3
PLAT971_ALERT_2_C Check Calcd Resid. Dens.  1.08Ang From Ag2            1.81 eA-3
PLAT971_ALERT_2_C Check Calcd Resid. Dens.  1.11Ang From Ag2            1.51 eA-3
PLAT972_ALERT_2_C Check Calcd Resid. Dens.  0.82Ang From Cl7           -1.56 eA-3
```

Alert level G

```
PLAT020_ALERT_3_G The Value of Rint is Greater Than 0.12 .........      0.121 Report
PLAT083_ALERT_2_G SHELXL Second Parameter in WGHT  Unusually Large      33.54 Why ?
PLAT242_ALERT_2_G Low    'MainMol' Ueq as Compared to Neighbors of        C33 Check
PLAT242_ALERT_2_G Low    'MainMol' Ueq as Compared to Neighbors of        C34 Check
PLAT398_ALERT_2_G Deviating  C-O-C   Angle From 120 for O1       .      105.0 Degree
PLAT398_ALERT_2_G Deviating  C-O-C   Angle From 120 for O3       .      105.3 Degree
PLAT431_ALERT_2_G Short Inter HL..A Contact  Cl10     ..O3       .       3.13 Ang.
                                             1-x,-y,2-z  =      2_657 Check
PLAT790_ALERT_4_G Centre of Gravity not Within Unit Cell: Resd.  #          4 Note
              C H Cl3
PLAT883_ALERT_1_G No Info/Value for _atom_sites_solution_primary .     Please Do !
PLAT909_ALERT_3_G Percentage of I>2sig(I) Data at Theta(Max) Still        42% Note
PLAT910_ALERT_3_G Missing # of FCF Reflection(s) Below Theta(Min).          4 Note
                1  0  0,   0  1  0,   0  0  1,   0  1  1,
PLAT961_ALERT_5_G Dataset Contains no Negative Intensities .......     Please Check
PLAT967_ALERT_5_G Note: Two-Theta Cutoff Value in Embedded .res ..       50.0 Degree
PLAT978_ALERT_2_G Number C-C Bonds with Positive Residual Density.          0 Info
```

---

0 **ALERT level A** = Most likely a serious problem - resolve or explain
0 **ALERT level B** = A potentially serious problem, consider carefully
14 **ALERT level C** = Check. Ensure it is not caused by an omission or oversight
14 **ALERT level G** = General information/check it is not something unexpected

1 ALERT type 1 CIF construction/syntax error, inconsistent or missing data

```
 14 ALERT type 2 Indicator that the structure model may be wrong or deficient
  6 ALERT type 3 Indicator that the structure quality may be low
  5 ALERT type 4 Improvement, methodology, query or suggestion
  2 ALERT type 5 Informative message, check
```

It is advisable to attempt to resolve as many as possible of the alerts in all categories. Often the minor alerts point to easily fixed oversights, errors and omissions in your CIF or refinement strategy, so attention to these fine details can be worthwhile. In order to resolve some of the more serious problems it may be necessary to carry out additional measurements or structure refinements. However, the purpose of your study may justify the reported deviations and the more serious of these should normally be commented upon in the discussion or experimental section of a paper or in the "special_details" fields of the CIF. checkCIF was carefully designed to identify outliers and unusual parameters, but every test has its limitations and alerts that are not important in a particular case may appear. Conversely, the absence of alerts does not guarantee there are no aspects of the results needing attention. It is up to the individual to critically assess their own results and, if necessary, seek expert advice.

Publication of your CIF in IUCr journals

A basic structural check has been run on your CIF. These basic checks will be run on all CIFs submitted for publication in IUCr journals (*Acta Crystallographica*, *Journal of Applied Crystallography*, *Journal of Synchrotron Radiation*); however, if you intend to submit to *Acta Crystallographica Section C* or *E* or *IUCrData*, you should make sure that full publication checks are run on the final version of your CIF prior to submission.

Publication of your CIF in other journals

Please refer to the *Notes for Authors* of the relevant journal for any special instructions relating to CIF submission.

PLATON version of 14/11/2023; check.def file version of 14/09/2023

Datablock ale288 - ellipsoid plot

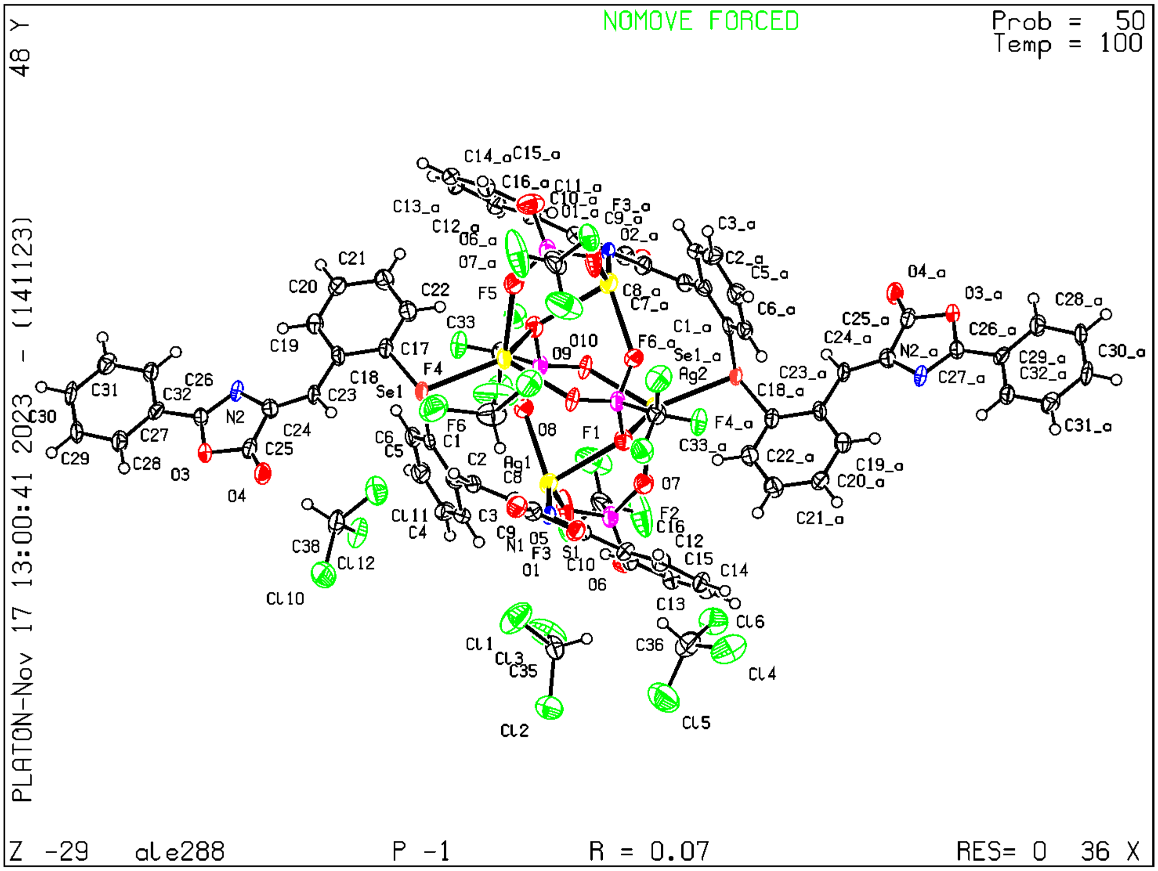

Supplement: Supplementary file 1 [file molecules-29-00792-s001.zip › Cpd_9_CCDC_ 2308733.pdf]
