# Supplementary material for: Metal Complexes Containing Homoleptic Diorganoselenium(II) Ligands: Synthesis, Characterization and Investigation of Optical Properties
Source: Molecules. 2024 Feb 8;29(4):792. doi: 10.3390/molecules29040792 (PMC10892405; doi:10.3390/molecules29040792)
Supplement: Supplementary file 1 [file molecules-29-00792-s001.zip › Molecules_ESI_2825259.pdf]

Molecules

Electronic Supporting Information (ESI) for

Metal Complexes Containing Homoleptic Diorganoselenium(II) Ligands: Synthesis, Characterization and Investigation of Optical Properties

Darius Dumitraş,[1] Emese Gal,[2] Cristian Silvestru[1] and Alexandra Pop[1,*]

[1] Supramolecular Organic and Organometallic Chemistry Centre, Department of Chemistry, Faculty of Chemistry and Chemical Engineering, Babeş-Bolyai University, Str. Arany Janos 11, RO-400028 Cluj-Napoca, România.

[2] Research Center on Fundamental and Applied Heterochemistry, Faculty of Chemistry and Chemical Engineering, Babeș-Bolyai University, Str. Arany Janos 11, RO-400028, Cluj-Napoca, România

Corresponding author: Alexandra Pop

E-mail: alexandra.m.pop@ubbcluj.ro; Tel: (+40) 264-593833.

Contents

Figures and Schemes
Figure S1. ^{1}H NMR spectra ($CDCl_3$) of **1** (a), **3** (b) and **5** (c)…………………………**S3**
Figure S2. ^{1}H NMR spectra ($CDCl_3$) of **2** (a), **4** (b) and **6** (c) ....…**S4**
Figure S3. ^{1}H NMR spectra of **5**, **7**, **11**, **13** and **15**……………………………………..S**4**
Figure S4. ^{1}H NMR spectra of **6**, **8**, **12**, **14** and **16**………………………………………S**5**
Figure S5. UV–Vis absorption spectra of **5** and **6**……………………………………………..S**5**
Figure S6. Absorption and emission spectra of **5** and **6**...S**6**

X-ray diffraction crystallography...S**6**
Figure S7. View of the supramolecular 3D network in **4**...S**6**
Figure S8. View of the supramolecular 2D network in **5**...S**7**
Figure S9. Polymeric chain association in the crystal of **9** **S7**
Figure S10. View of the supramolecular 2D network in **9**...S**8**
Table S1. Crystal data and details of data collection for **4**, **5** and **9**…………………... S**9**

NMR spectra
Figures S11-S12, ^{1}H and ^{77}Se{^{1}H} NMR spectra of **1**.. S**10**
Figure S13-S15, ^{1}H, ^{13}C{^{1}H} and ^{77}Se{^{1}H} NMR spectra of **2**...................................S**11**
Figure S16, ^{1}H NMR spectrum of **3**..S**12**
Figure S17-S19, ^{1}H, ^{13}C{^{1}H} and ^{77}Se{^{1}H} NMR spectra of **4**...................................S**13**
Figure S120-22, ^{1}H, ^{13}C{^{1}H} and ^{77}Se{^{1}H} NMR spectra of **5**...................................S**14**
Figure S23-25, ^{1}H, ^{13}C{^{1}H} and ^{77}Se{^{1}H} NMR spectra of **6**.....................................S**16**
Figure S26-28, ^{1}H, ^{13}C{^{1}H} and ^{19}F{^{1}H} NMR spectrum of **7** S**17**
Figure S29-31, ^{1}H, ^{13}C{^{1}H} and ^{19}F{^{1}H} NMR spectrum of **9** S**19**
Figure S32-34, ^{1}H, ^{13}C{^{1}H} and ^{19}F{^{1}H} NMR spectrum of **8** S**20**
Figure S35-37, ^{1}H, ^{13}C{^{1}H} and ^{19}F{^{1}H} NMR spectrum of **10** S**22**
Figure S38-41, ^{1}H, ^{13}C{^{1}H}, ^{19}F{^{1}H} and ^{31}P{^{1}H} NMR spectrum of **11**...................S**23**
Figures S42-S45, ^{1}H, ^{13}C{^{1}H}, ^{19}F{^{1}H} and ^{31}P{^{1}H} NMR spectra of **12**..................S**25**
Figure S46-48, ^{1}H, ^{13}C{^{1}H} and ^{77}Se{^{1}H} NMR spectra of **13**...................................S**27**
Figure S49-51, ^{1}H, ^{13}C{^{1}H} and ^{77}Se{^{1}H} NMR spectra of **14**...................................S**29**
Figure S52-53, ^{1}H and ^{13}C{^{1}H} NMR spectra of **15**..S**30**
Figure S54-56, ^{1}H, ^{13}C{^{1}H} and ^{77}Se{^{1}H} NMR spectra of **16**...................................S**31**

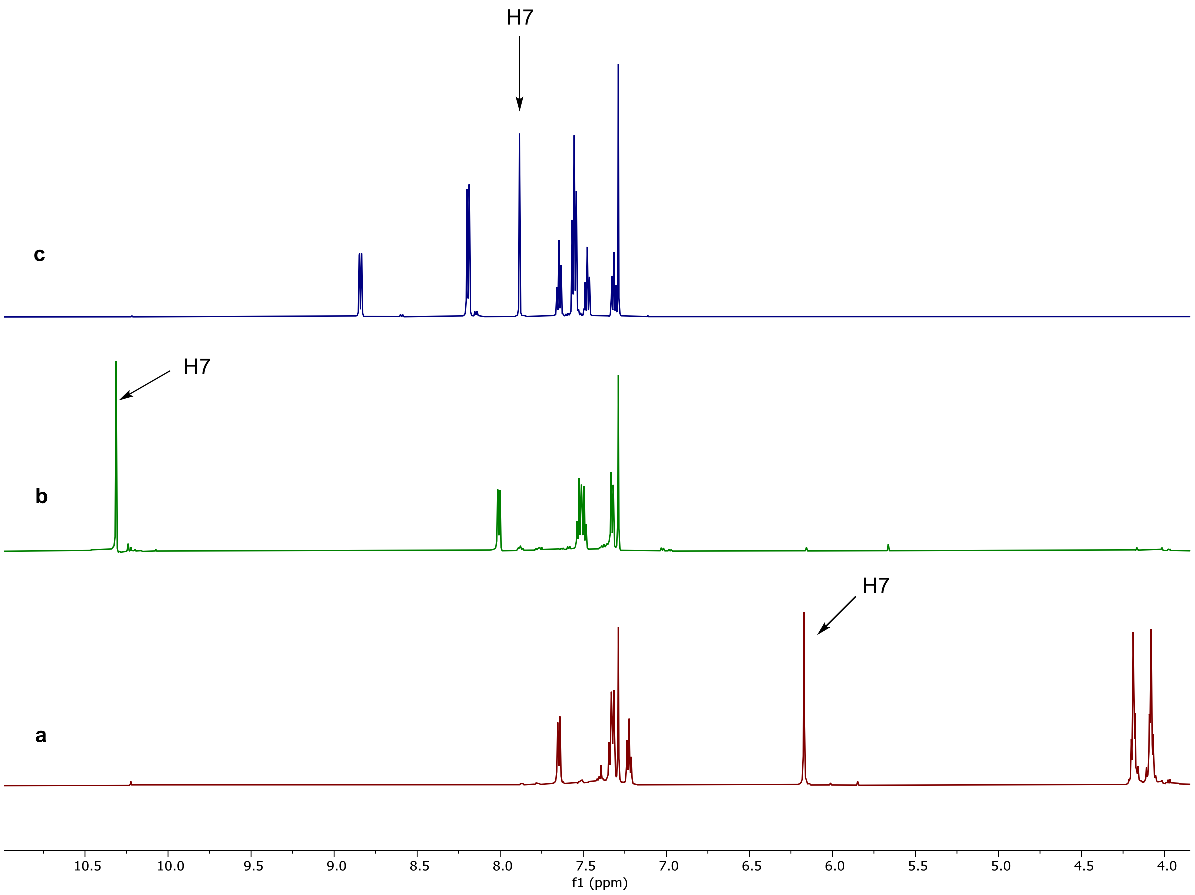

Figure S1. ^{1}H NMR spectra ($CDCl_3$) of **1** (a), **3** (b) and **5** (c).

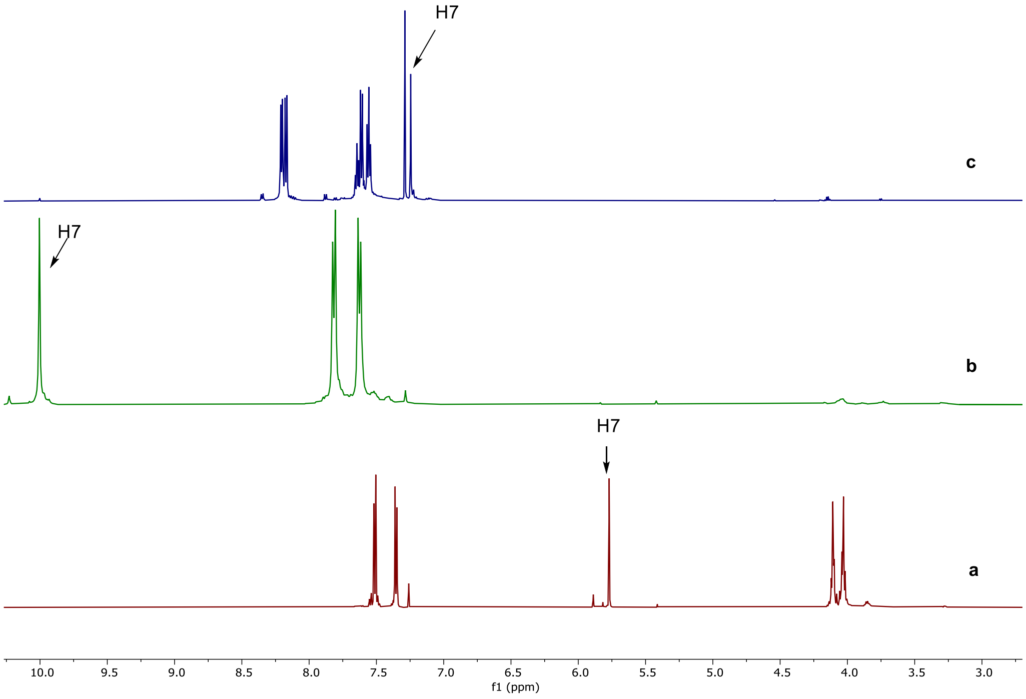

Figure S2. ^{1}H NMR spectra ($CDCl_3$) of **2** (a), **4** (b) and **6** (c).

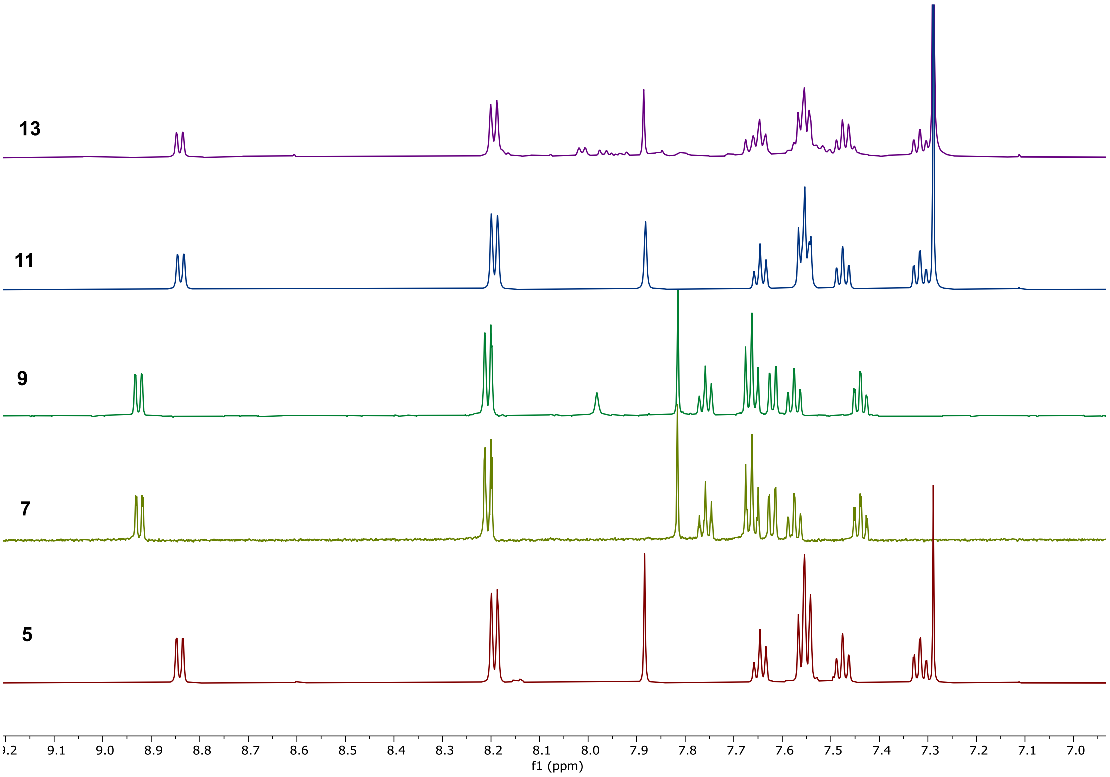

Figure S3. ^{1}H NMR spectra of **5** ($CDCl_3$), **7** (acetone-d_6), **11** (acetone-d_6), **13** ($CDCl_3$) and **15** ($CDCl_3$).

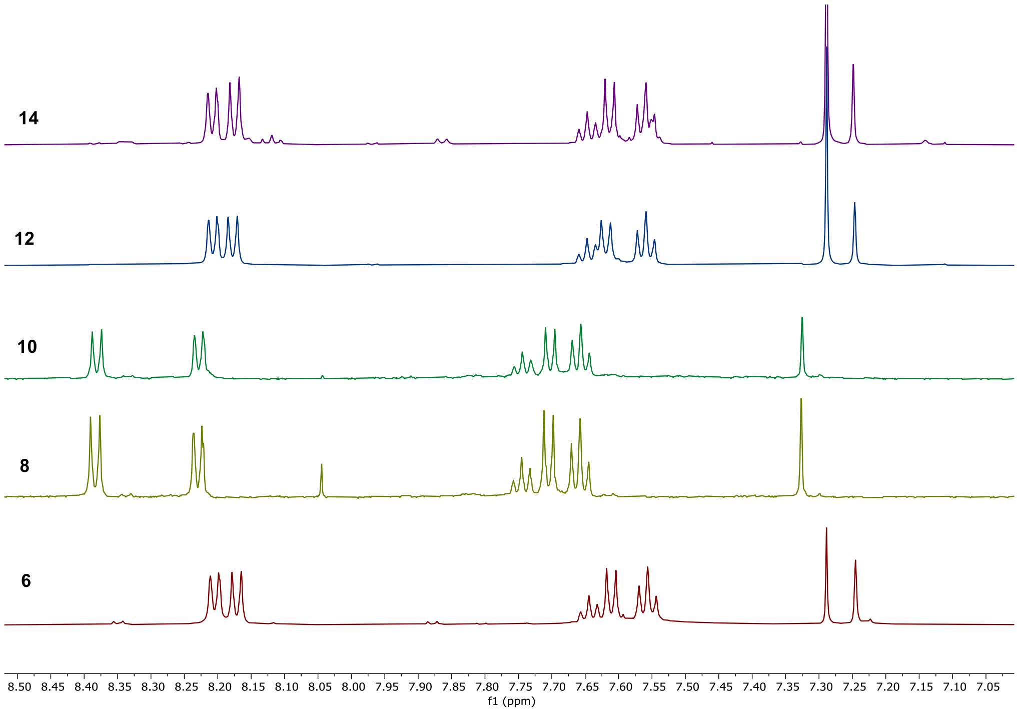

Figure S4. ^{1}H NMR spectra of **6** ($CDCl_3$), **8** (acetone-d_6), **12** (acetone-d_6), **14** ($CDCl_3$) and **16** ($CDCl_3$).

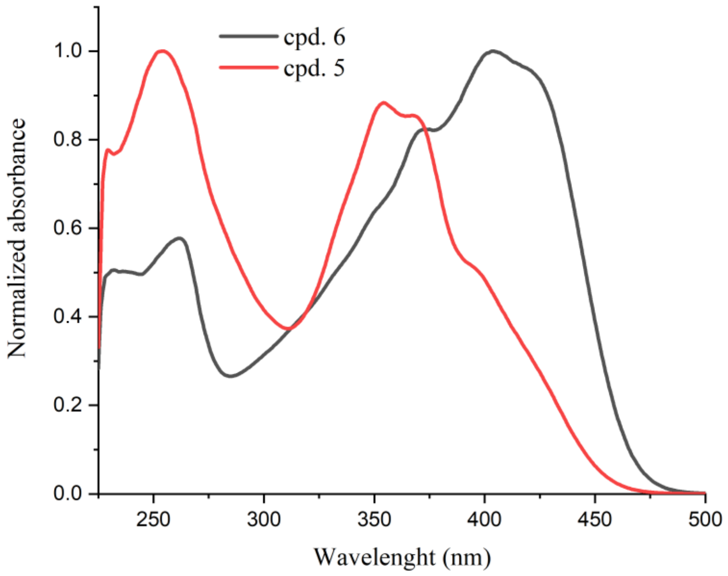

Figure S5. UV–Vis absorption spectra of **5** and **6**.

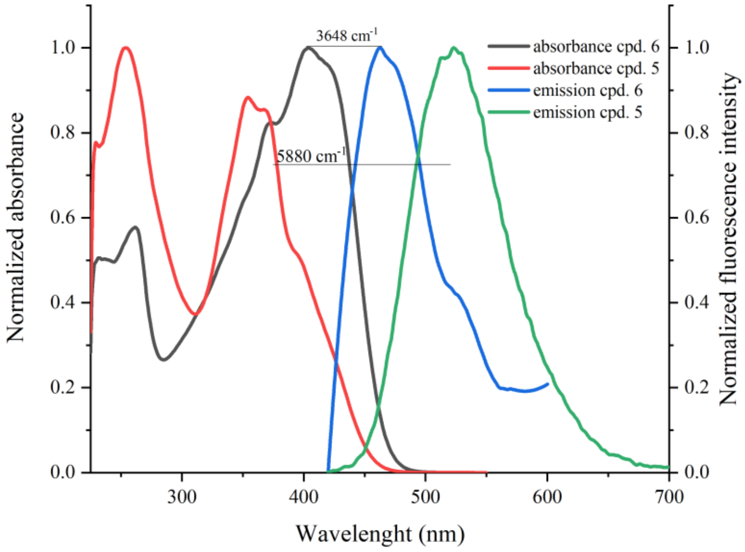

Figure S6. Absorption and emission spectra of **5** and **6**.

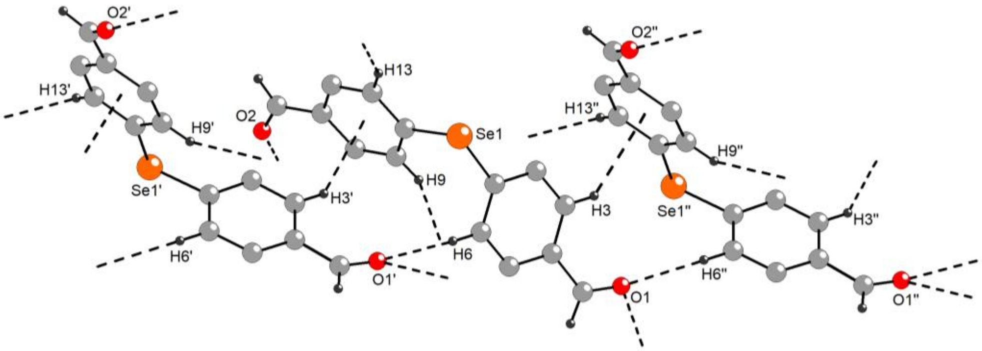

(a)

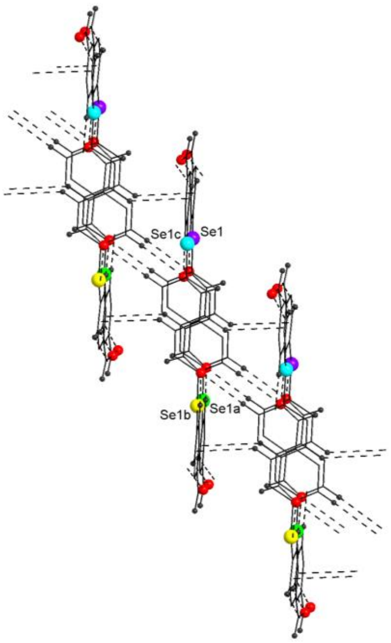

(b)

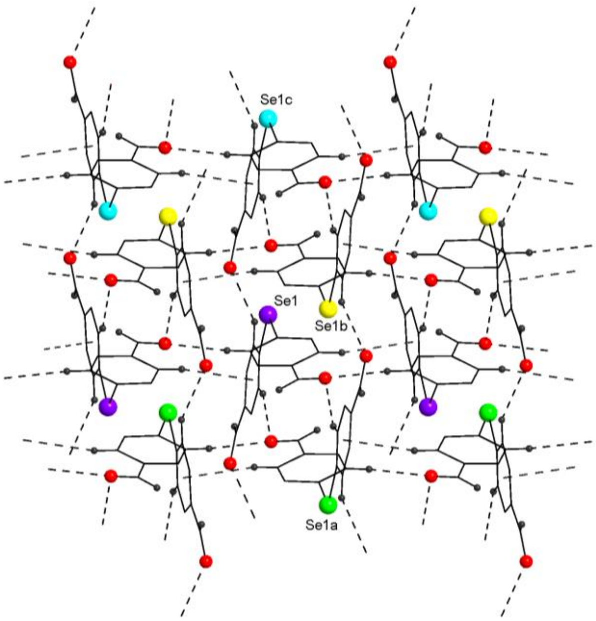

(c)

Figure S7. (a) View of a chain polymer in the crystal of **4** showing the interactions with the other neighbouring parallel chains (only hydrogen atoms involved in intermolecular interactions are shown); (b) view along axis *b*, and (c) view along axis *c* of the supramolecular 3D network build through $C-H_{aryl}\cdots\pi$ ($Ph_{centroid}$) and $C-H_{aryl}\cdots O$ interactions in the crystal of **4**; molecules of the same polymeric chain are indicated by same colour (green, magenta, yellow and cyan, respectively) for the corresponding selenium atoms [symmetry equivalent atoms (1−x, 1−y, 1−z), (1−x, 2−y, 1−z) and (x, 1+y, z) are given by “a” (green), “b” (yellow), and “c” (cyan), respectively].

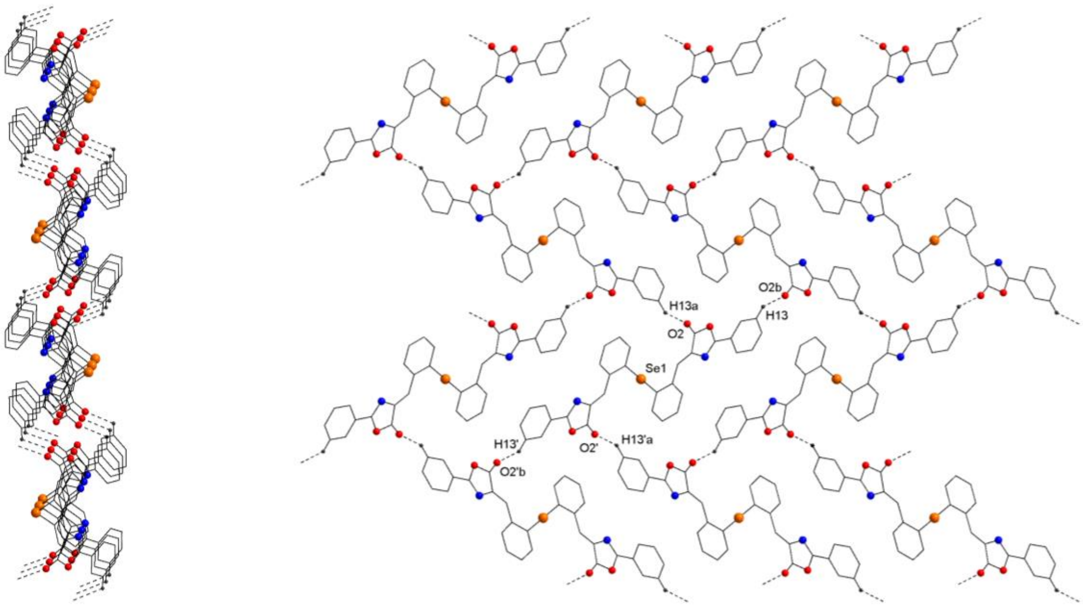

(a) (b)

Figure S8. (a) View along axis *a*, and (b) view along axis *c* of the supramolecular 2D network build through C−H_{aryl}···O interactions in the crystal of **5** [symmetry equivalent atoms (−x, 1−y, z), (−1/2+x, 3/2−y, 1−z), (1/2+x, 3/2−y, 1−z), (1/2−x, −1/2+y, 1−z) and (−1/2−x, −1/2+y, 1−z) are given by “prime”, “a”, “b”, “prime a” and “prime b”, respectively]

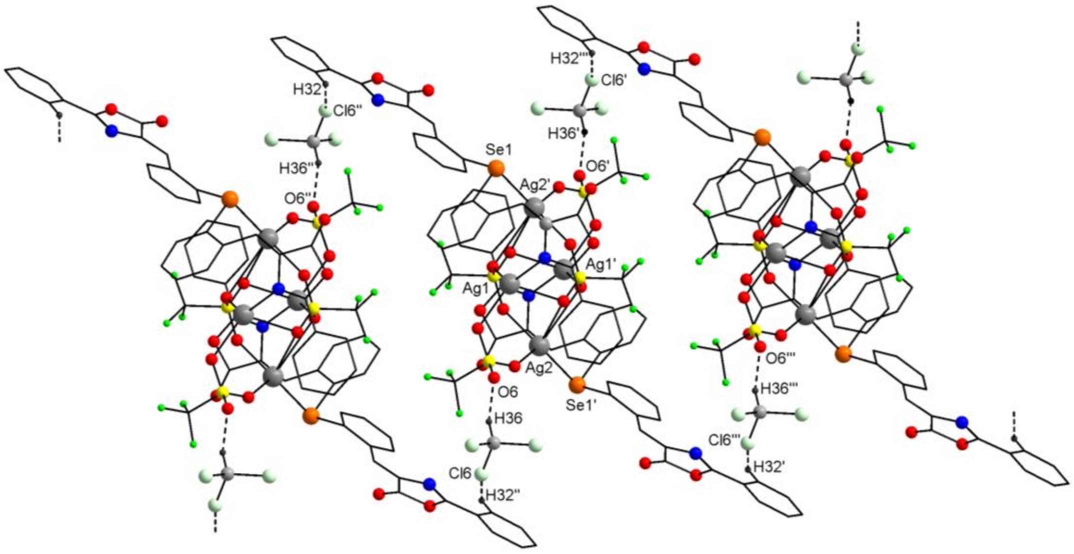

Figure S9. Polymeric chain association in the crystal of **9** (only hydrogen atoms involved in intermolecular interactions are shown). Symmetry equivalent atoms (2−x, 1−y, 1−z), (1−x, 1−y, 1−z), and (1+x, y, z) are given by “prime”, “double prime”, and “triple prime”, respectively.

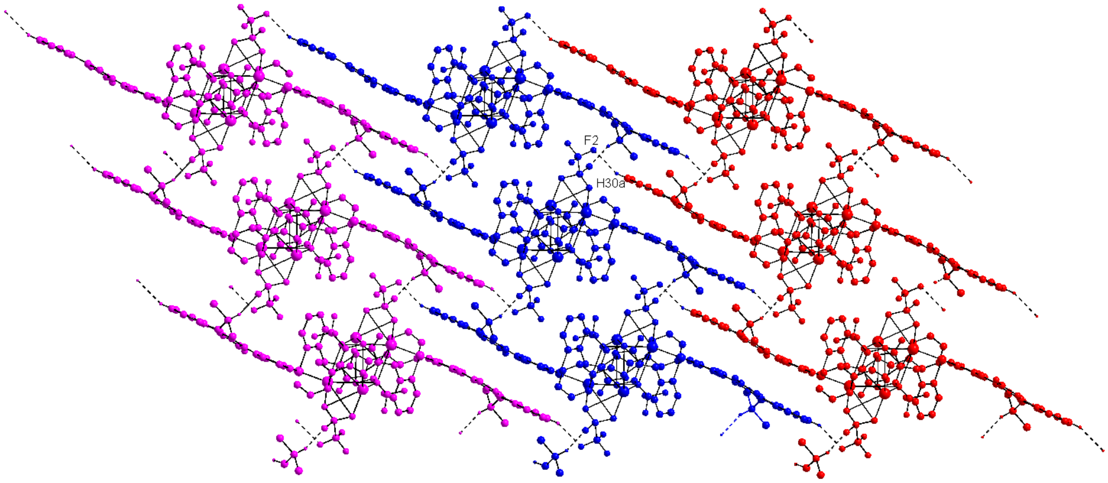

Figure S10. View of the supramolecular 2D network build through H···F interactions in the crystal of **9** (only hydrogen atoms involved in intermolecular interactions are shown). Symmetry equivalent atoms (1+x, y, −1+z) are given by “a”. F2···H30a 2.63 Å.

Table S1. Crystal data and details of data collection for **4**, **5** and **9**.

Compound	**4**	**5**	**9**
Empirical formula	$C_{14}H_{10}O_2Se$	$C_{32}H_{20}N_2O_4Se$	$C_{76}H_{48}Ag_4F_{12}N_4O_{20}Se_2$
Formula weight	289.18	575.46	3133.62
T [K]	100.(2)	100.(2)	100.(2)
Crystal system	monoclinic	orthorhombic	triclinic
Space group	P21/n	P 21 21 2	P-1
a [Å]	12.2490(4)	13.7518(6)	11.2955(13)
b [Å]	7.5386(3)	19.5095(10)	13.2811(15)
c [Å]	12.9332(5)	4.7253(3)	17.903(2)
α [°]	90	90	84.806(3)
β [°]	102.5630(10)	90	81.693(3)
γ [°]	90	90	88.358(4)
V [Å^3]	1165.66(8)	1267.75(12)	2646.3(5)
Z	4	2	1
ρ_{calcd} [g cm^{-3}]	1.648	1.508	1.966
Absorption coefficient [mm^{-1}]	3.206	1.524	2.188
Crystal size [mm]	0.128x0.115x0.059	0.131x0.087x0.045	0.081x0.064x 0.023
Θ range for data collection	2.08 to 28.30	2.088 to 28.306	2.0 to 25.0
Reflections collected	33053	37153	106466
Independent reflections	2888	3165	9332
	[R(int) = 0.0470]	[R(int) = 0.0723]	[R(int) = 0.1210]
Data/restraints/parameters	2888/0/154	3165/0/178	9332/0/658
Final R indices [I>2sigma(I)]	R1 = 0.0286	R1 = 0.0325	R1 = 0.0669
	wR2 = 0.0663	wR2 = 0.0558	wR2 = 0.1609
R indices (all data)	R1 = 0.0379	R1 = 0.0451	R1 = 0.1046
	wR2 = 0.0709	wR2 = 0.0598	wR2 = 0.1764
Goodness-of-fit on F^2	1.044	1.043	1.036
Largest diff. peak/hole / e Å^{-3}	0.869 / -0.500	0.256 / -0.502	2.605 / -1.647

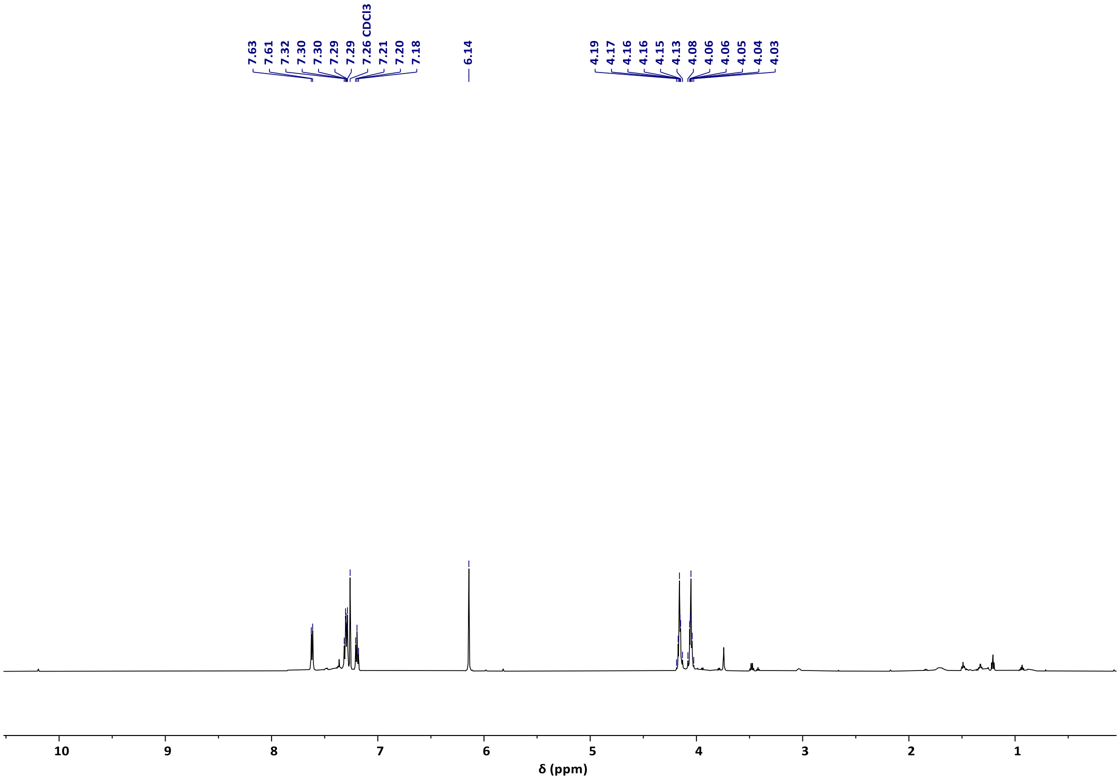

Figure S11. ^{1}H NMR spectrum ($CDCl_3$, 600 MHz) of **1**.

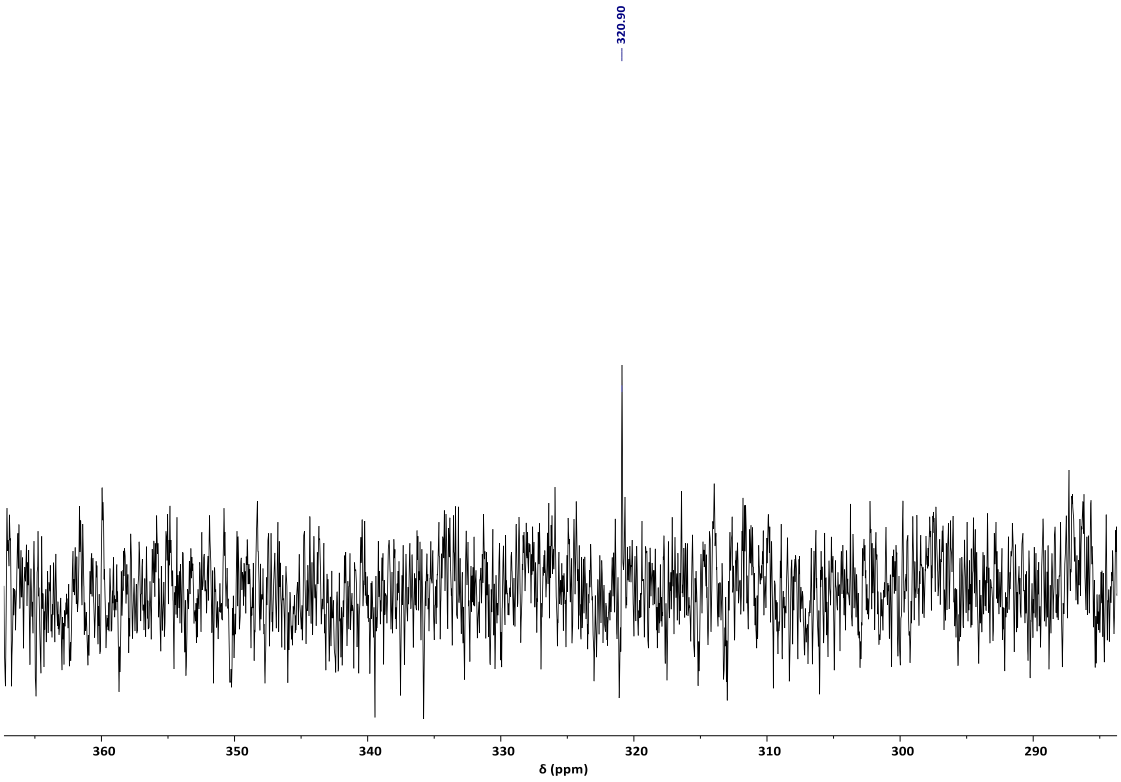

Figure S12. ^{77}Se NMR spectrum ($CDCl_3$, 115 MHz) of **1**.

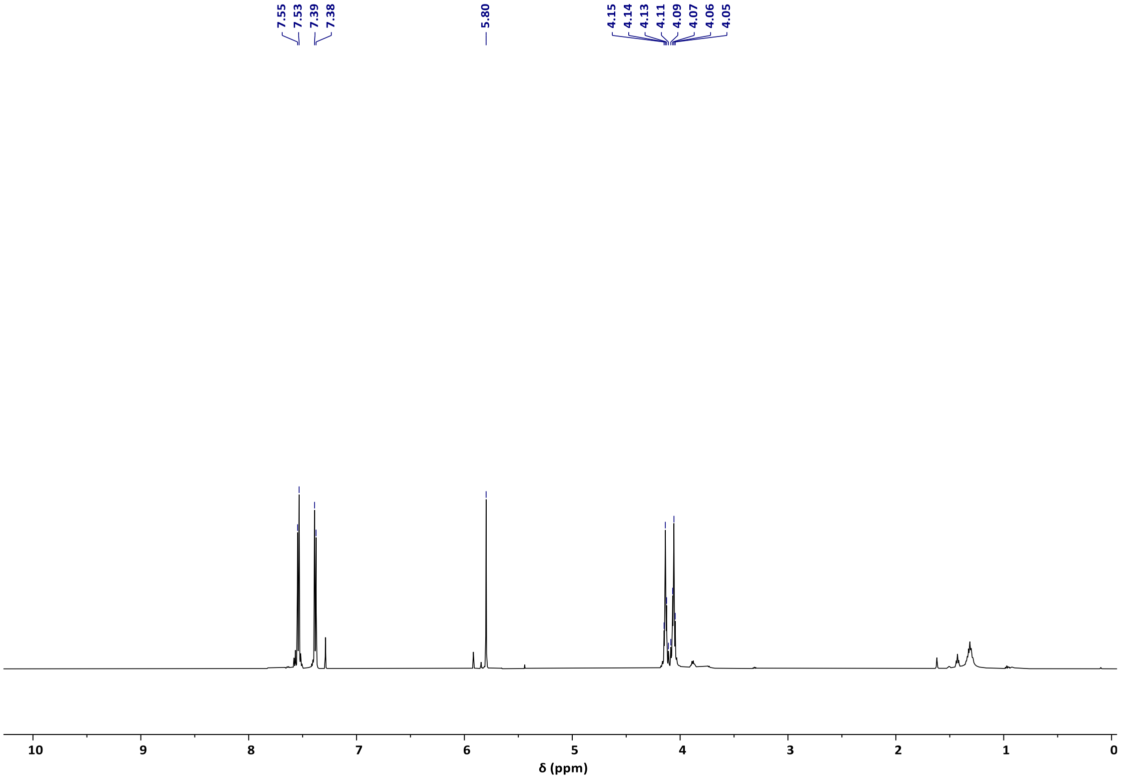

Figure S13. ^{1}H NMR spectrum ($CDCl_3$, 600 MHz) of **2**.

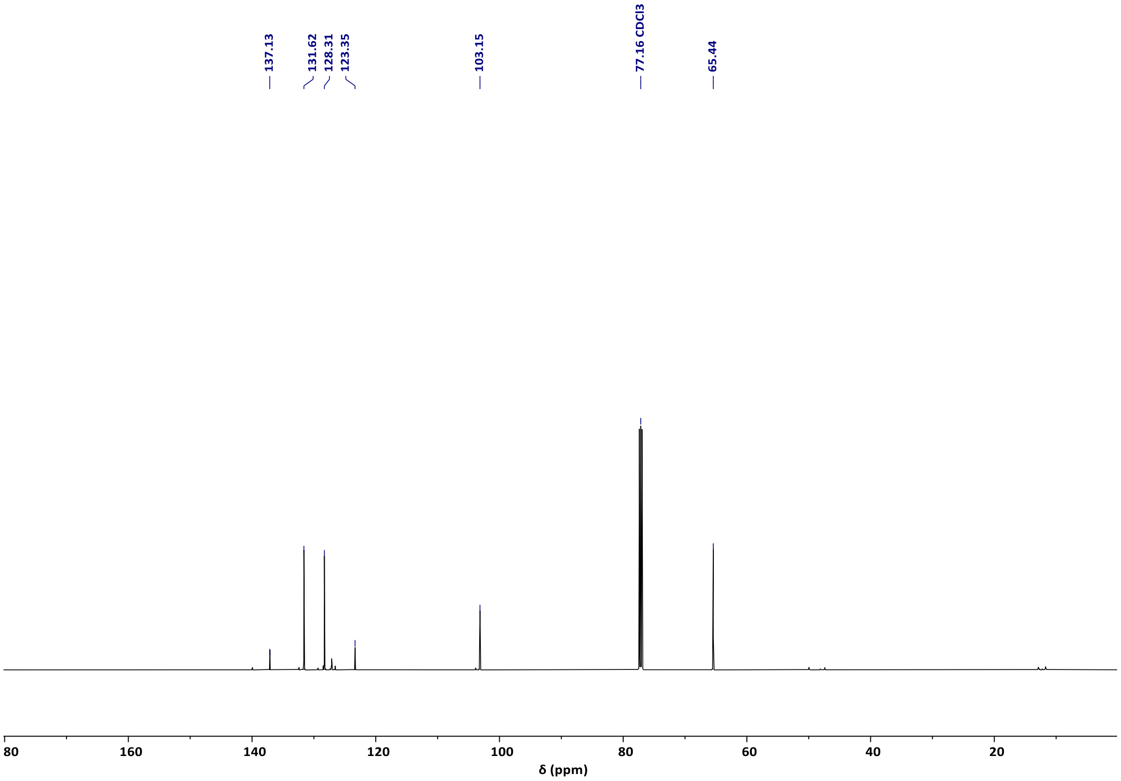

Figure S14. ^{13}C NMR spectrum ($CDCl_3$, 151 MHz) of **2**.

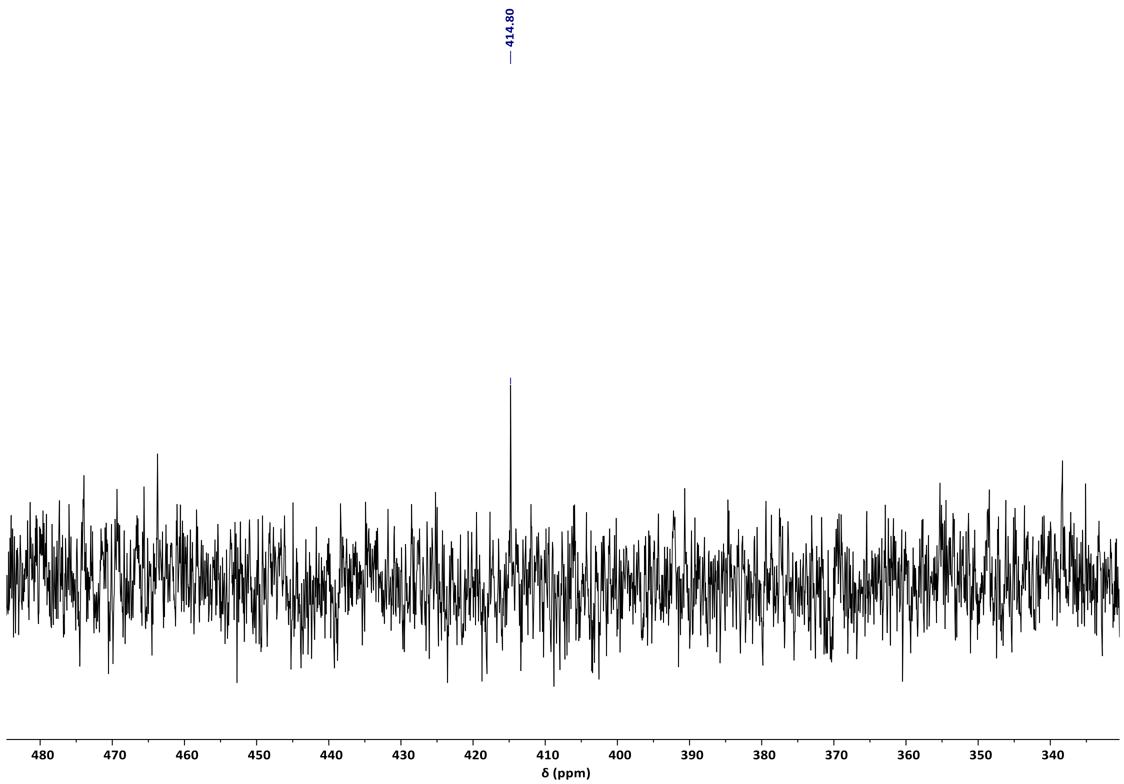

Figure S15. ^{77}Se NMR spectrum ($CDCl_3$, 114 MHz) of **2**.

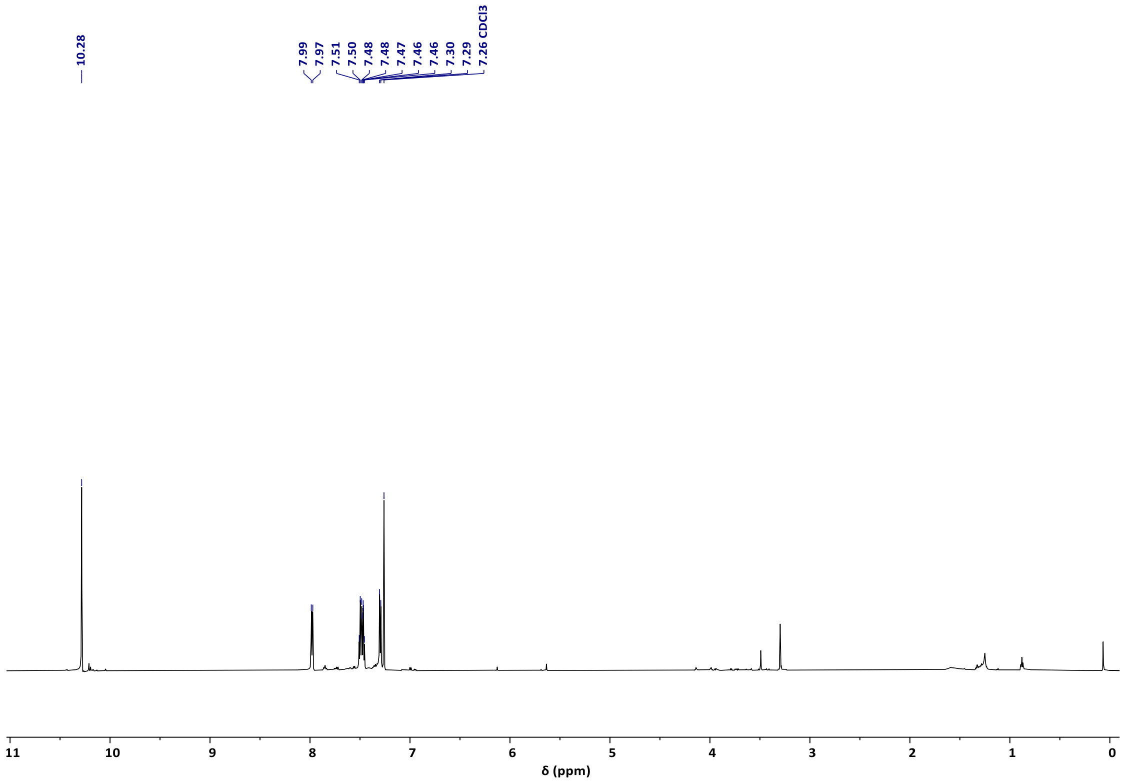

Figure S16. ^{1}H NMR spectrum ($CDCl_3$, 600 MHz) of **3**.

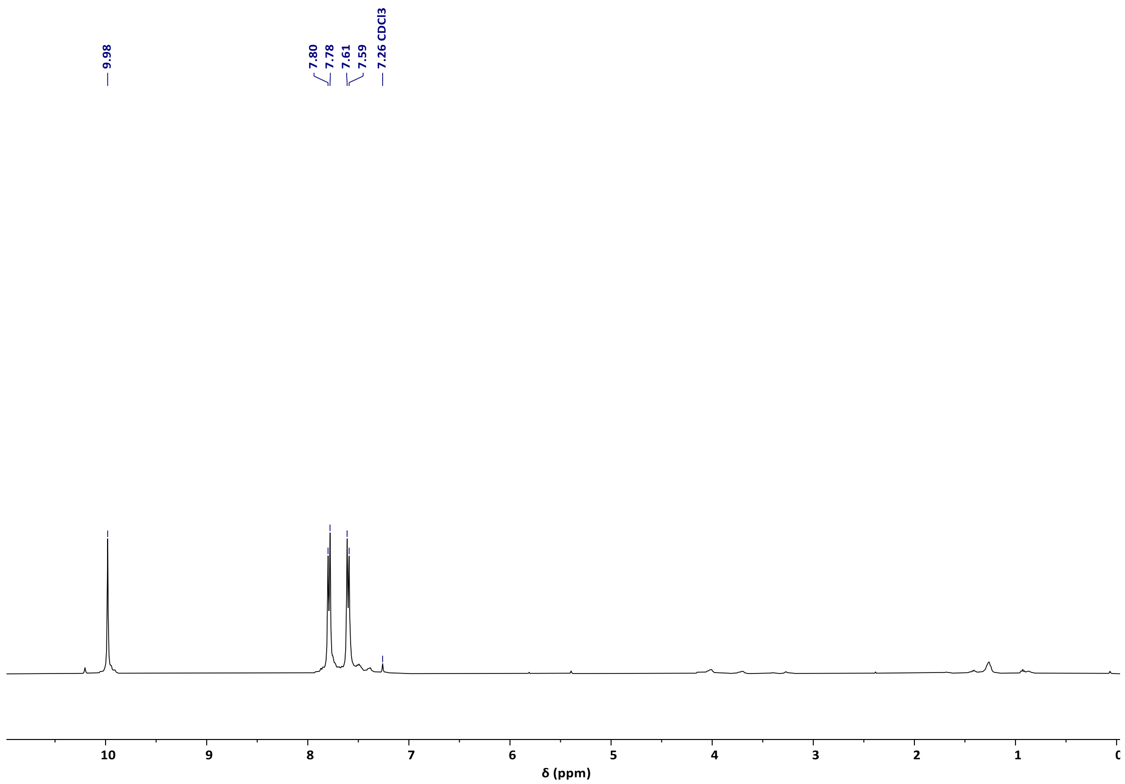

Figure S17. ^{1}H NMR spectrum ($CDCl_3$, 400 MHz) of **4**.

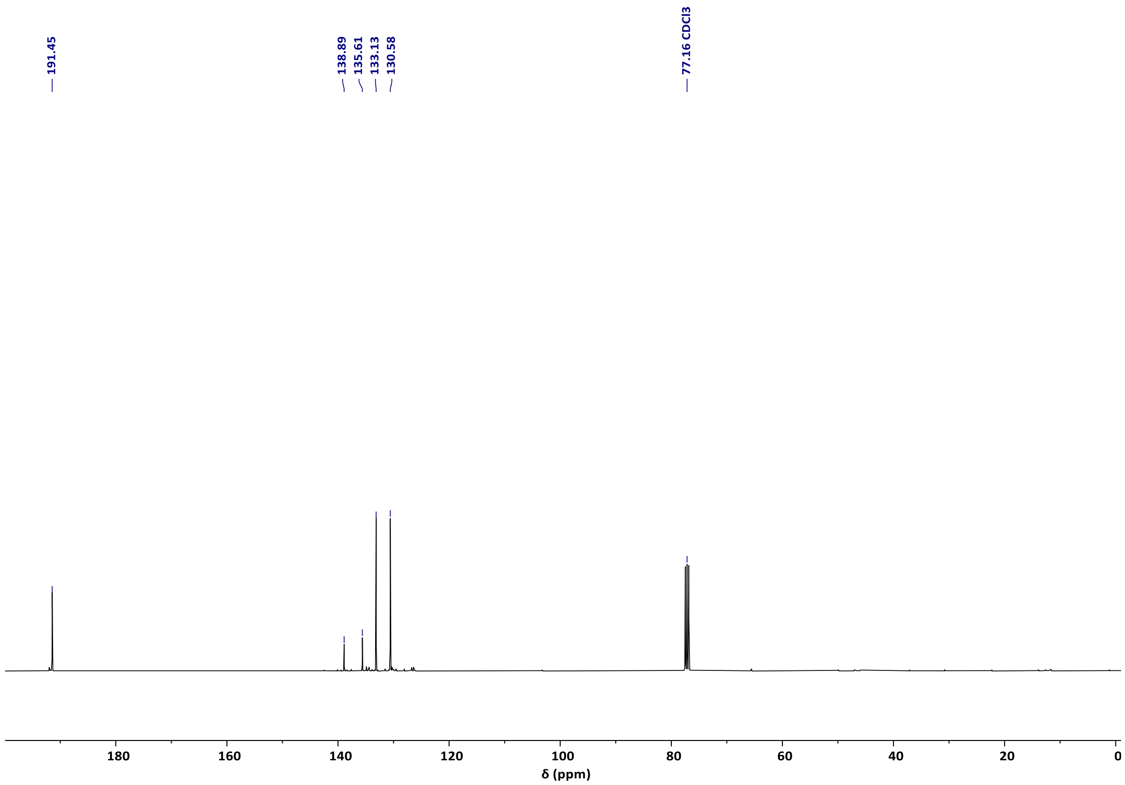

Figure S18. ^{13}C NMR spectrum ($CDCl_3$, 101 MHz) of **4**.

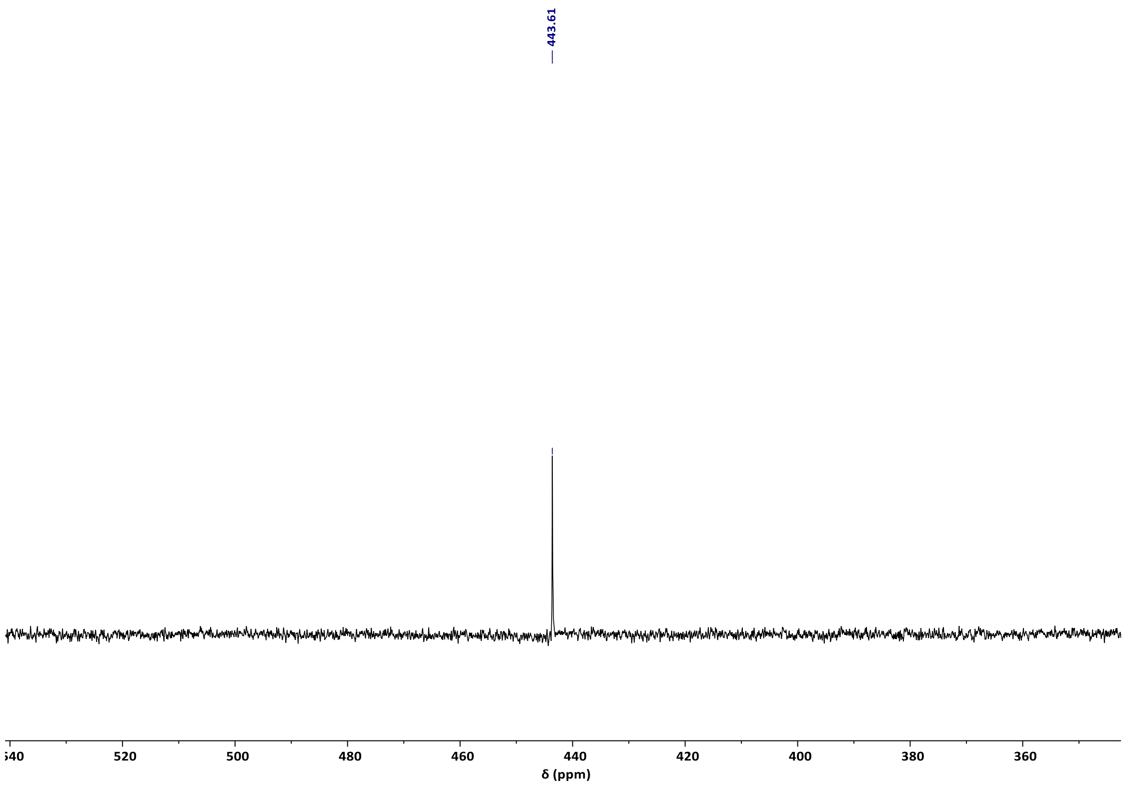

Figure S19. ^{77}Se NMR spectrum ($CDCl_3$, 76 MHz) of **4**.

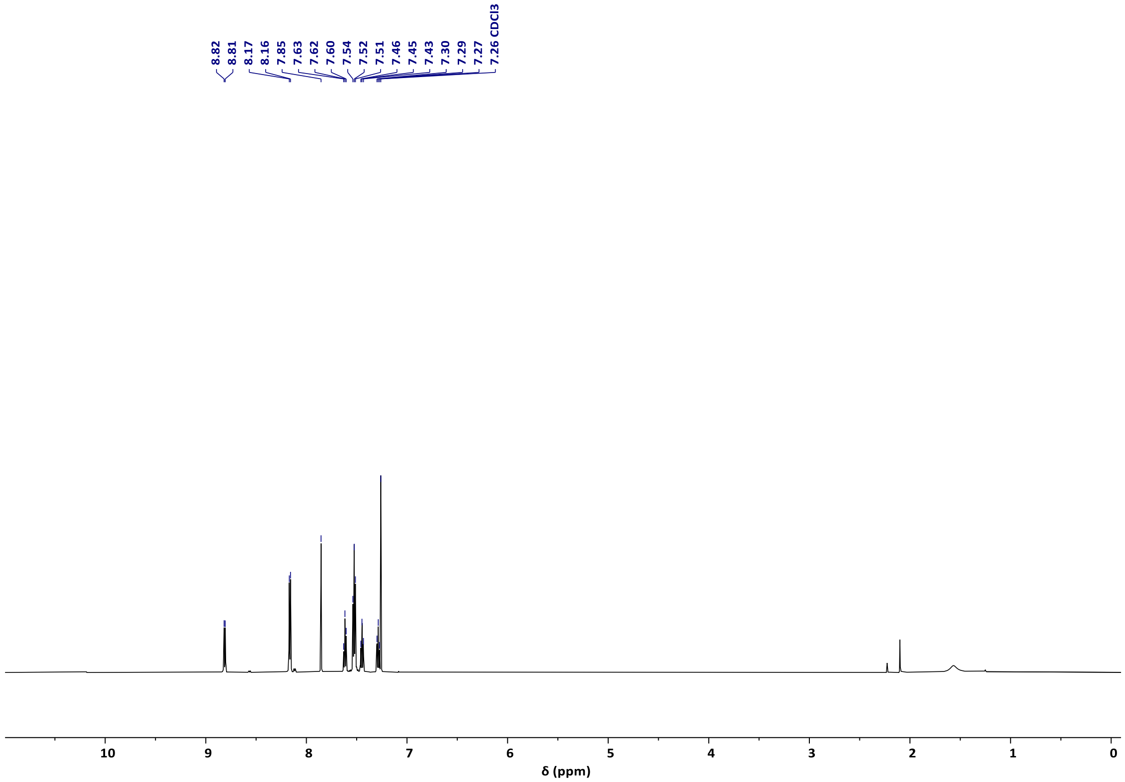

Figure S20. ^{1}H NMR spectrum ($CDCl_3$, 600 MHz) of **5**.

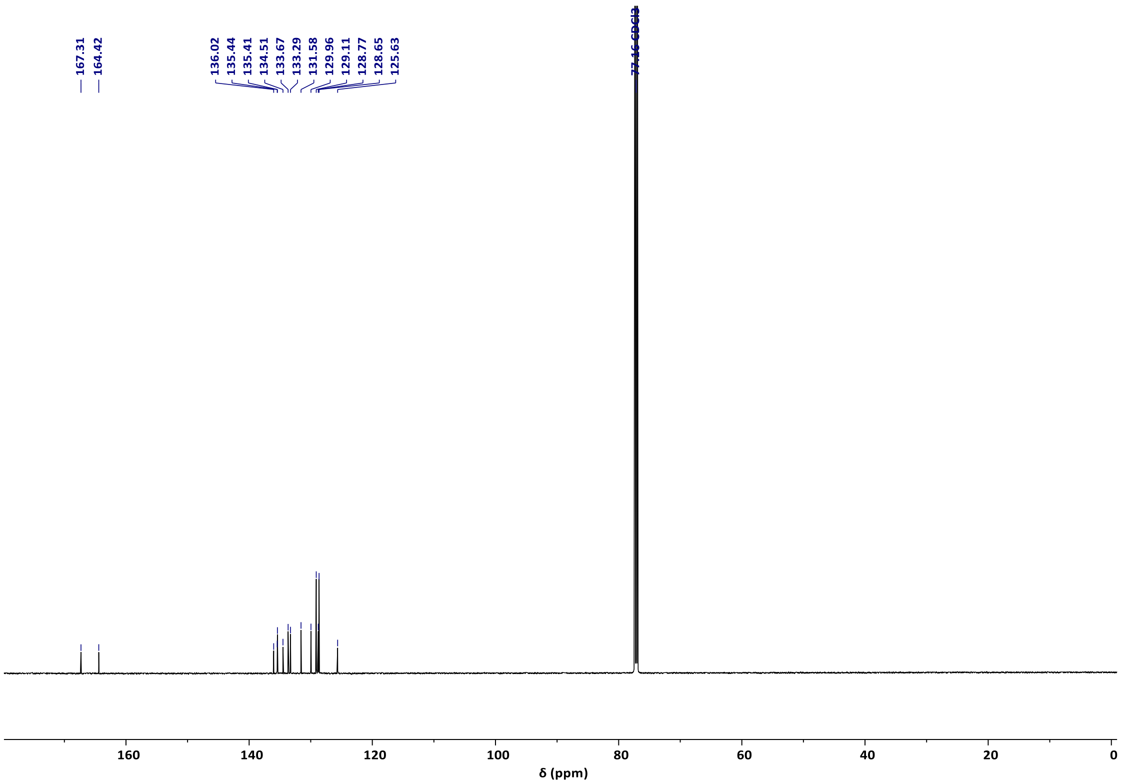

Figure S21. ^{13}C NMR spectrum ($CDCl_3$, 151 MHz) of **5**.

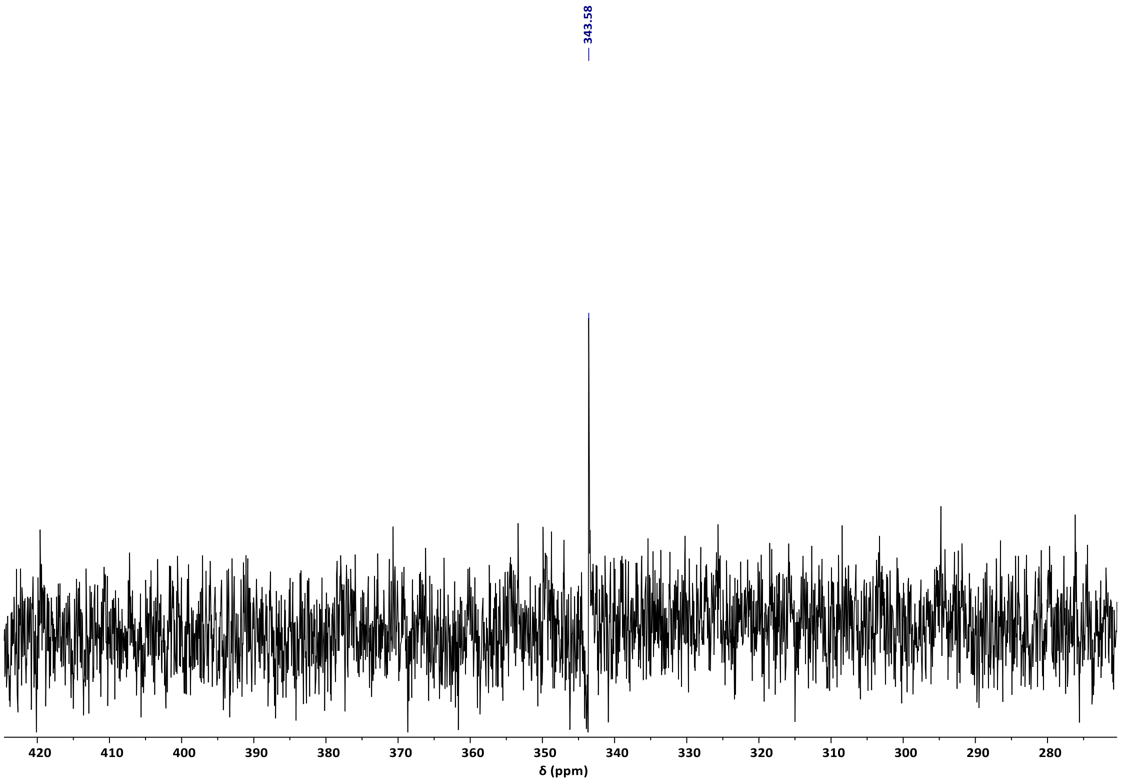

Figure S22. ^{77}Se NMR spectrum ($CDCl_3$, 114 MHz) of **5**.

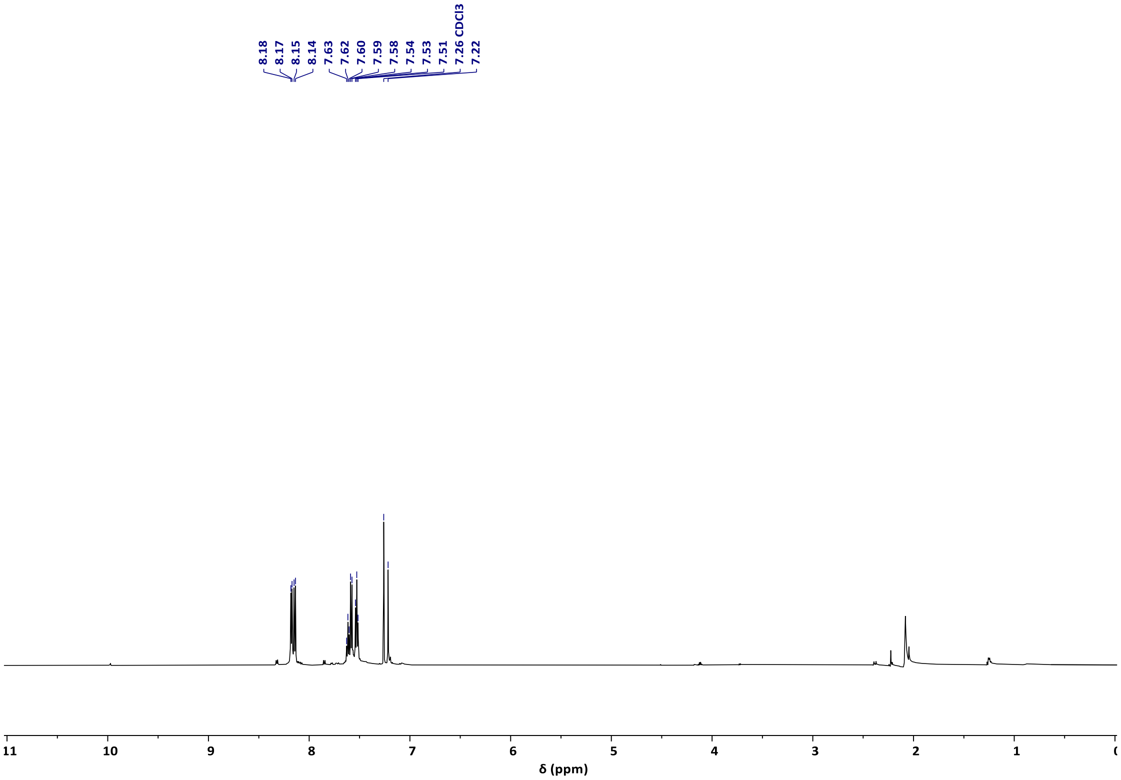

Figure S23. ^{1}H NMR spectrum ($CDCl_3$, 400 MHz) of **6**.

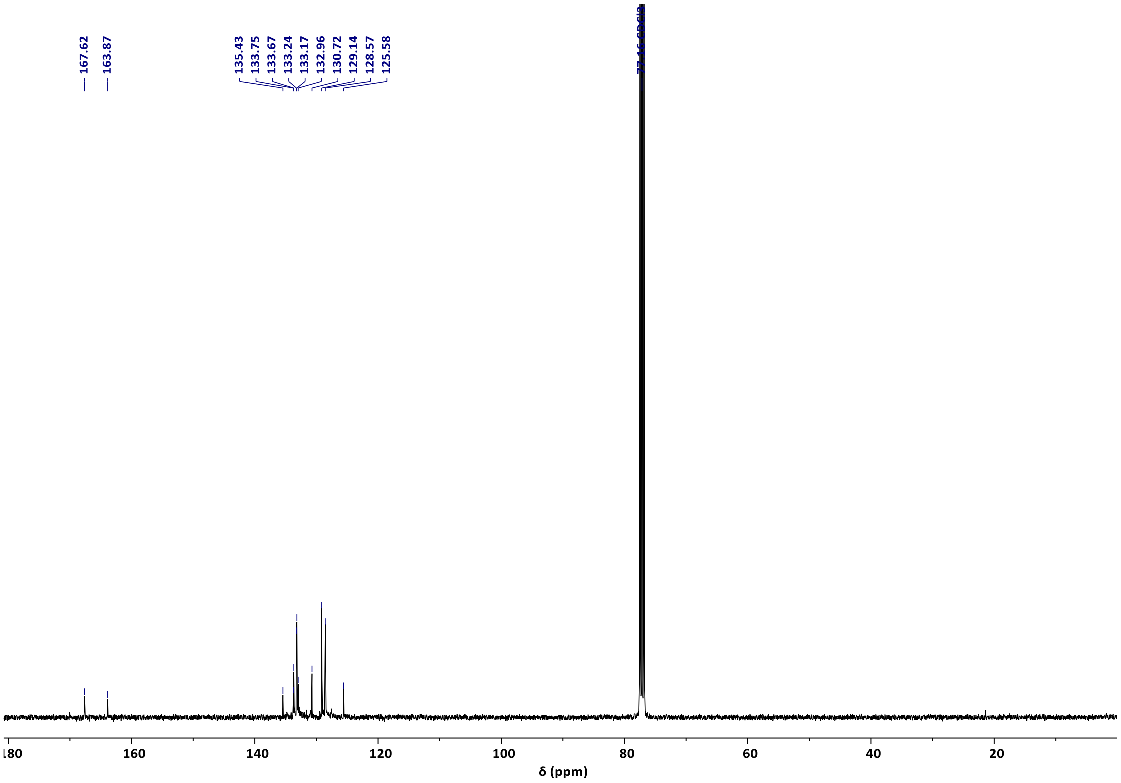

Figure S24. ^{13}C NMR spectrum ($CDCl_3$, 101 MHz) of **6**.

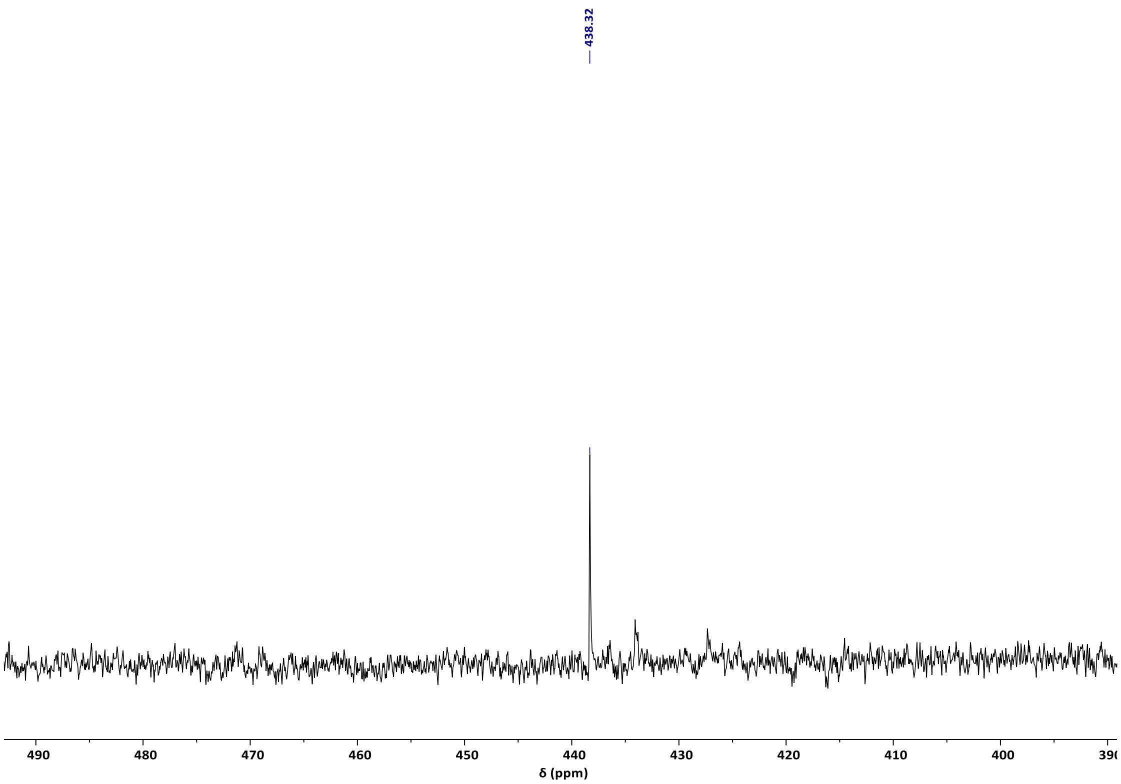

Figure S25. ^{77}Se NMR spectrum ($CDCl_3$, 76 MHz) of **6**.

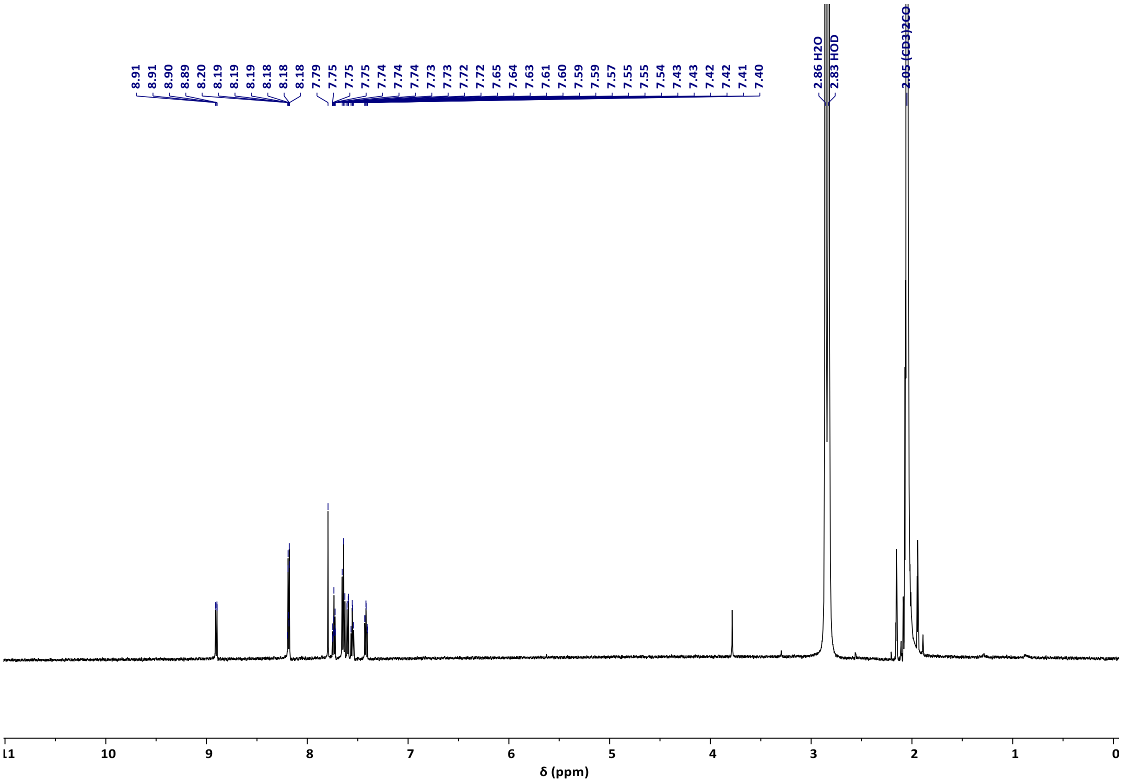

Figure S26. ^{1}H NMR spectrum (acetone-d_6, 600 MHz) of **7**.

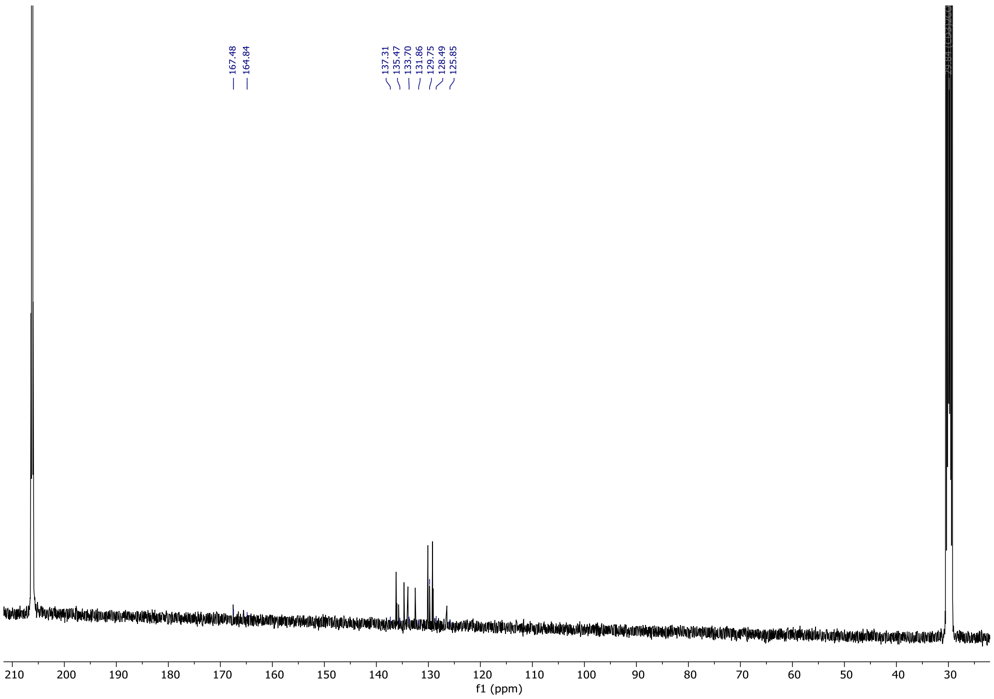

Figure S27. ^{13}C NMR spectrum (acetone-d_6, 151 MHz) of **7**.

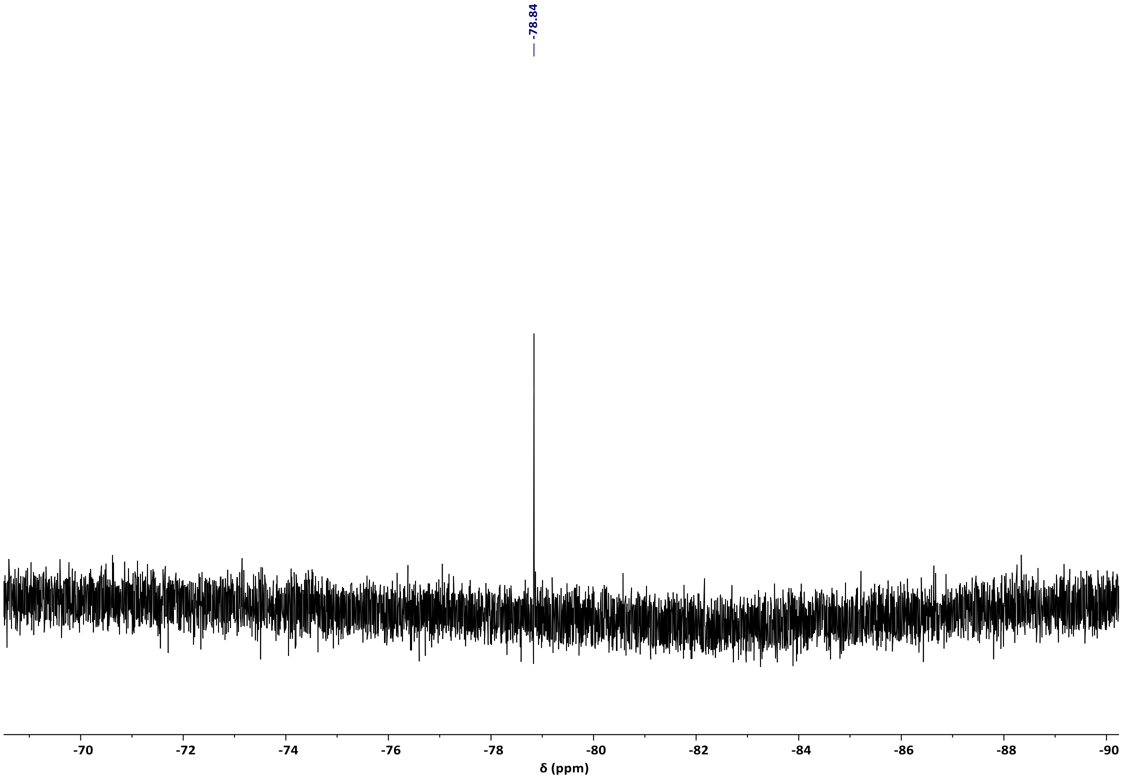

Figure S28. ^{19}F NMR spectrum (acetone-d_6, 565 MHz) of **7**.

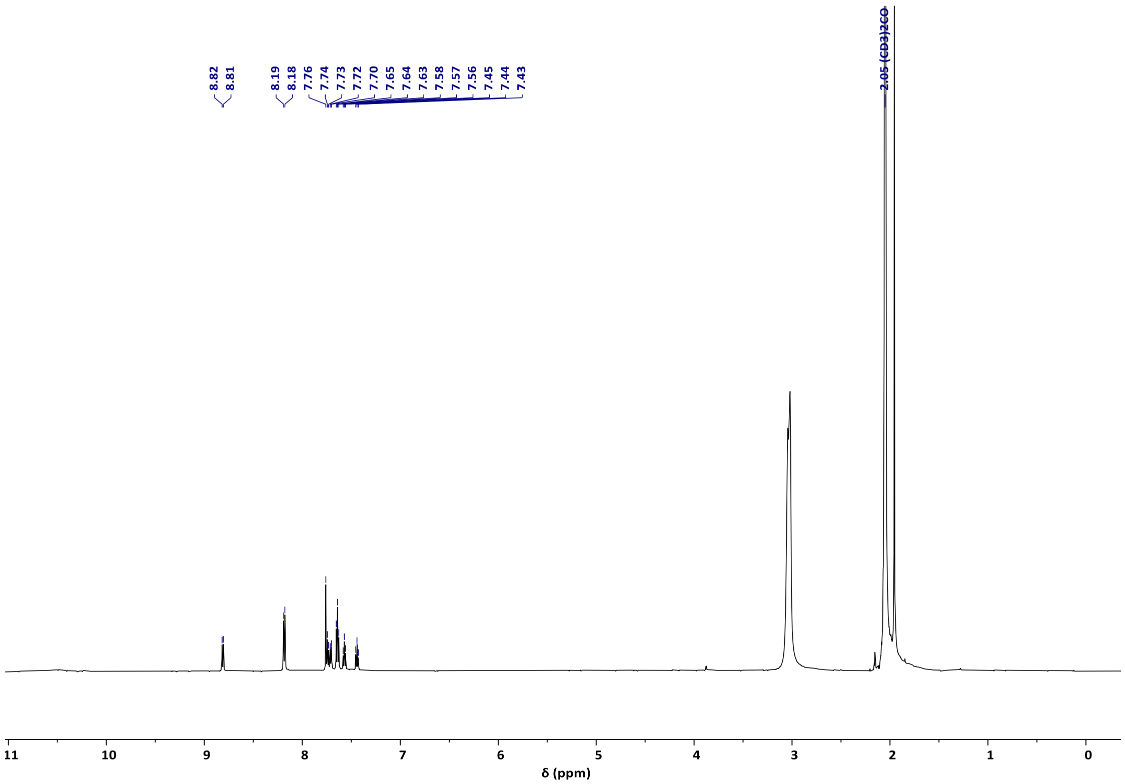

Figure S29. ^{1}H NMR spectrum (acetone-d_6, 600 MHz) of **9**.

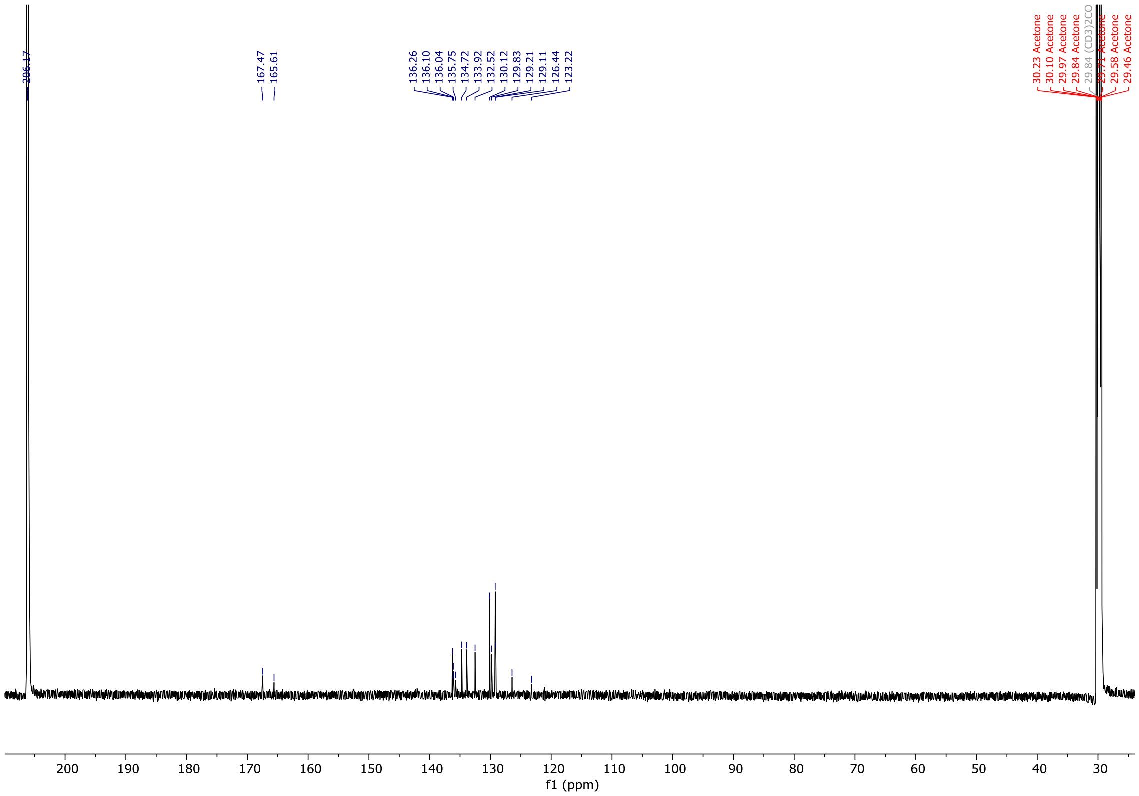

Figure S30. ^{13}C NMR spectrum (acetone-d_6, 151 MHz) of **9**.

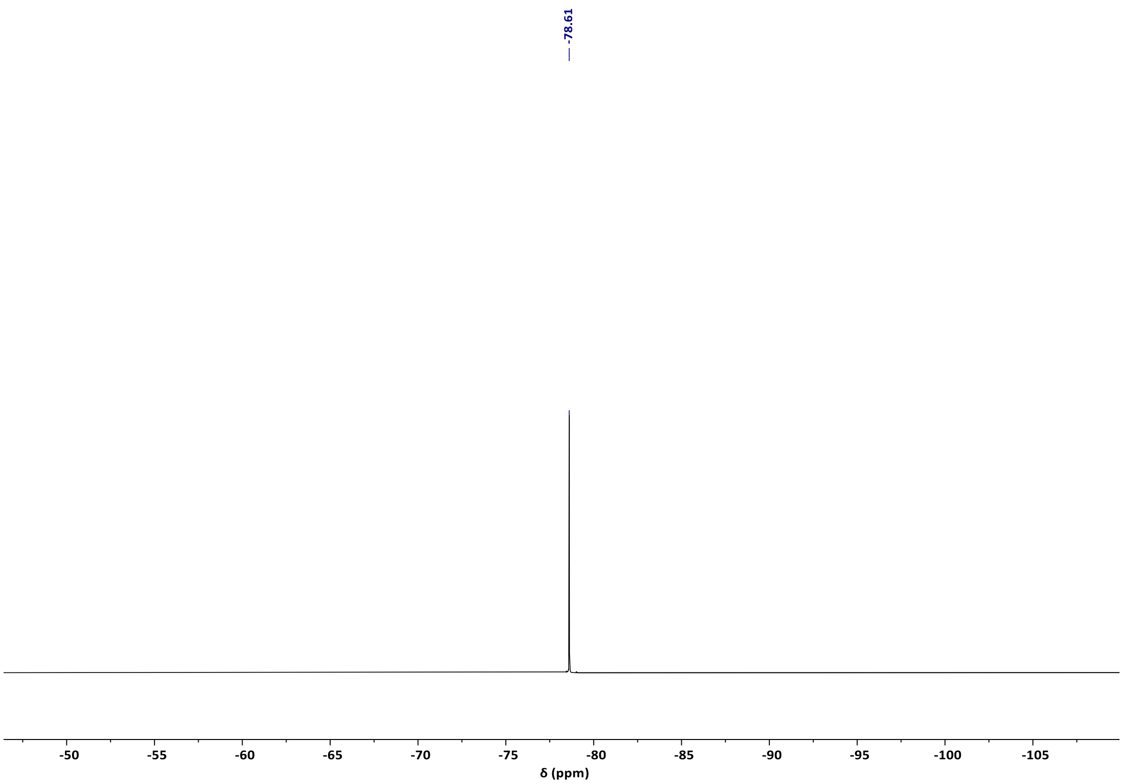

Figure S31. ^{19}F NMR spectrum (acetone-d_6, 565 MHz) of **9**.

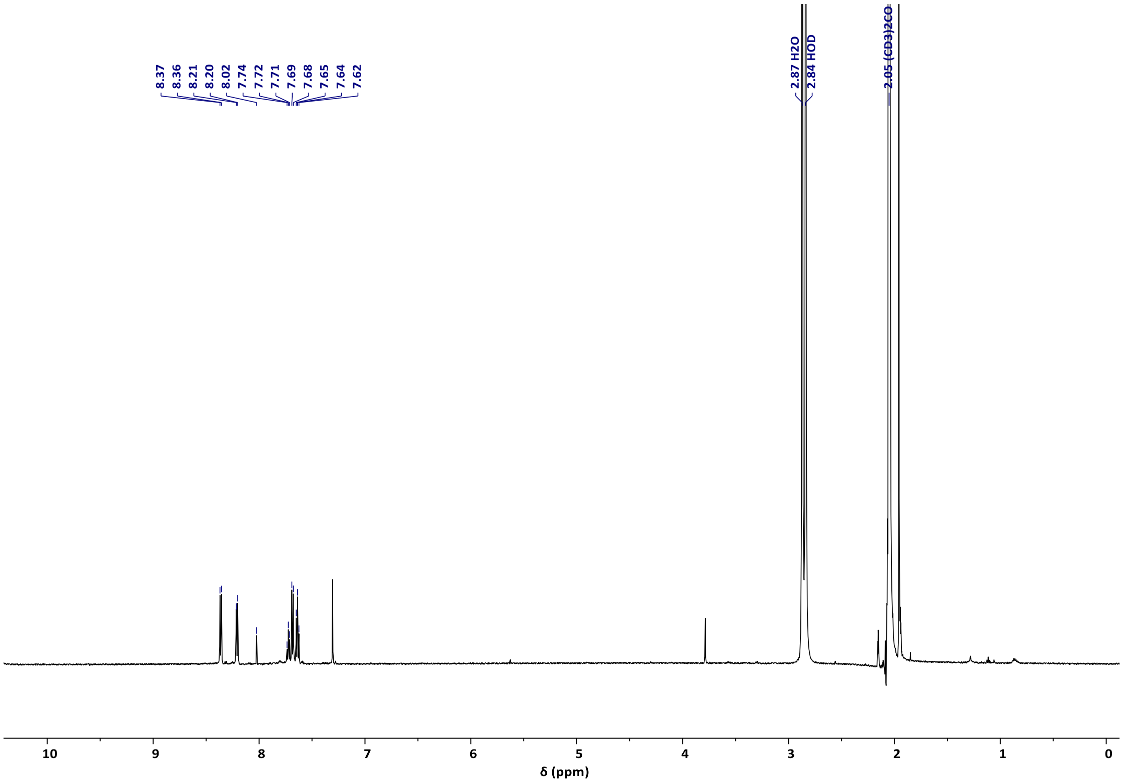

Figure S32. ^{1}H NMR spectrum (acetone-d_6, 600 MHz) of **8**.

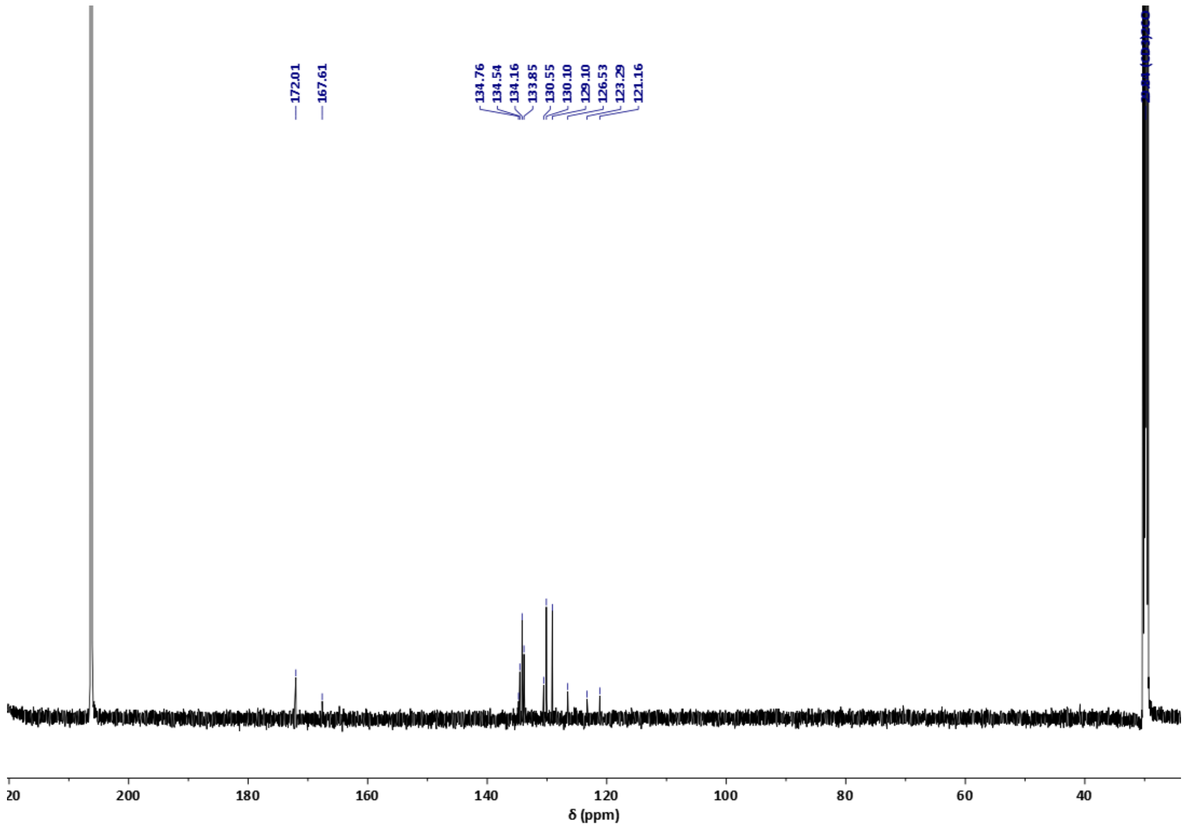

Figure S33. ^{13}C NMR spectrum (acetone-d_6, 151 MHz) of **8**.

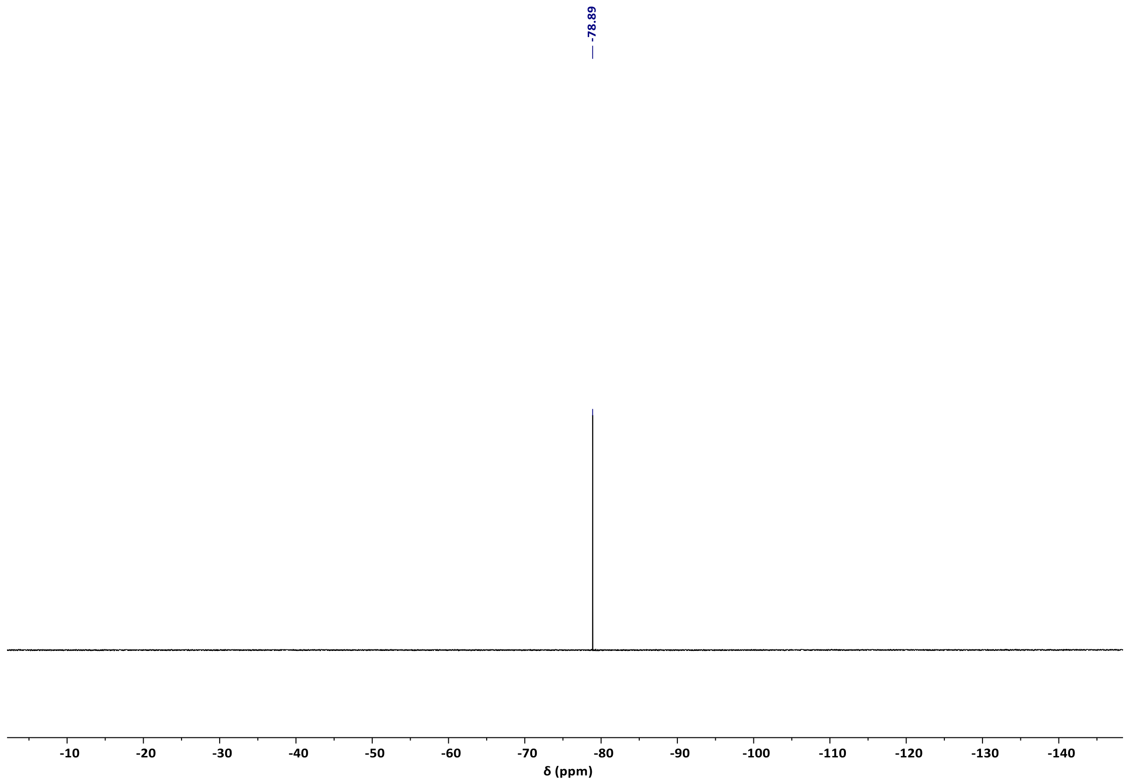

Figure S34. ^{19}F NMR spectrum (acetone-d_6, 565 MHz) of **8**.

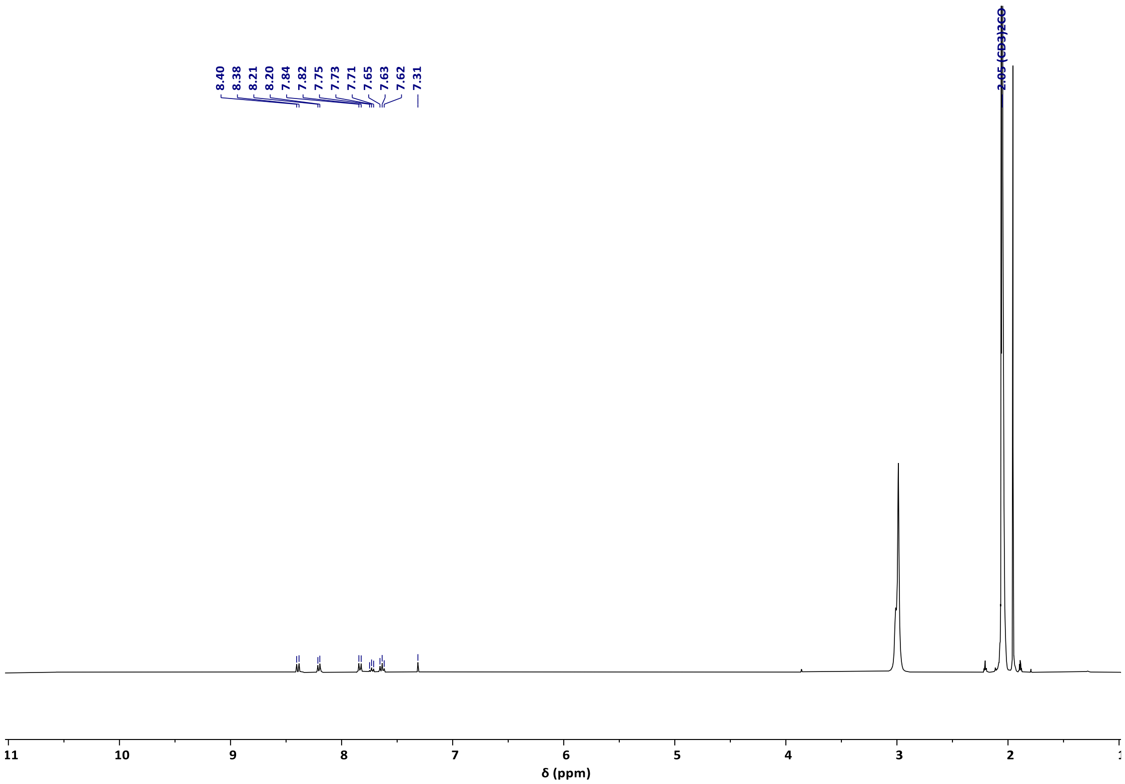

Figure S35. ^{1}H NMR spectrum (acetone-d_6, 400 MHz) of **10**.

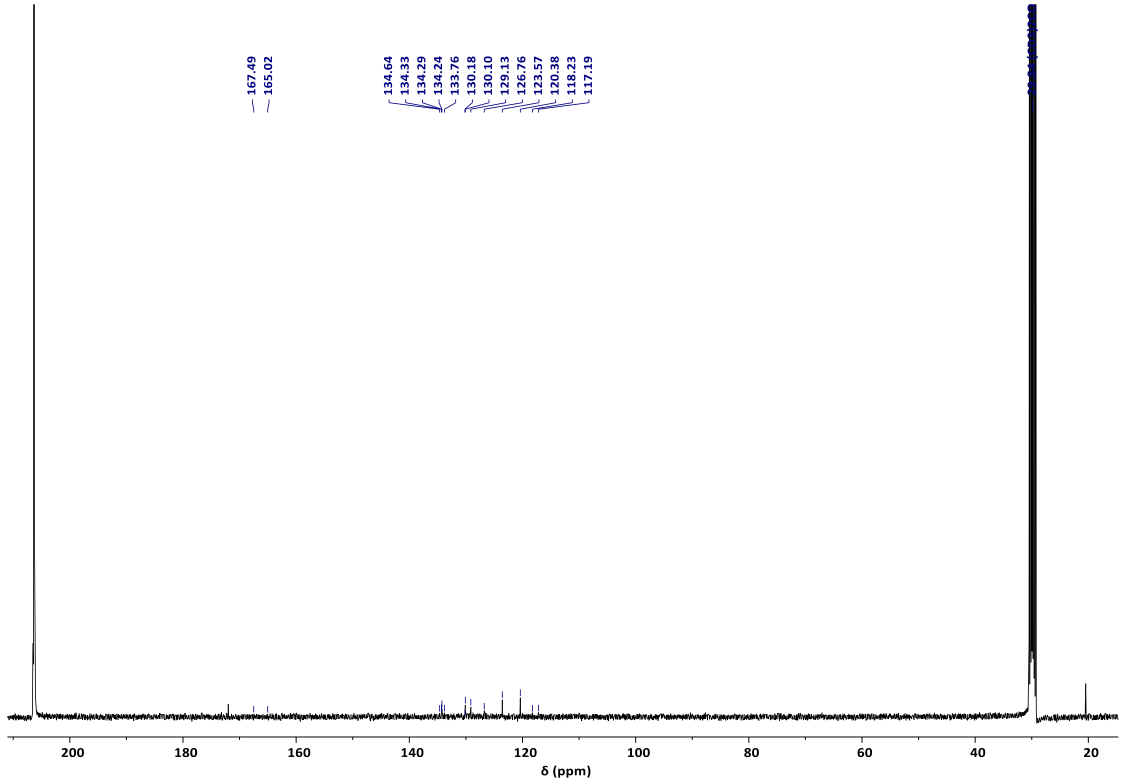

Figure S36. ^{13}C NMR spectrum (acetone-d_6, 101 MHz) of **10**.

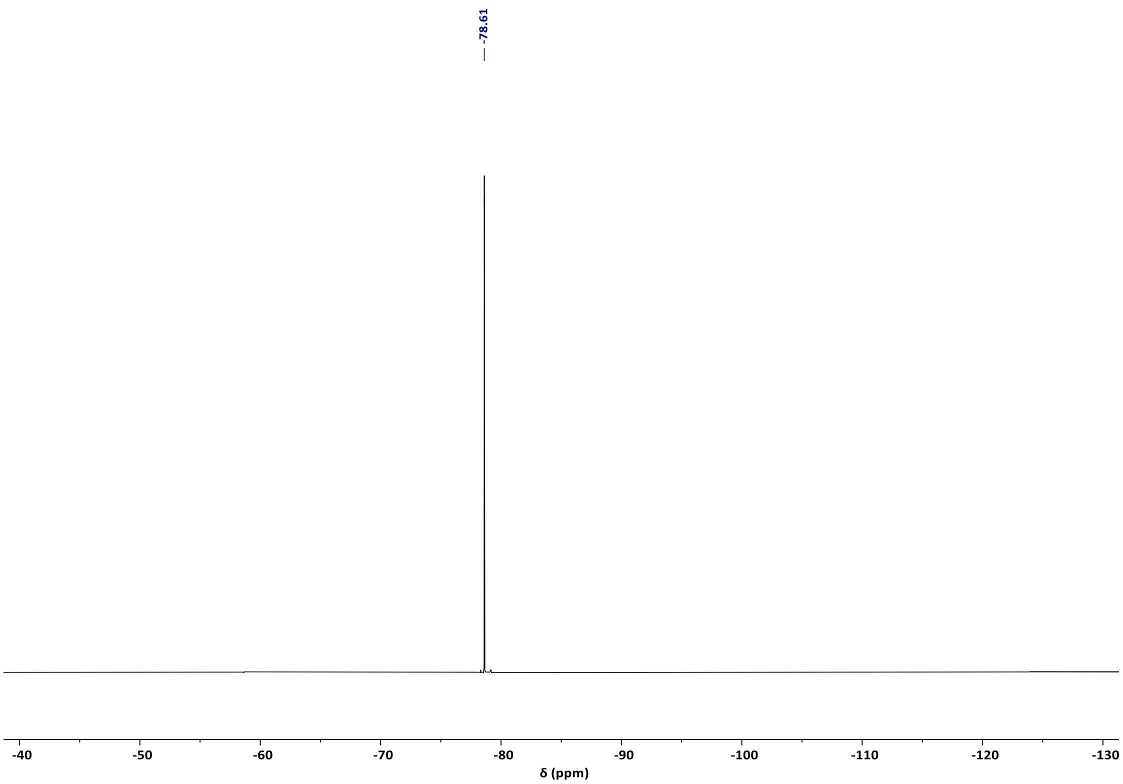

Figure S37. ^{19}F NMR spectrum (acetone-d_6, 376 MHz) of **10**.

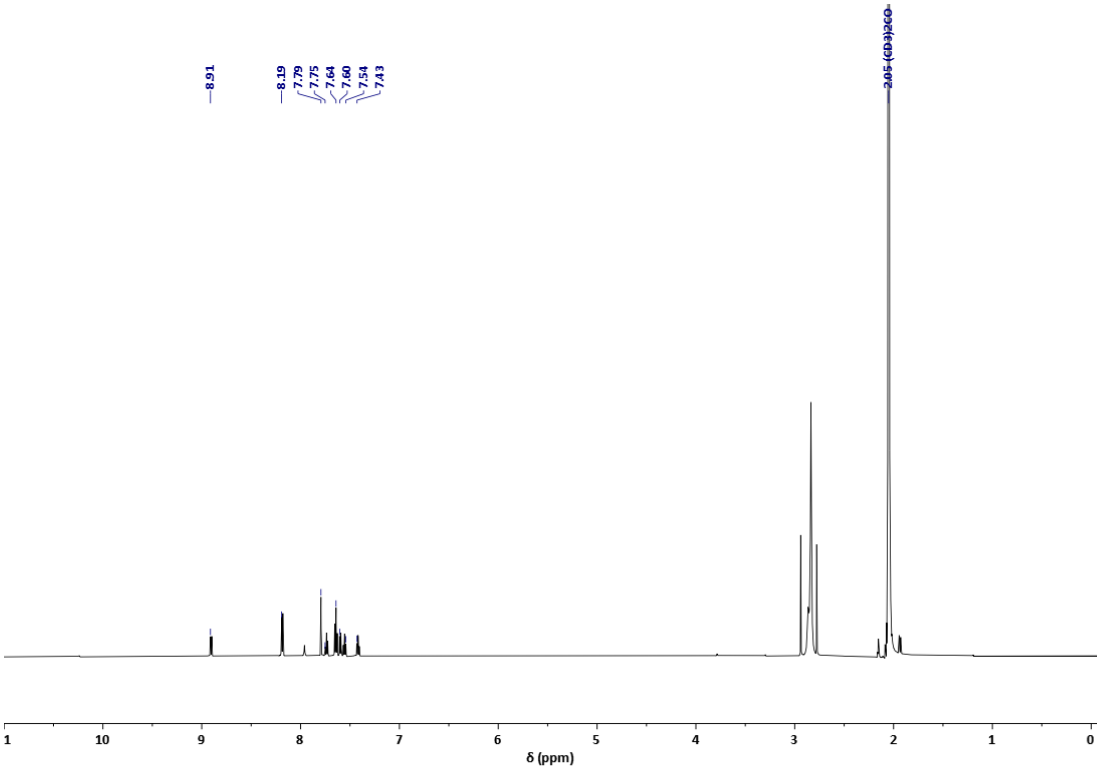

Figure S38. ^{1}H NMR spectrum (acetone-d_6, 600 MHz) of **11**.

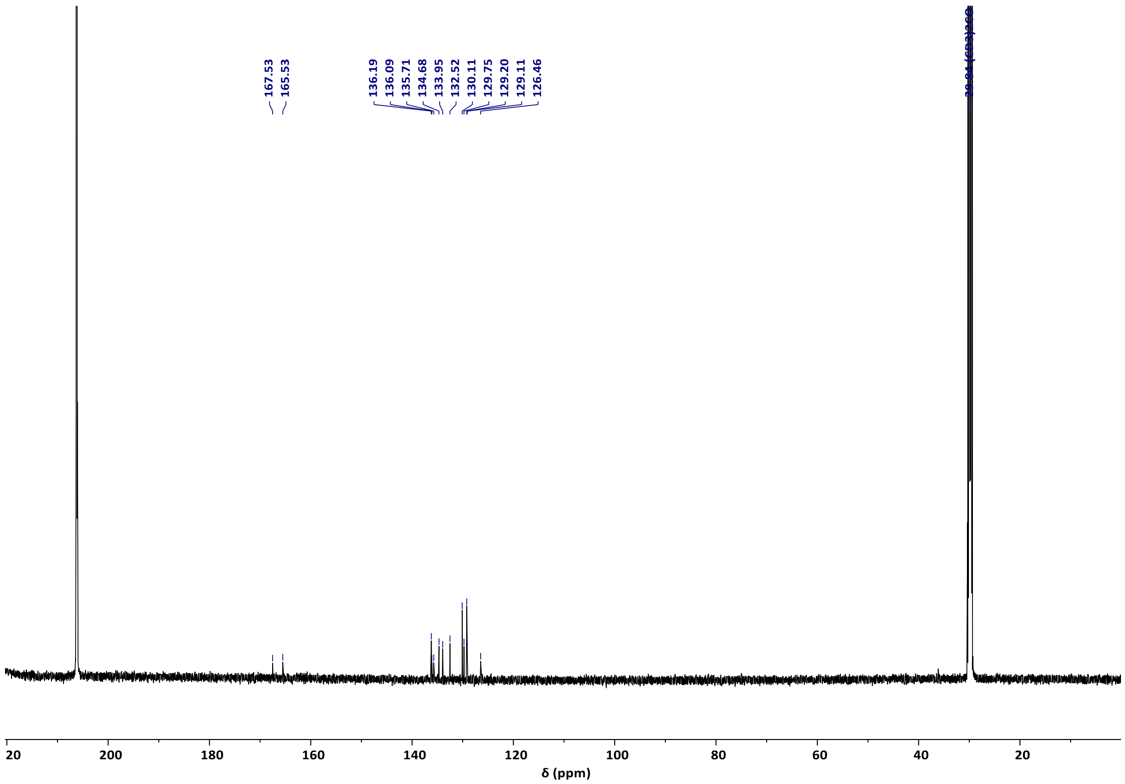

Figure S39. ^{13}C NMR spectrum (acetone-d_6, 151 MHz) of **11**.

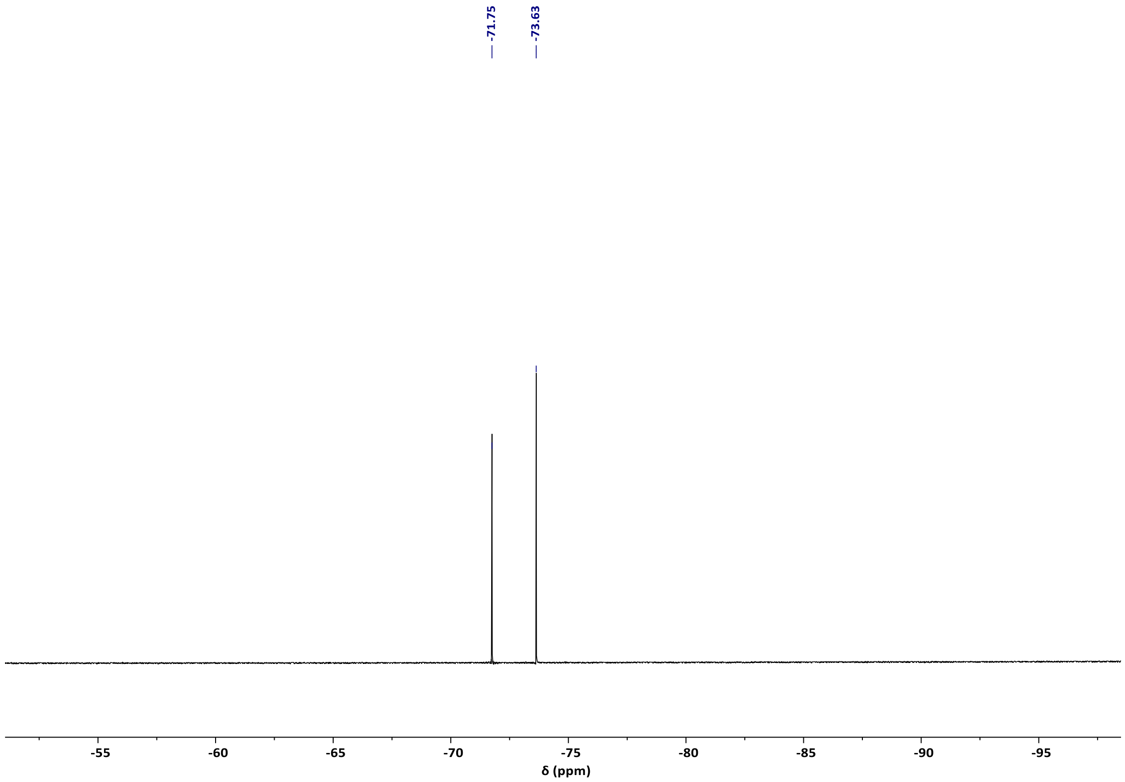

Figure S40. ^{19}F NMR spectrum (acetone-d_6, 376 MHz) of **11**.

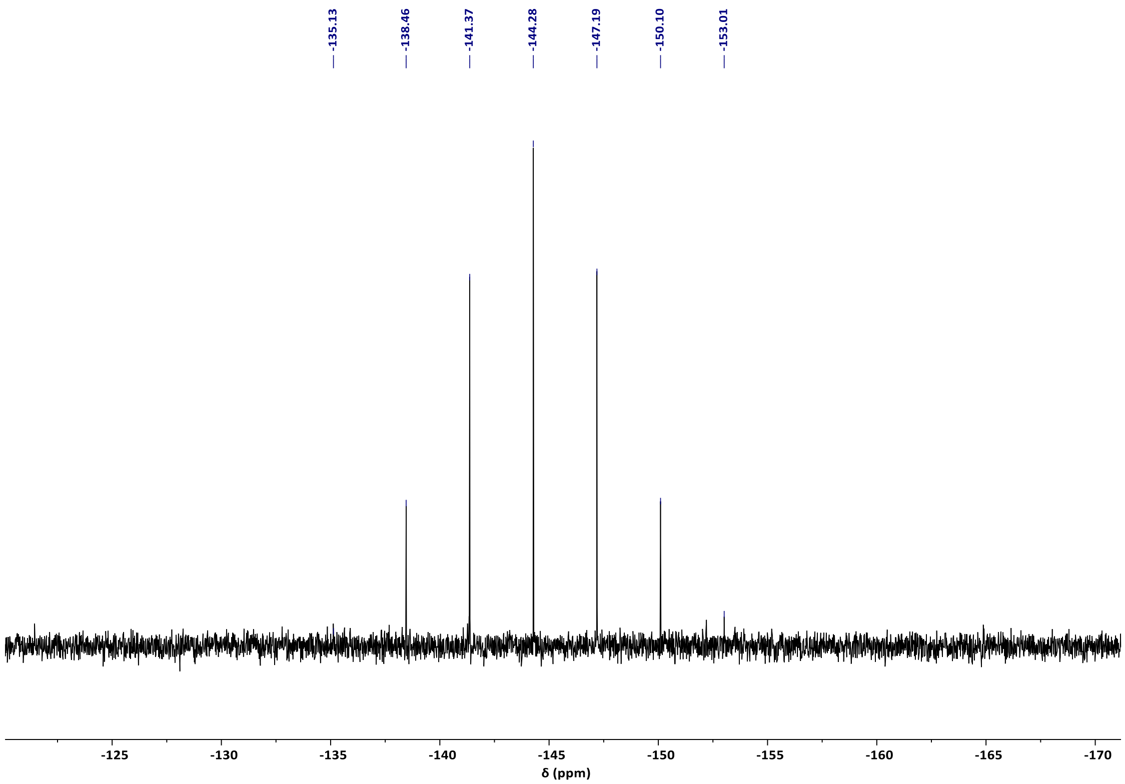

Figure S41. ^{31}P NMR spectrum (acetone-d_6, 243 MHz) of **11**.

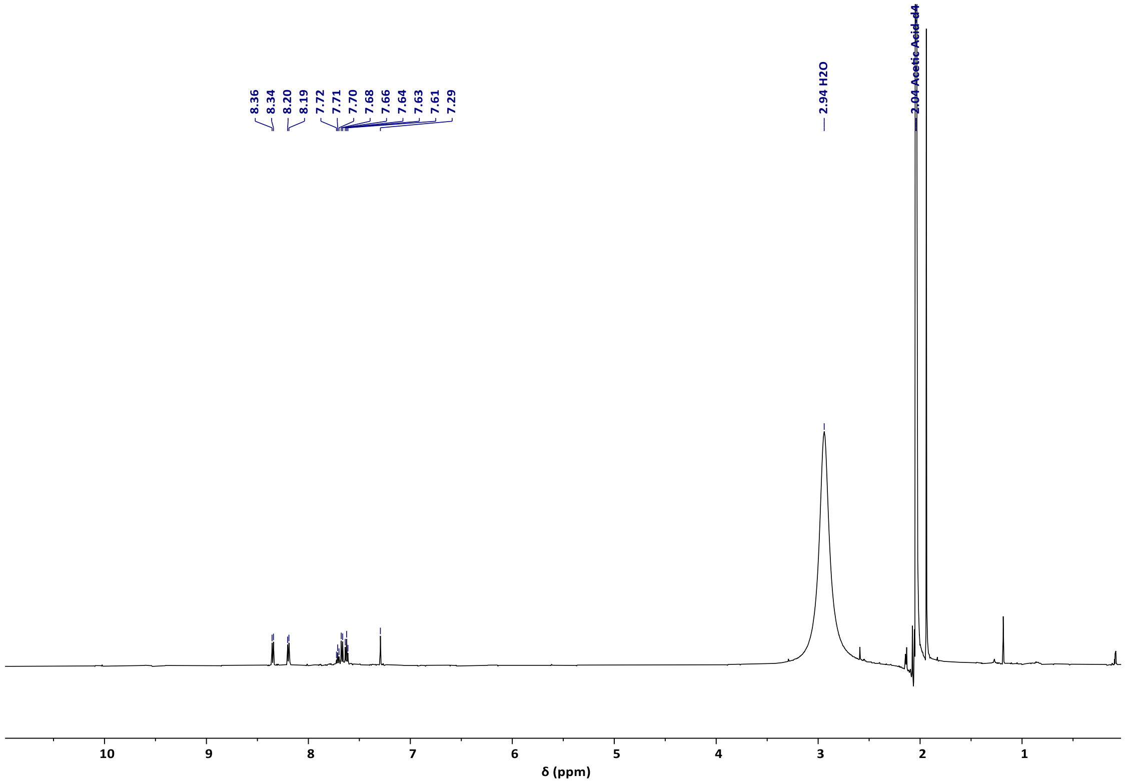

Figure S42. ^{1}H NMR spectrum (acetone-d_6, 600 MHz) of **12**.

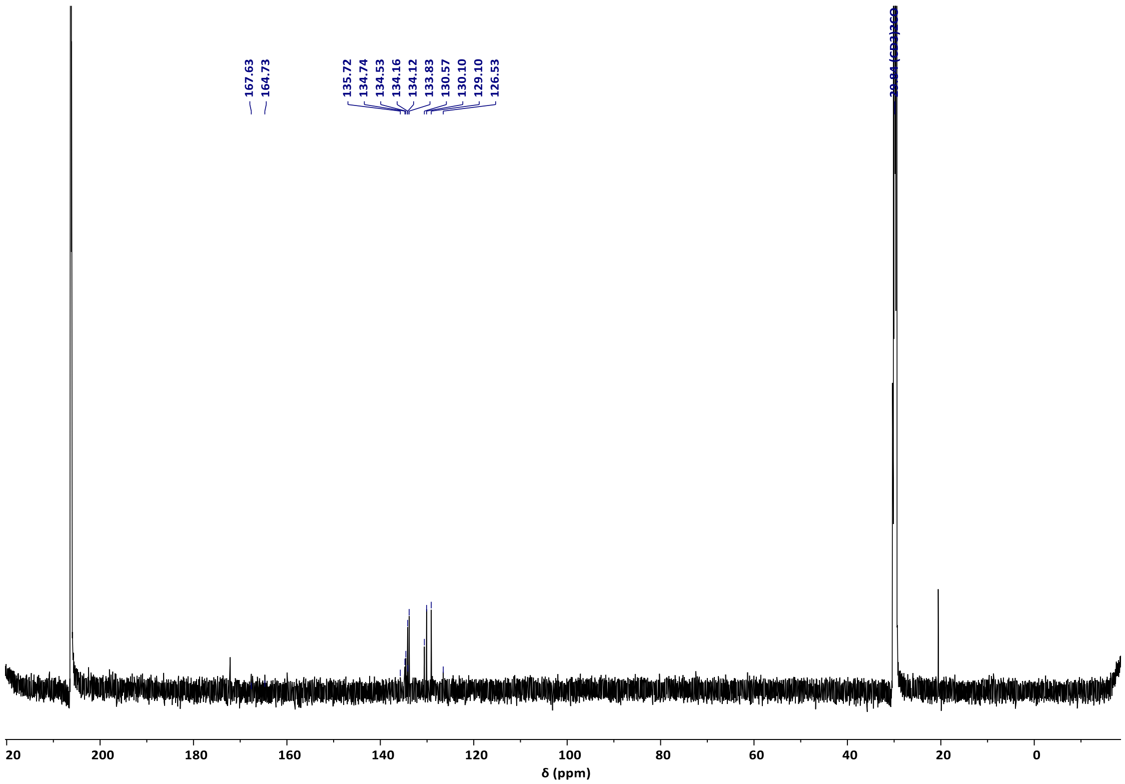

Figure S43. ^{13}C NMR spectrum (acetone-d_6, 151 MHz) of **12**.

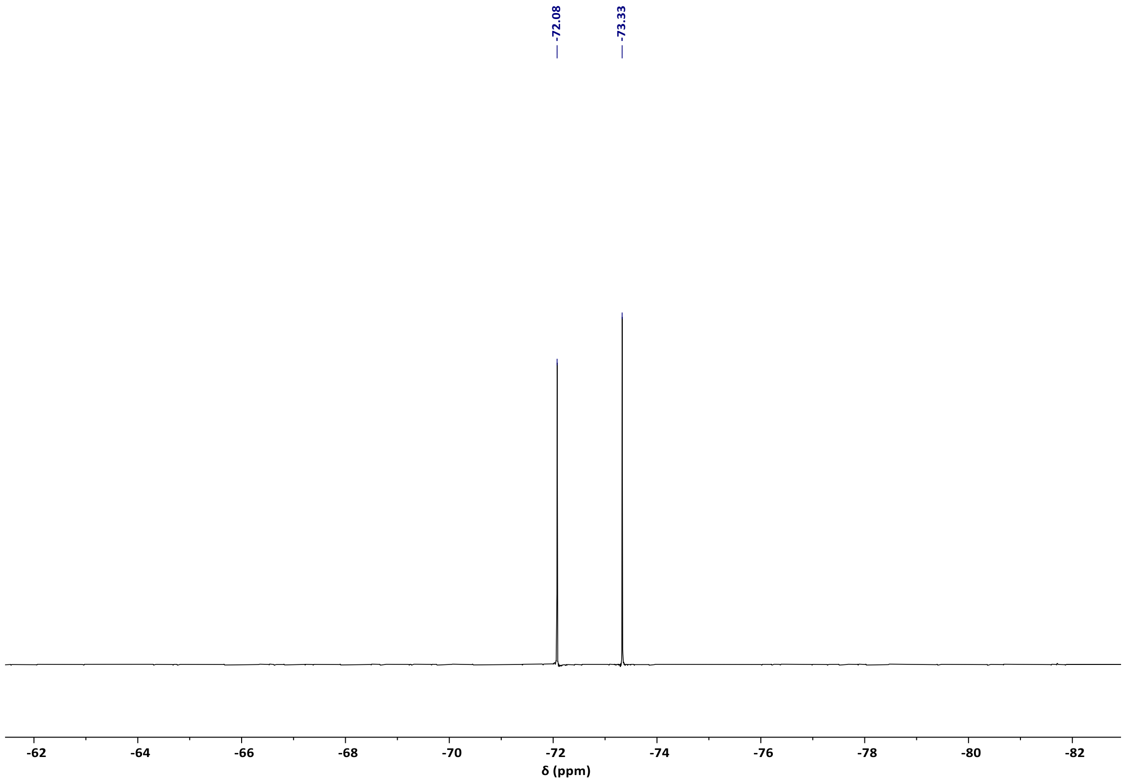

Figure S44. ^{19}F NMR spectrum (acetone-d_6, 565 MHz) of **12**.

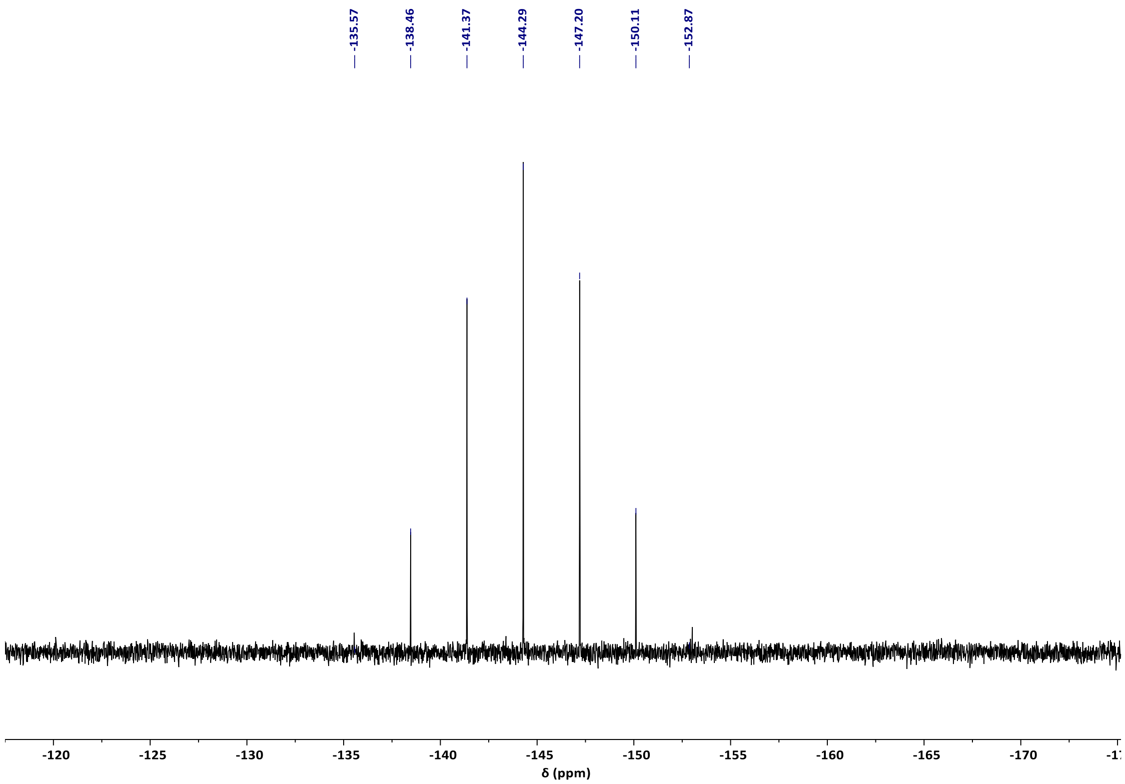

Figure S45. ^{31}P NMR spectrum (acetone-d_6, 243 MHz) of **12**.

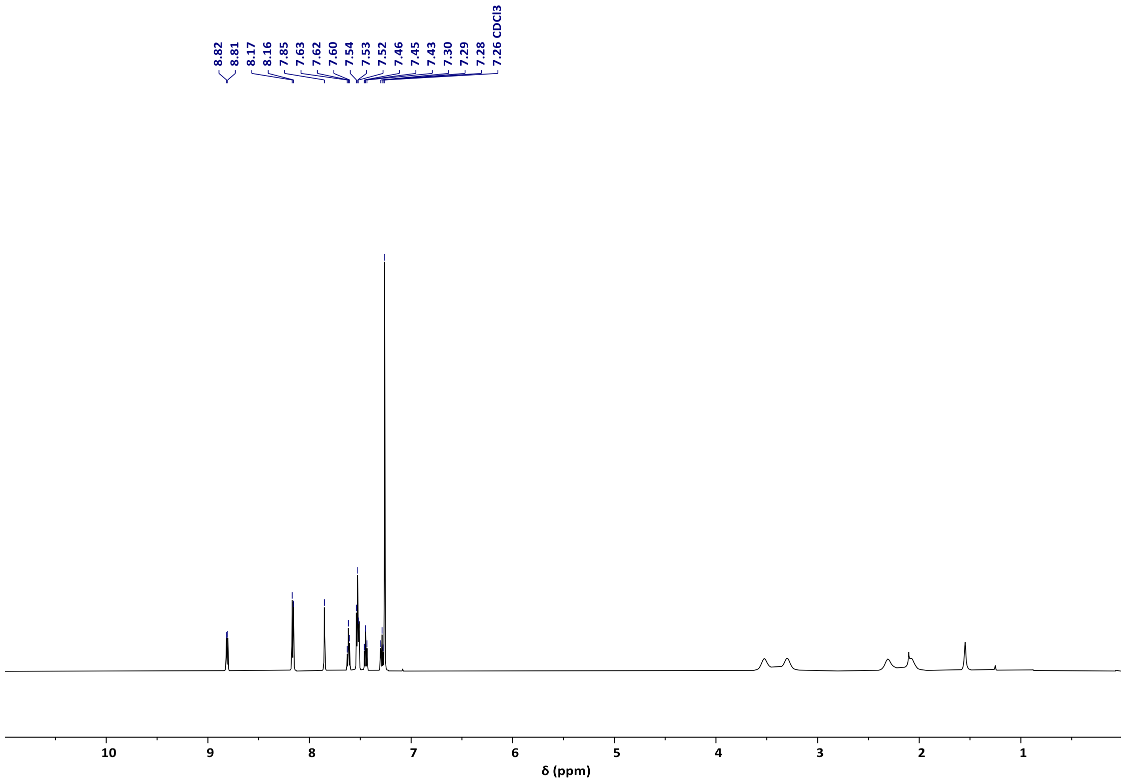

Figure S46. ^{1}H NMR spectrum ($CDCl_3$, 600 MHz) of **13**.

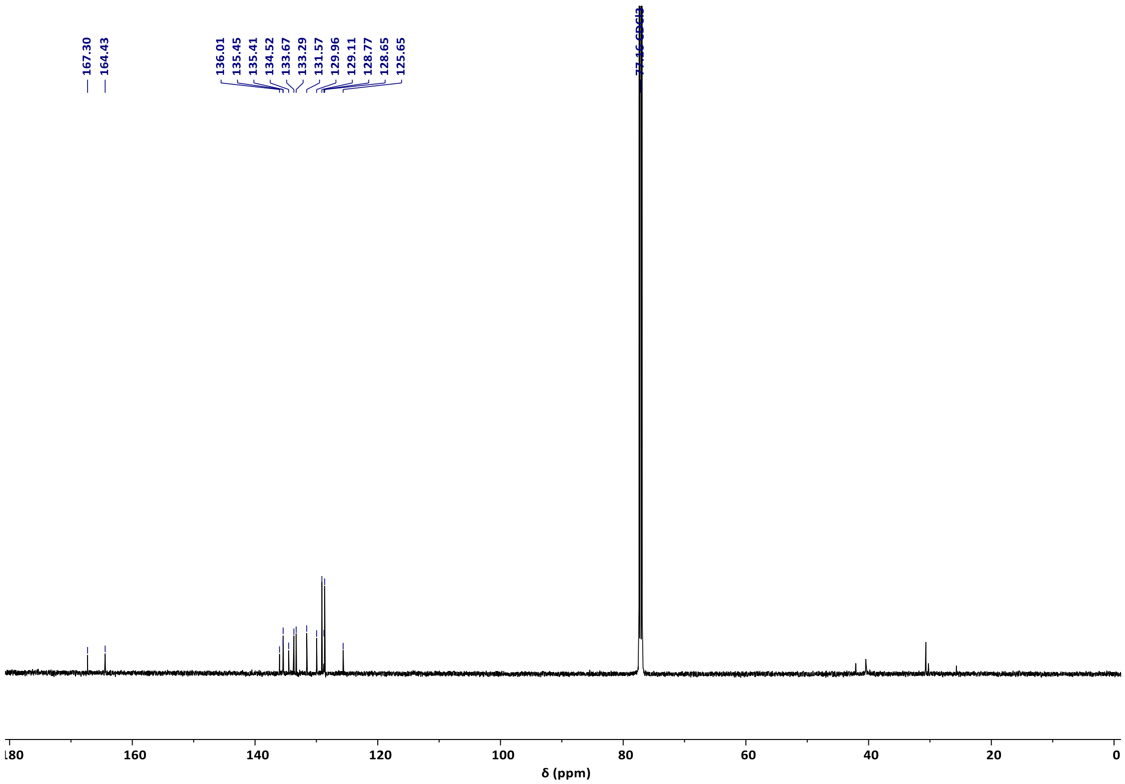

Figure S47. ^{13}C NMR spectrum ($CDCl_3$, 151 MHz) of **13**.

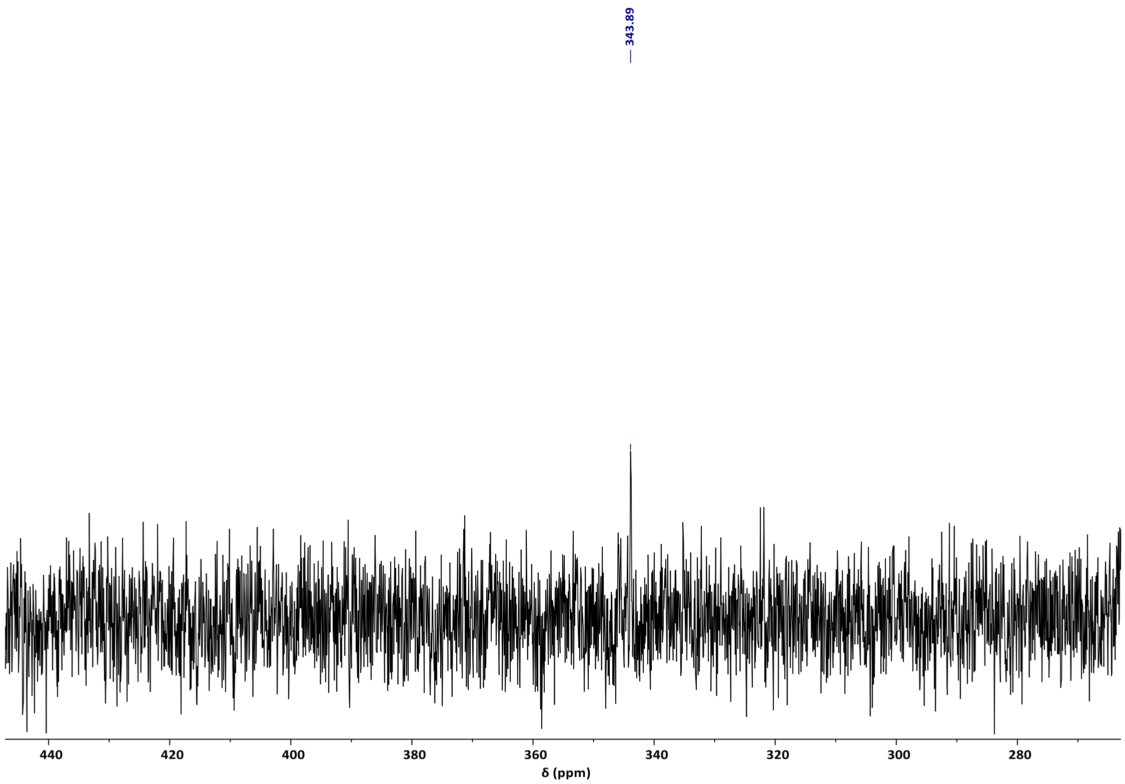

Figure S48. ^{77}Se NMR spectrum ($CDCl_3$, 114 MHz) of **13**.

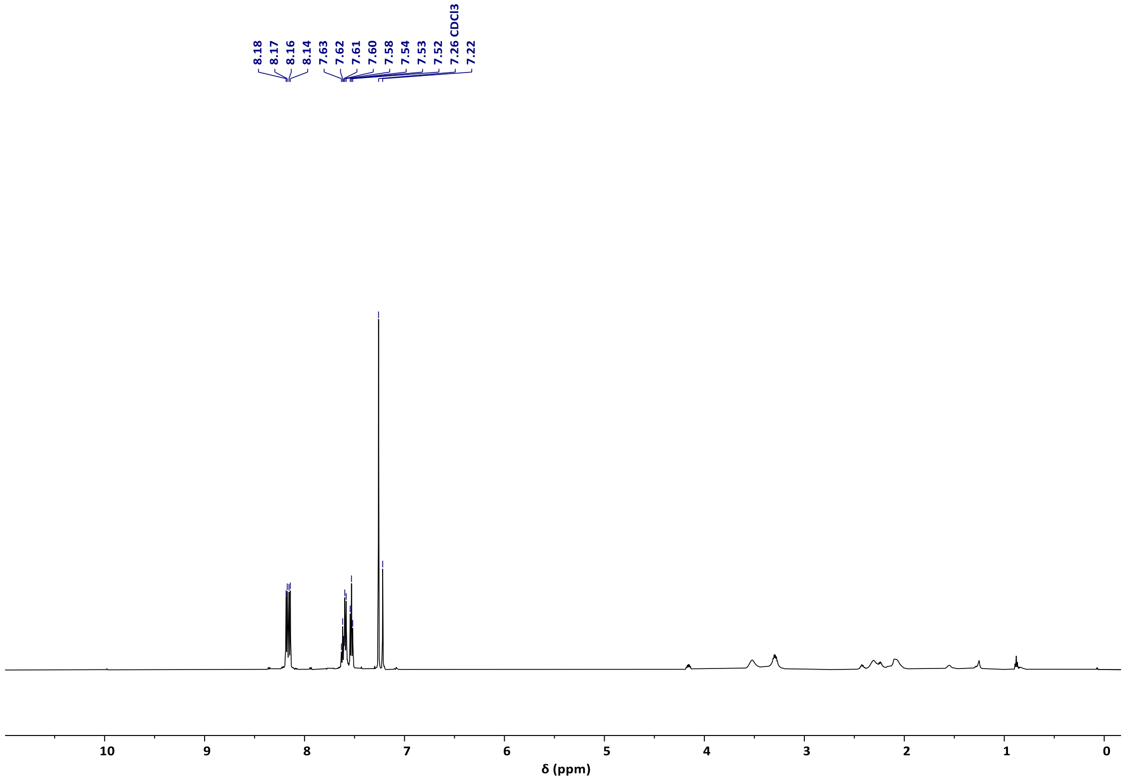

Figure S49. ^{1}H NMR spectrum ($CDCl_3$, 600 MHz) of **14**.

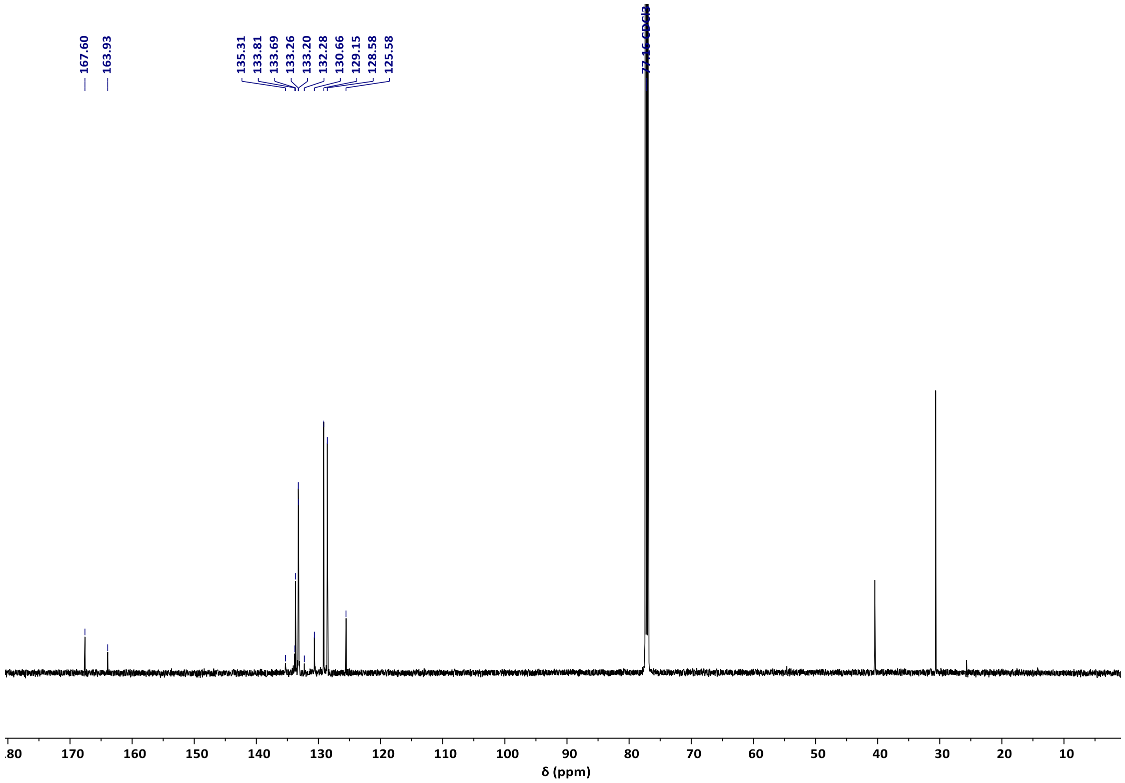

Figure S50. ^{13}C NMR spectrum ($CDCl_3$, 151 MHz) of **14**.

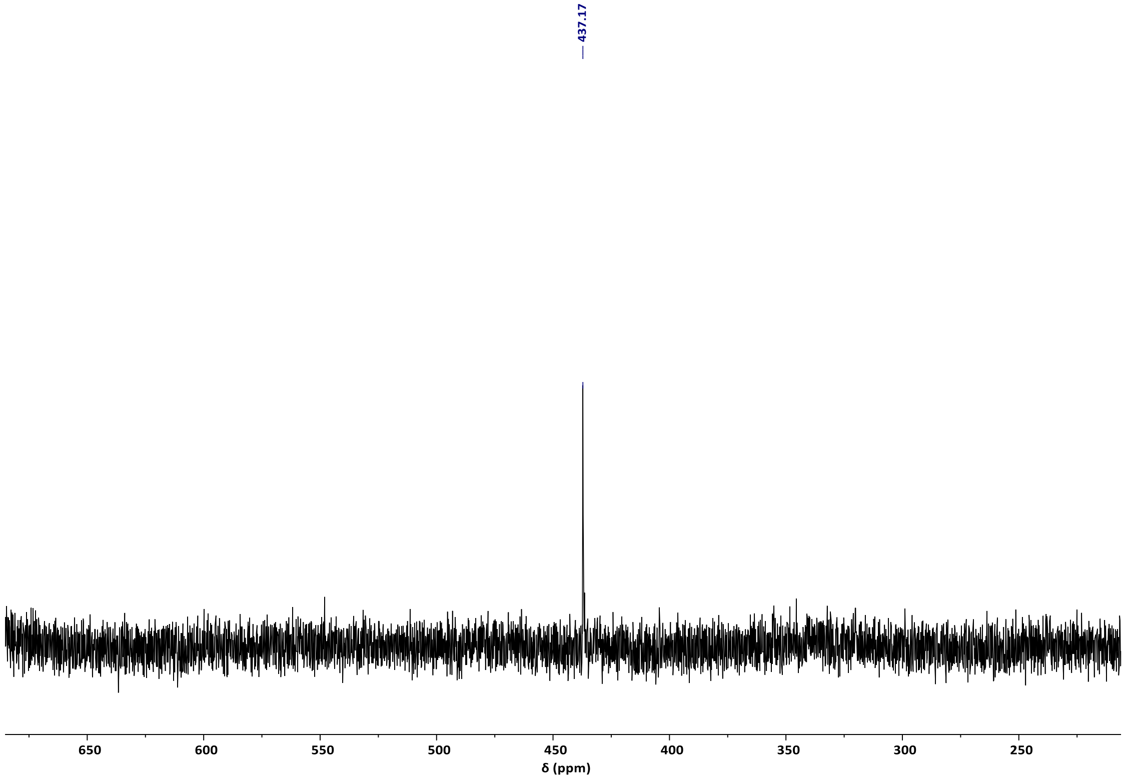

Figure S51. ^{77}Se NMR spectrum ($CDCl_3$, 114 MHz) of **14**.

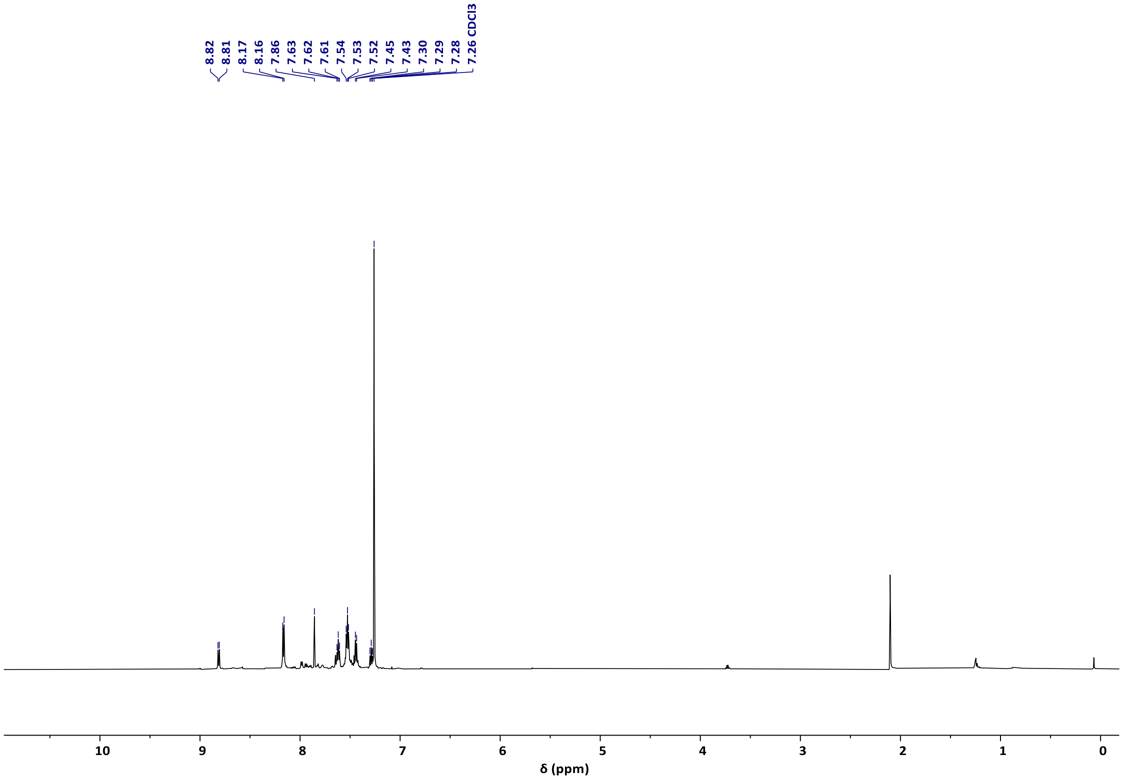

Figure S52. ^{1}H NMR spectrum ($CDCl_3$, 400 MHz) of **15**.

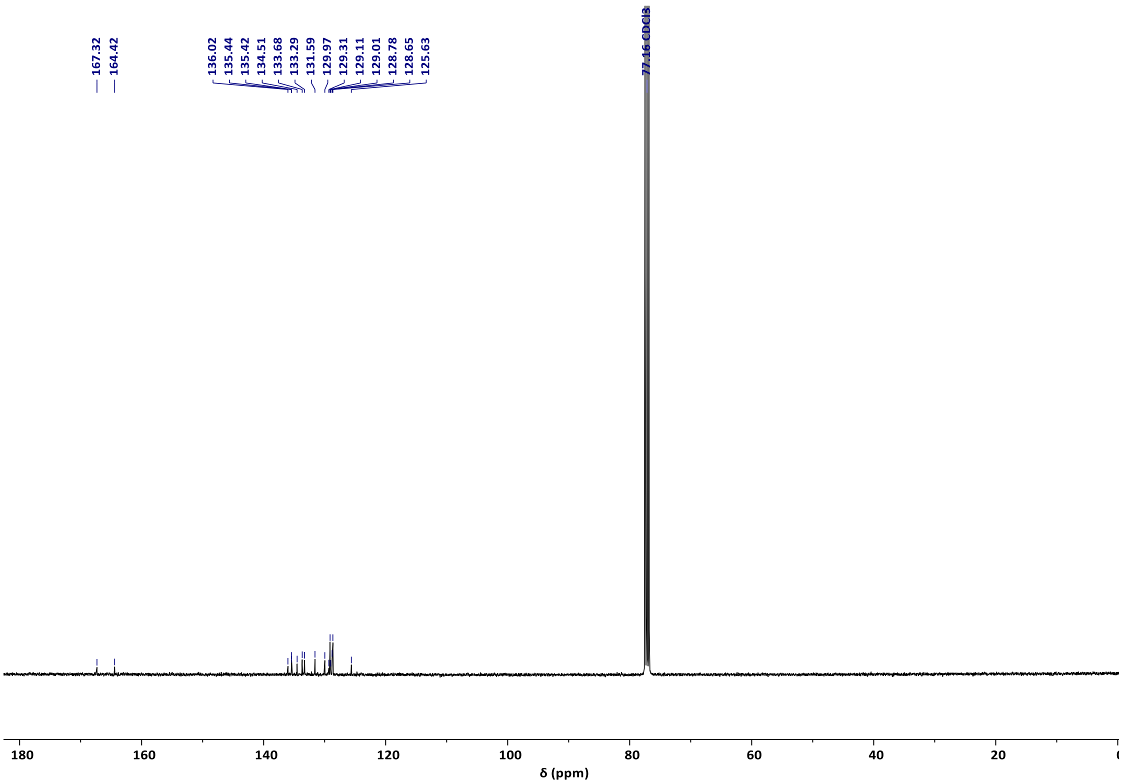

Figure S53. ^{13}C NMR spectrum ($CDCl_3$, 101 MHz) of **15**.

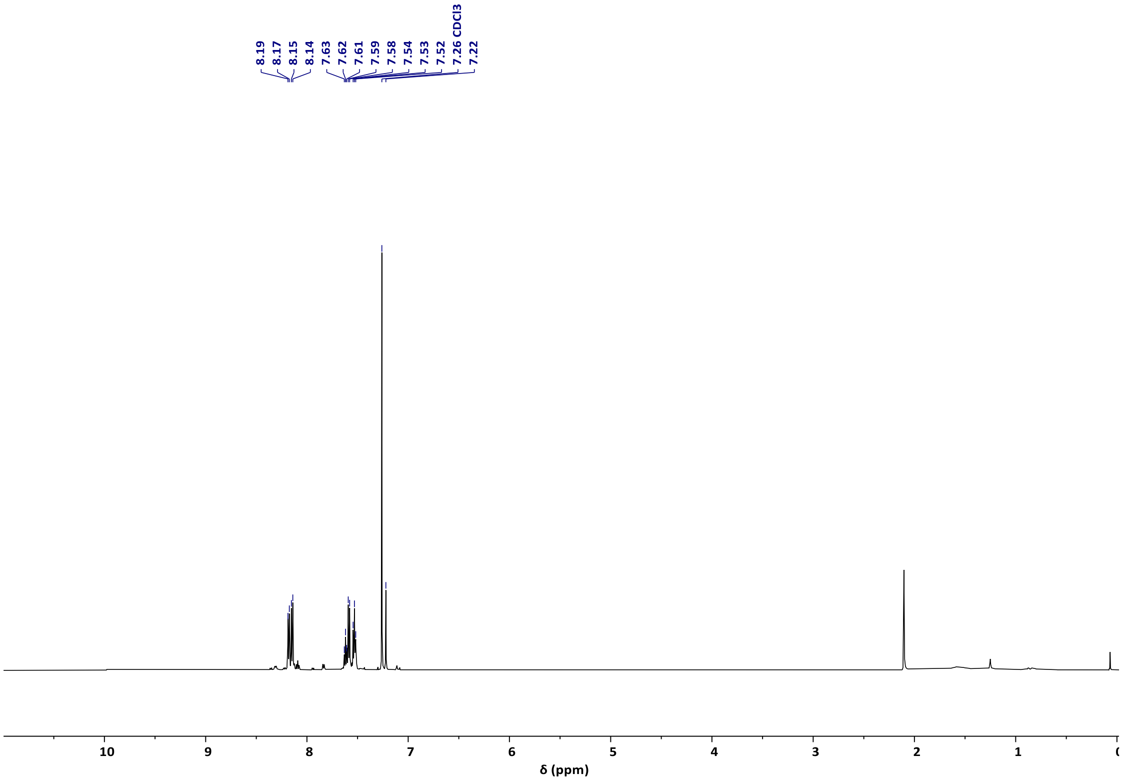

Figure S54. ^{1}H NMR spectrum ($CDCl_3$, 600 MHz) of **16**.

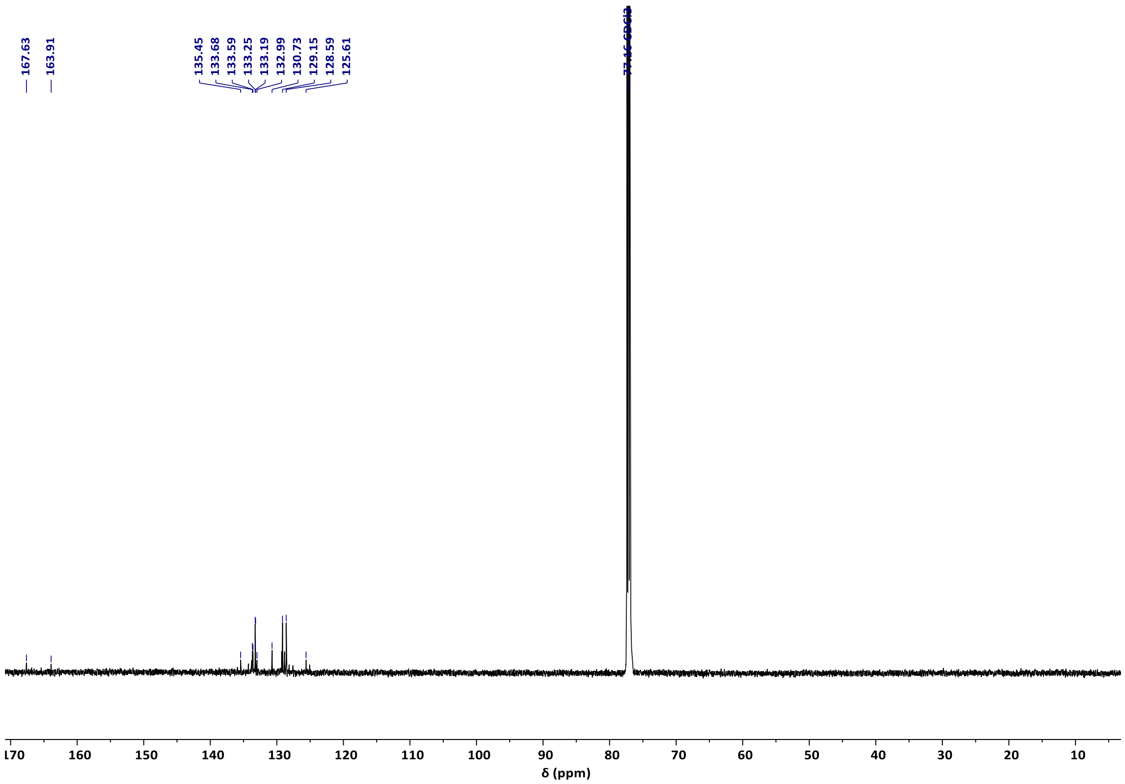

Figure S55. ^{13}C NMR spectrum ($CDCl_3$, 151 MHz) of **16**.

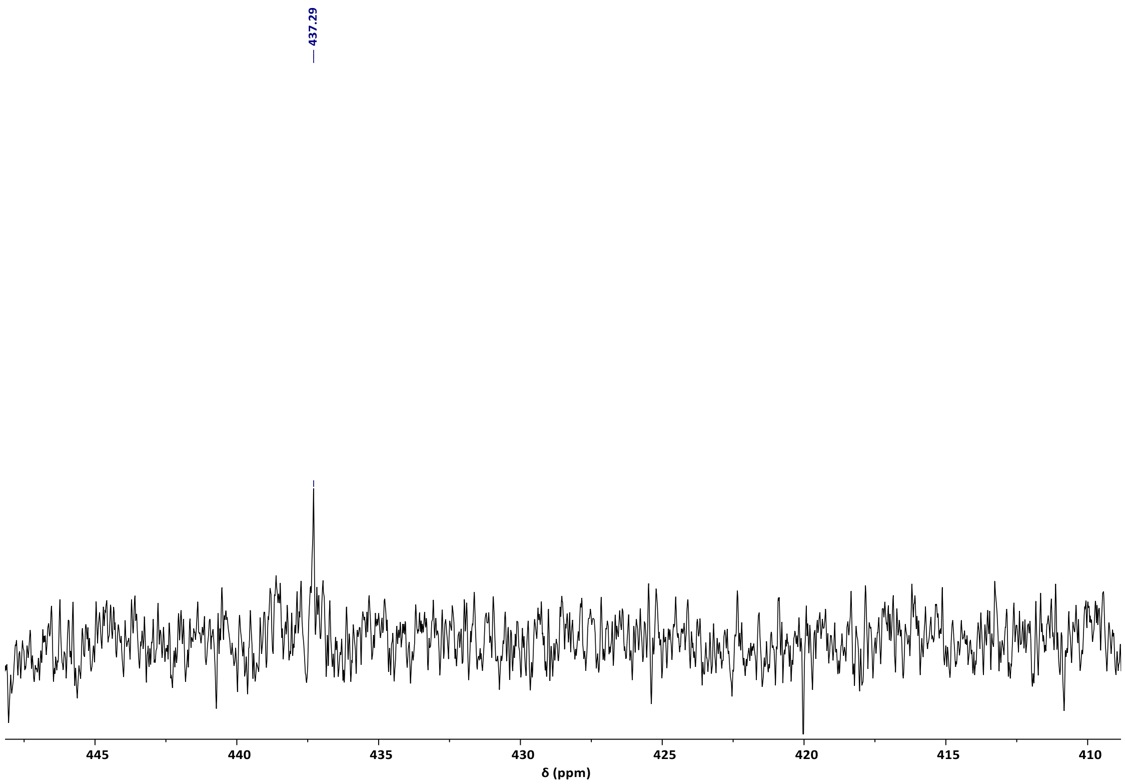

Figure S56. ^{77}Se NMR spectrum ($CDCl_3$, 114 MHz) of **16**.
